# Modelling acquired resistance to DOT1L inhibition exhibits the adaptive potential of *KMT2A*-rearranged acute lymphoblastic leukemia

**DOI:** 10.1186/s40164-023-00445-8

**Published:** 2023-09-22

**Authors:** Pauline Schneider, Nicholas T. Crump, Susan T.C.J.M. Arentsen-Peters, Alastair L. Smith, Rico Hagelaar, Fabienne R.S. Adriaanse, Romy S. Bos, Anja de Jong, Stefan Nierkens, Bianca Koopmans, Thomas A. Milne, Rob Pieters, Ronald W. Stam

**Affiliations:** 1 Princess Máxima Center for Pediatric Oncology, Utrecht, The Netherlands; 2 MRC Molecular Haematology Unit, Radcliffe Department of Medicine, MRC Weatherall Institute of Molecular Medicine, University of Oxford, Oxford, UK; 3 Hugh and Josseline Langmuir Centre for Myeloma Research, Centre for Haematology, Department of Immunology and Inflammation, Imperial College London, London, UK; 4 Oncode Institute, Utrecht, The Netherlands

## Abstract

In *KMT2A*-rearranged acute lymphoblastic leukemia (ALL), an aggressive malignancy, oncogenic KMT2A-fusion proteins inappropriately recruit DOT1L to promote leukemogenesis, highlighting DOT1L as an attractive therapeutic target. Unfortunately, treatment with the first-in-class DOT1L inhibitor pinometostat eventually leads to non-responsiveness. To understand this we established acquired pinometostat resistance in pediatric KMT2A::AFF1^*+*^ B-ALL cells. Interestingly, these cells became mostly independent of DOT1L-mediated H3K79 methylation, but still relied on the physical presence of DOT1L, HOXA9 and the KMT2A::AFF1 fusion. Moreover, these cells selectively lost the epigenetic regulation and expression of various KMT2A-fusion target genes such as *PROM1/*CD133, while other KMT2A::AFF1 target genes, including *HOXA9* and *CDK6* remained unaffected. Concomitantly, these pinometostat-resistant cells showed upregulation of several myeloid-associated genes, including *CD33* and *LILRB4/CD85k.* Taken together, this model comprehensively shows the adaptive potential of *KMT2A*-rearranged ALL cells upon losing dependency on one of its main oncogenic properties.

**Supplementary Information:**

The online version contains supplementary material available at 10.1186/s40164-023-00445-8.

## Background

Chromosomal translocations involving the *KMT2A* (*MLL*) gene constitute the cytogenetic hallmark of acute lymphoblastic leukemia (ALL) diagnosed in infants (< 1 year of age), giving rise to an aggressive malignancy with high relapse rates and low event-free survival (EFS) chances of 30–40% [1, 2]. Hence, currently available treatment regimens for *KMT2A*-rearranged infant ALL are inadequate and require more effective therapeutic options to improve clinical outcome.

*KMT2A* translocations result in the fusion of *KMT2A* to one of its many translocation partner genes, [3] generating chimeric transcripts encoding highly oncogenic KMT2A fusion proteins. Among infant ALL patients, *KMT2A* is most recurrently fused to either *AFF1* (*AF4*), *MLLT1* (*ENL*), or *MLLT3* (*AF9*) [3]. Functionally, wild-type *KMT2A* plays an essential role in definitive hematopoiesis [4] regulating gene expression through histone 3 lysine 4 (H3K4) methyltransferase activity, mediated by its Su(Var)_3–9_, Enhancer-of-zeste, Trithorax (SET) domain [5]. In contrast, KMT2A fusion proteins lose their SET domain, but instead recruit the histone 3 lysine 79 (H3K79) methyltransferase DOT1L through binding motifs encoded by the translocation partner genes [6–8]. Binding of DOT1L to KMT2A fusion proteins causes inappropriate H3K79 hypermethylation at KMT2A target genes, leading to an altered transcriptomic landscape that strongly favors leukemia development [6–9].

Interestingly, the mutational landscape of *KMT2A*-rearranged infant ALL is remarkably silent, with only 1.3–2.5 leukemia-specific, non-silent mutations in the dominant clone per patient, [10, 11] suggesting that the *KMT2A* translocation may well be the sole oncogenic lesion driving this aggressive type of leukemia [12]. Therefore, targeting DOT1L, through which KMT2A fusion proteins exert their oncogenic effects, represents an attractive therapeutic strategy. Accordingly, the development of the DOT1L inhibitor EPZ004777 and its successor EPZ5676 (pinometostat) [13, 14] were expected to become key to successful treatment of *KMT2A*-rearranged acute leukemias. However, despite promising preclinical results, subsequent clinical trials revealed that good initial responses in patients treated with pinometostat readily led to non-responsiveness due to acquired resistance and poor pharmacokinetics (PK) [15, 16]. Previously, it was reported that acquired resistance to pinometostat in *KMT2A*-rearranged acute leukemic cell lines is associated with enhanced drug efflux mediated by the elevated expression of the multidrug resistance transporters ABCB1 and ABCG2 [17].

Despite this, targeting DOT1L remains a promising avenue for treating *KMT2A* rearranged leukemias, and novel small-molecule DOT1L inhibitors with improved PK profiles have already been identified [18, 19]. For future drug development, more needs to be understood about exactly how DOT1L contributes to leukemogenesis, the role of its enzymatic methyltransferase activity, and how leukemias might develop resistance. Understanding these issues could not only impact the development of novel DOT1L inhibitors but could also be essential for better understanding the activity of novel compounds designed to target similar pathways. Therefore, following up on the study by Campbell and colleagues, [17] we here established and extensively characterized a model of acquired resistance to DOT1L inhibition in *KMT2A*-rearranged ALL cells.

## Methods

### Cell line models

The *KMT2A::AFF1*^*+*^ B-cell precursor ALL cell lines used are SEM, (DSMZ, cat.nr. ACC 546), and RS4;11, (ATCC; cat.nr. CRL-1873). Culture conditions are described in detail in the supplemental methods.

### Establishment of acquired pinometostat resistance in SEM and RS4;11 cells

SEM and RS4;11 cells were cultured in the presence of gradually increasing concentrations (ranging from 1 to 100 µM) of the DOT1L inhibitor pinometostat (EPZ5676, Selleckchem), for 14 weeks. For assessment of the in vitro response to pinometostat, cells were cultured in the absence of pinometostat for a few passages before exposing the cells to six concentrations (ranging from 0 to 100 μm), of pinometostat for 14 days. Trypan blue exclusion counts were used to calculate the inhibitory pinometostat concentration to 50% of the leukemic cells (i.e., IC_50_ value). *p* values were determined by ratio paired t-test using four biological replicates, mean with range.

### Immunoblotting

The presence of histone modifications and the levels of protein expression were determined by immunoblot analysis, as described in the supplemental methods.

### RNA sequencing (RNA-seq) and chromatin immunoprecipitation sequencing (ChIP-seq)

RNA- and ChIP-sequencing was performed on a NextSeq® 500 System (Illumina®). Experimental procedures and analyses are described in the supplemental methods.

### Assay for transposase-accessible chromatin sequencing (ATAC-seq)

ATAC sequencing was outsourced to Active Motif (ATACseq Service: https://www.activemotif.com/catalog/1233/atac-seq-service) to identify regions that have open or accessible chromatin states, as described in the supplemental methods.

### Flow cytometry (FACS) analysis

Details of FACS analysis are described in the supplemental methods.

### RNA interference

To transiently induce mRNA knockdowns, leukemic cells were electroporated in the presence of 500 nM of small-interfering RNAs (siRNAs) directed against the mRNA of selected target genes, as described previously [20], and as described in the supplemental methods.

### Quantitative reverse-transcription PCR analysis

RNA, isolated using the RNeasy Mini Kit (QIAGEN), was reverse transcribed and the obtained cDNA was used for quantitative reverse-transcription PCR (qRT-PCR) analysis as described previously [21], and as described in the supplemental methods.

### Cell viability assays and high-throughput drug screening

Cell viability assays were performed using flow cytometry and 7-AAD viability dye (BioLegend) to discriminate between viable and dead cells. Further details on the cell viability assays and high-throughput drug screens are described in the supplemental methods.

### Statistical analysis

Statistical significance of independent experimental replicates in graphs were determined by two-sided Student’s t-tests. All statistical analyses were conducted using GraphPad Prism8, version 8.3.4. *p* < 0.05 was considered statistically significant.

## Results

### Establishment of acquired resistance to DOT1L inhibition in *KMT2A*-rearranged ALL cells

**Fig. 1:**
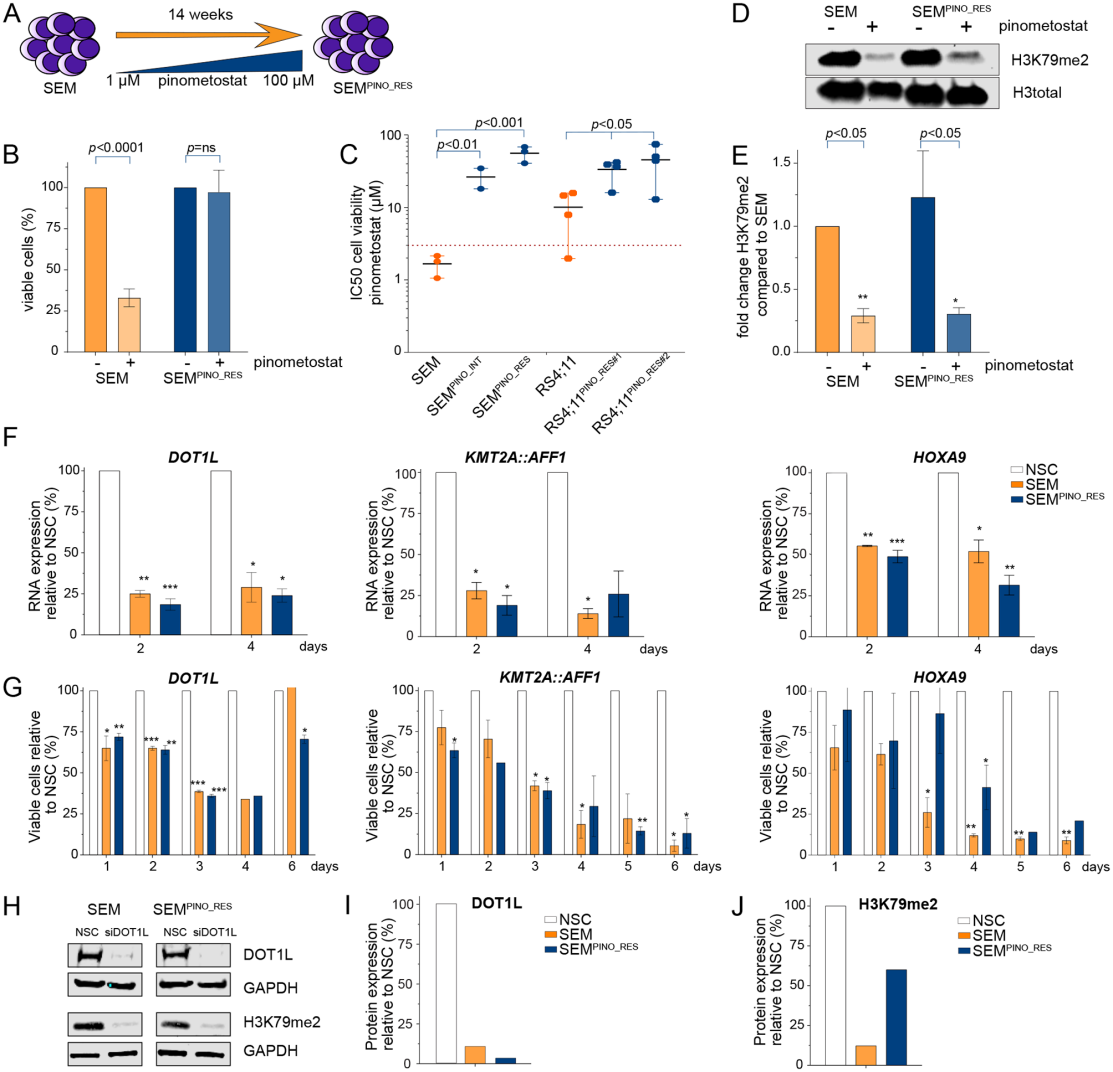
Establishment of acquired resistance to DOT1L inhibition in *KMT2A*-rearranged ALL cells. **A**. Graphic overview of acquired resistance induction to DOT1L inhibition in KMT2A::AFF1 + B-cell ALL (SEM) leading to pinometostat-resistant cells (SEM^PINO_RES^). **B**. Viable cell percentage of SEM and SEM^PINO_RES^ cells in the absence (-) or presence (+) of 50µM pinometostat for 7 days, normalized to cells cultured without pinometostat. Data present the mean +/- the standard deviation (SD) derived from 2 biological replicates. **C**. IC50 values of viable cells of the indicated cell line models determined using six drug concentrations (0-100 µM) for 14 days. The data illustrates the mean +/- SD from 4 biological replicates, each comprising 3 technical replicates. **D**. Immunoblot images of H3K79me2 and total histone H3 in SEM and SEM^PINO_RES^ cells cultured with or without 50 µM pinometostat for 7 days. **E.** Quantification of H3K79me2 protein expression using densitometry analysis normalized against total histone H3 expression. Data represent fold-changes normalized against untreated SEM cells for 2 biological replicates. **F.** mRNA expression of DOT1L, KMT2A:AFF1, and HOXA9 determined by qRT-PCR analysis, and **G**. viable cell percentage in SEM and SEM^PINO_RES^ at day 2 and day 4 after siRNA-mediated knockdown (KD) relative to non-silencing controls (NSCs). Data of 2 biological replicates ± SD, *p < 0.05, **p < 0.005, ***p < 0.0005, ****p < 0.0001. **(H)** Immunoblot images of DOT1L, H3K79me2, and GAPDH protein expression in SEM and SEM^PINO_RES^ cells at day 4 following siRNA-mediated KD of DOT1L, and corresponding quantification of **(I)** DOT1L or **(J)** H3K79me2 protein expression relative to GAPDH using densitometry analysis. Differences were statistically evaluated using unpaired t-tests

To induce acquired resistance to DOT1L inhibition, the *KMT2A::AFF1*^+^ ALL cell line SEM [22] was exposed to increasing concentrations of the first-in-class DOT1L inhibitor pinometostat for 14 weeks (Fig. 1A). Next, cells were cultured in the absence of drug before evaluating potential changes in pinometostat-induced cytotoxicity. Exposure of maternal SEM cells to 50 µM pinometostat for 1 week reduced the percentage of viable cells to ~ 35%.

In contrast the viability of SEM cells that underwent prolonged exposure to increasing pinometostat concentrations was hardly affected (Fig. 1B). This pinometostat-resistant daughter line, designated as SEM^PINO_RES^, revealed a 34-fold higher 14-day-IC_50_ value as compared to maternal SEM cells (Fig. 1C), indicating that SEM^PINO_RES^ became highly resistant. An additional model of intermediate resistance was established in SEM cells (i.e., SEM^PINO_INT^) by prolonged exposure to 4.5 µM pinometostat for 7 weeks, leading to a 16-fold higher IC_50_ (Fig. 1C).

Similar to SEM^PINO_RES^, we also induced pinometostat resistance in the *KMT2A::AFF1*^*+*^ ALL cell line RS4;11. With a mean 14-day-IC_50_ value of ~ 10 µM, representing pinometostat concentrations well above maximum achievable plasma levels in pinometostat-treated patients, [15, 16, 23] maternal RS4;11 is more resistant than SEM (Fig. 1C). Two emerging pinometostat-resistant RS4;11 daughter lines, i.e., RS4;11^PINO_RES#1^ and RS4;11^PINO_RES#2^, showed IC_50_ values of 33–45 µM (Fig. 1C).

To assess the inhibitory effects of pinometostat on DOT1L-mediated methyltransferase activity, the levels of H3K79 di-methylation (H3K79me2) were determined by immunoblot analysis. Interestingly, the levels of H3K79me2 in SEM and SEM^PINO_RES^ were comparable and pinometostat was able to substantially reduce the levels of H3K79me2 equally in both cell lines (Fig. 1D-E). Hence, despite persistent inhibition of DOT1L-mediated H3K79 methylation, cell viability in SEM^PINO_RES^ is no longer affected, suggesting that these cells became largely independent of H3K79 methylation induced by DOT1L.

Next, we assessed whether changes in global histone modifications had occurred between SEM and SEM^PINO_RES^. For this we used Mod Spec®, a mass spectrometry-based measurement for the relative abundance of over 80 distinct histone marks. This analysis confirmed no differences in the levels of H3K79 mono-, di-, and tri-methylation (i.e., H3K79me1, H3K79me2, and H3K79me3, respectively) between SEM and SEM^PINO_RES^, and showed equal reduction of these histone marks upon pinometostat exposure (Figure S1). Moreover, these data demonstrated that the global landscape of histone modifications between SEM cells and SEM^PINO_RES^ largely remained similar. The only histone modification that is downregulated in response to pinometostat exposure appeared to be H3K79 methylation, demonstrating the specificity of this agent.

In *KMT2A*-rearranged acute leukemias, the KMT2A fusion protein is considered to be the main oncogenic driver and loss of DOT1L was shown to specifically decrease *KMT2A* fusion-driven transcriptional programs, including the expression of *HOXA9.* [7] Therefore, we asked whether acquired resistance to DOT1L inhibition was accompanied by an altered dependency on DOT1L, KMT2A::AFF1 and/or HOXA9. Therefore SEM and SEM^PINO_RES^ cells were subjected to siRNA-mediated knockdown of these genes, resulting in significant reductions in mRNA expression of ~ 75–80% for *DOT1L* and *KMT2A::AFF1*, and ~ 50–65% for *HOXA9*, relative to non-silencing controls (NSCs) (Fig. 1F). Validation on the protein level confirmed a reduction of DOT1L of ~ 90% in SEM and of > 90% in SEM^PINO_RES^ (Fig. 1H,I). Knockdown of DOT1L was accompanied by a reduction of H3K79me2 of ~ 90% in SEM, and of ~ 40% SEM^PINO_RES^ (Fig. 1H, J). Surprisingly, knockdown of *DOT1L* and *KMT2A::AFF1* resulted in similar reductions in cell viability in both SEM and SEM^PINO_RES^. For HOXA9 suppression the effects on cell viability in SEM^PINO_RES^ appeared to be somewhat delayed (Fig. 1G). This suggests that although SEM^PINO_RES^ cells became less sensitive to inhibition of H3K79 methylation in terms of leukemic cell survival, these cells remained dependent on the physical presence of proteins known to be important in *KMT2A*-mediated leukemogenesis, including DOT1L.

### Acquired resistance to DOT1L inhibition leads to selective loss of KMT2A-fusion driven gene expression

**Fig. 2:**
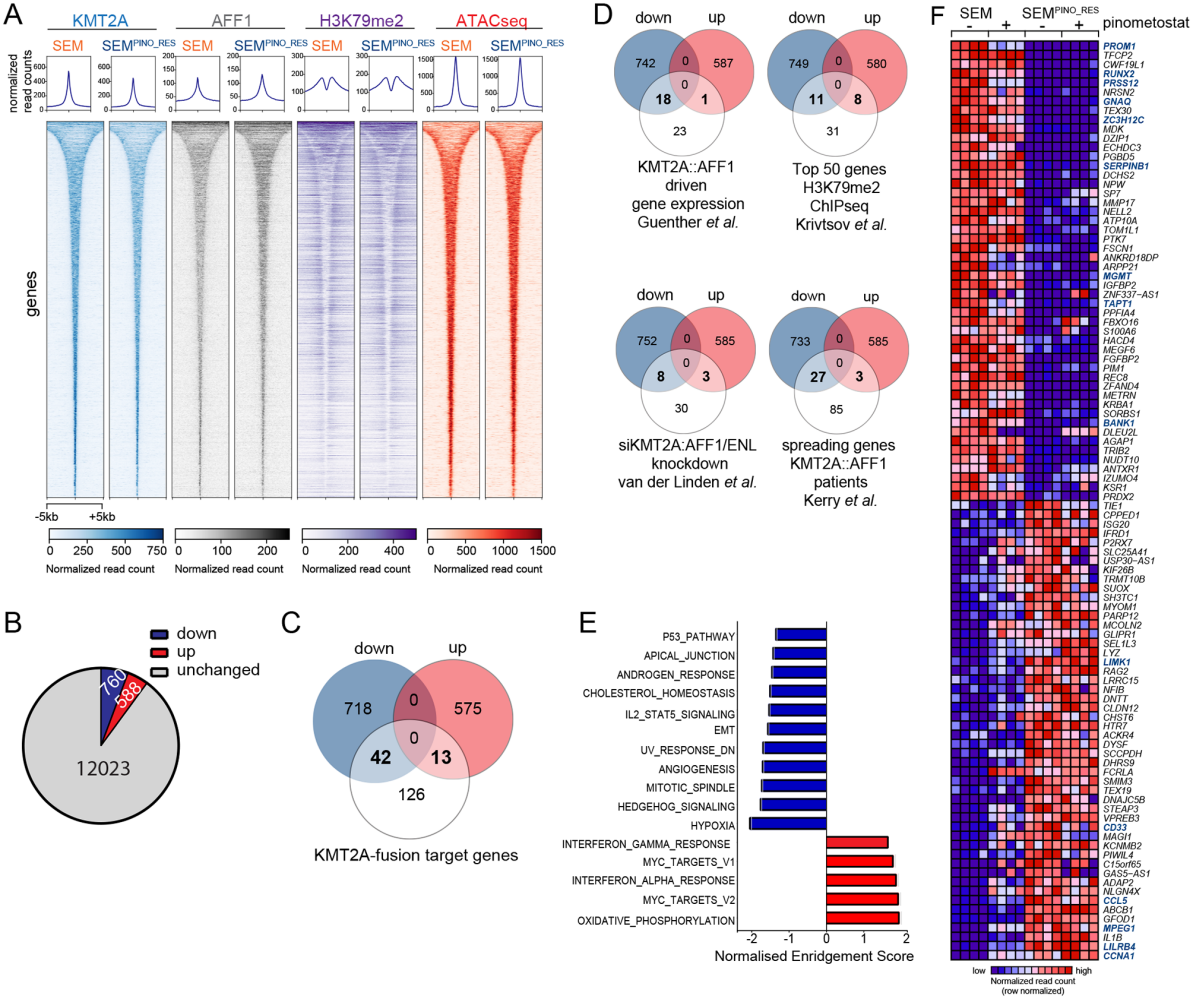
Characterization of SEM^PINORES^ by RNA-, ATAC-, and ChIP-sequencing. **A.** Heatmap showing ChIP-seq reads of KMT2A, AFF1, H3K79me2, and ATAC-seq reads at all KMT2A::AFF1 binding sites in SEM cells as well as SEM^PINO_RES^ at the same location, ranked by peak width. Scale bar represents normalized read count. **B**. Pie chart showing the number of genes for which the expression was significantly (i.e., at false discovery rate (FDR) adjusted *p*-values of < 0.05) downregulated (blue), upregulated (red), or remain unchanged (gray) between SEM^PINO_RES^ in comparison to SEM (RNA-seq data; n = 4 biological replicates/sample). **C.** Venn diagram showing the overlap of downregulated (blue) or upregulated (red) putative KMT2A-fusions target genes (n = 181) (white) in SEM^PINO_RES^ compared to SEM. The putative KMT2A fusion target genes in this figure comprise the combination of genes identified by four independent studies [9, 20, 24, 25], and **D.** similar Venn diagrams are presented using the KMT2A fusion target genes from each individual study. **E.** Forest plot showing hallmark gene sets that were positively or negatively enriched in Geneset Enrichment Analysis (GSEA), based on the Normalized Enrichment Score (NES). **F.** Heatmap showing the most positively enriched and significantly upregulated genes (n = 50) as well as the most negatively enriched and significsntly downregulated genes (n = 50) in SEM^PINO_RES^ as determined by GSEA. Data shown represents normalized RNA-seq counts in SEM and SEM^PINO_RES^ cells cultured for 7 days in either the absence (-) or presence (+) of 50 µM pinometostat of n = 4 biological replicates

Next, we performed RNA- and ChIP-seq for KMT2A, AFF1, H3K4me3, H3K79me2, and H3K27ac, as well as ATAC-seq on SEM and SEM^PINO_RES^ cells cultured in both the absence and presence of 50 µM pinometostat for 7 days. Interestingly, as assessed by ChIP-seq, there are very few observable differences in the global profiles of KMT2A, AFF1 or H3K79me2 as well as ATACseq profiles between SEM or SEM^PINO_RES^, suggesting that acquired pinometostat resistance does not lead to obvious global changes in open chromatin (Fig. 2A). Upon analyzing gene expression profiles, it became apparent that in the absence of pinometostat, there was a noteworthy decrease in the expression of 760 out of the 13,371 genes expressed (5.7%), while 588 genes (4.4%) exhibited an increase in expression in SEM^PINO_RES^ cells in comparison to SEM cells (Fig. 2B, Table S1). The differences in gene expression patterns triggered by pinometostat were relatively less prominent between the two cell line models (Figure S2, Table S1). In the presence of pinometostat in the original SEM cells, 670 genes (5.0%) revealed a significant decrease in expression, and 596 genes (4.5%) demonstrated a notable increase in expression, when compared to untreated SEM cells. Conversely, in the presence of pinometostat in SEM^PINO_RES^ cells, 208 genes (1.6%) were significantly downregulated, while 388 genes (2.9%) were significantly upregulated compared to untreated SEM^PINO_RES^ cells. Interestingly, a considerable number of genes reported to represent potential target genes of KMT2A fusion proteins [9, 20, 24, 25] were significantly downregulated in SEM^PINO_RES^ cells (Fig. 2C, Table S1). Approximately half of the KMT2A::AFF1 target genes identified by Guenther et al. [24] were downregulated in SEM^PINO_RES^ (Fig. 2D, Table S1), as well as a quarter of the top 50 genes associated with H3K79 methylation in *KMT2A*-rearranged acute leukemia patient samples as identified by Krivtsov et al. [9].

Likewise, ~ 25% of the genes we previously reported to be differentially expressed in response to siRNA-mediated repression of *KMT2A::AFF1* and *KMT2A:MLLT1* in *KMT2A*-rearranged ALL cells, [20] as well as a fourth of the genes reported to display binding of KMT2A:AFF1 that spreads beyond the gene promoter and well into the gene body as recently identified in SEM cells by Kerry et al., [25] were downregulated in SEM^PINO_RES^ (Fig. 2D, Table S1). Thus, acquired resistance to DOT1L inhibition leads to selective (or partial) loss of KMT2A-fusion driven gene expression. To explore biological pathways potentially affected by acquired resistance to DOT1L inhibition, we performed Gene Set Enrichment Analysis (GSEA) on all RNA-seq data, and identified various hallmark gene sets to be significantly (nominal *p*-value < 0.05) modulated in SEM^PINO_RES^. These included the upregulated gene sets ‘MYC targets v1 and v2’ and ‘Oxidative Phosphorylation’, as well as downregulated gene sets such as ‘p53 pathway’ (i.e., DNA damage response genes), ‘epithelial-to-mesenchymal transition (EMT)’ and ‘hypoxia’ (Fig. 2E).

**Fig. 3:**
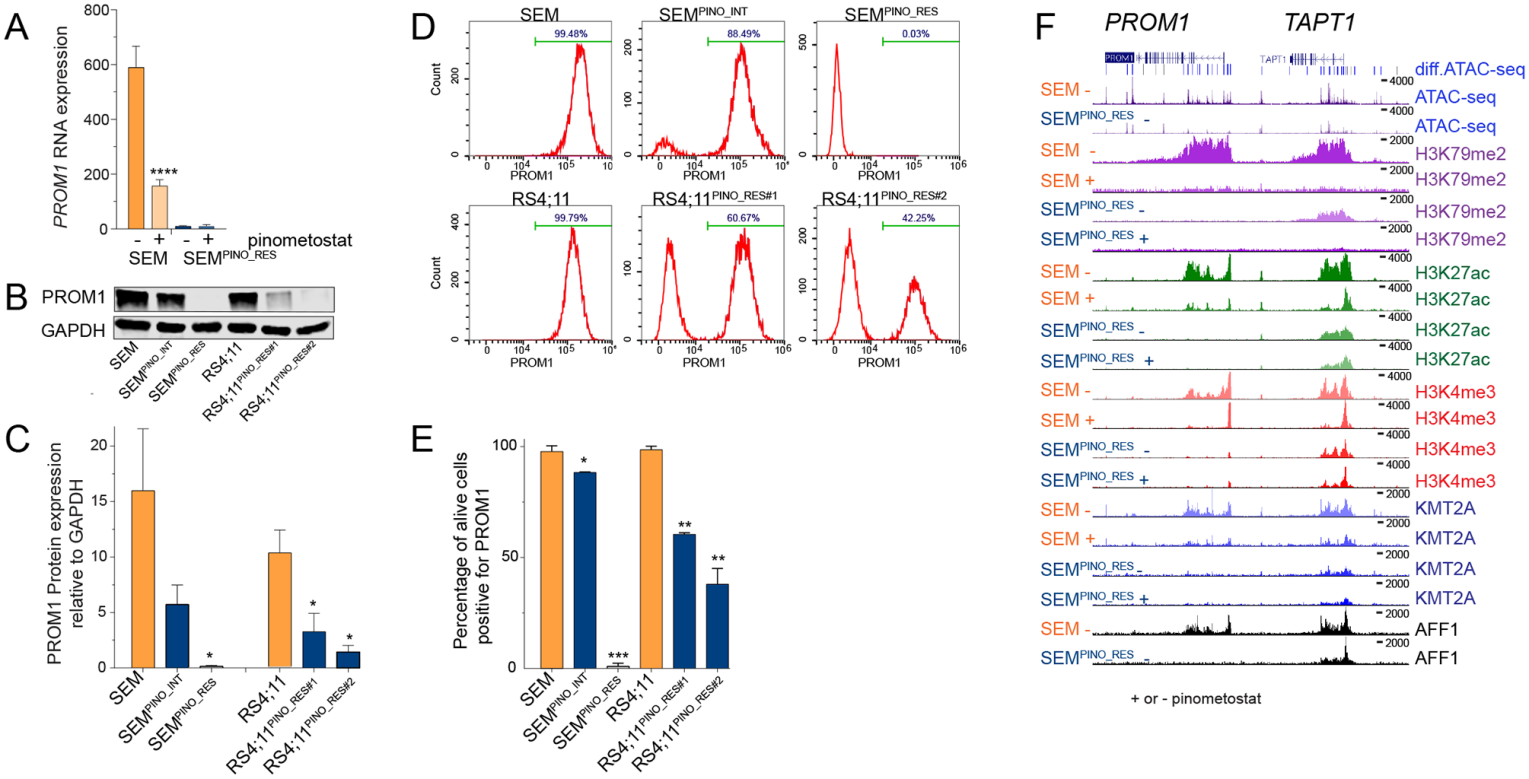
Acquired resistance to DOT1L inhibition leads to selective loss of KMT2A-fusion driven PROM1 expression. **A.***PROM1* mRNA expression in SEM and SEM^PINO_RES^ cells cultured for 7 days in the absence (-) or presence (+) of 50 µM pinometostat, as determined by RNA-seq. Values indiacte normalized counts with SD derived from four biological replicates for each cell line and condition. ****p < 0.0001. **B.** Western blot images of PROM1 and GAPDH protein levels in indicated cell line models, and **C.** quantification of PROM1 expression relative to GAPDH by densitometry analysis. Values indicate mean ± SD PROM1 protein expression as determined in two biological replicates. *p < 0.05. **D.** Histograms showing the counts of viable cells positive for PROM1/CD133 of indicated cell line models, as determined by flow cytometry (FACS) analysis, and **E.** Quantification of PROM1/CD133 expression presented as the mean ± SD as determined by two independent FACS experiments. *p < 0.05, **p < 0.005. **F.** Differences in chomatin accessibility at the *PROM1* and *TAPT1* gene locus between SEM^PINO_RES^ and SEM cells as determined by ATAC-sequencing of two biological replicates (on top). Vertical blue lines indicate significant decreases of chromatin accessibility in SEM^PINO_RES^ cells, whereas grey lines indicate equal chromatin accessibility in both SEM^PINO_RES^ and SEM. The ATAC-sequencing results are followed by ChIP-sequencing tracks of the same locus showing the distribution of H3K79Me2, H3K27Ac, H3K4Me3, KMT2A in SEM and SEM^PINO_RES^ cells cultered for 7 days in either the absence (-) or presence (+) of 50 µM pinometostat. Differences were statistically evaluated using unpaired t-tests

Examination of the genes most prominently enriched (n = 50) and the genes most notably under-represented (n = 50) in our GSEA data revealed *PROM1* to be the most downregulated gene (GSEA score of -4,83) and *CCNA1* the most positively enriched gene (GSEA score of 3,66) in SEM^PINO_RES^ cells (Fig. 2F). Both genes represent putative KMT2A fusion targets epigenetically marked by H3K79 methylation and have shown to be highly and specifically expressed in *KMT2A*-rearranged ALL [9]. *PROM1* encodes a transmembrane glycoprotein (i.e., CD133) commonly regarded as a cancer stem cell marker [26–28] and reported to be an important target of KMT2A::AFF1 [24, 29, 30]. *PROM1* is robustly expressed in SEM cells but readily downregulated during pinometostat exposure, whereas *PROM1* expression was nearly absent in SEM^PINO_RES^ (Fig. 3A). Analysis at protein level by immunoblot and FACS confirmed the complete loss of PROM1/CD133 in SEM^PINO_RES^, while in SEM^PINO_INT^ PROM1/CD133 was still present in in ~ 88% of the cells (Fig. 3B-E). This indicates that PROM1/CD133 expression is gradually lost from the population after prolonged pinometostat exposure. Similarly, RS4;11 cells firmly express PROM1/CD133, which was markedly reduced in both RS4;11^PINO_RES#1^ and RS4;11^PINO_RES#2^ (Fig. 3B-E).

ChIP-sequencing data for KMT2A, AFF1, H3K4me3, H3K79me2, and H3K27ac showd that upon pinometostat exposure, SEM cells display a clear reduction of KMT2A binding in *PROM1*, which was accompanied by strong reductions of the levels of H3K79me2, H3K4me3, and H3K27ac at the *PROM1* gene as well as at its enhancer *TAPT1* [29] (Fig. 3F). In untreated SEM^PINO−RES^ cells the *PROM1* locus is completely devoid of KMT2A, AFF1, H3K79me2, H3K4me3, and H3K27ac, suggesting that this gene is no longer being regulated by KMT2A::AFF1 and subsequent DOT1L-mediated H3K79 methylation. Also, ATAC-sequencing clearly revealed decreased chromatin accessibility at the promoter and enhancer of *PROM1* in SEM^PINO−RES^ (Fig. 3F). Interestingly, at the *TAPT1* locus in these same SEM^PINO−RES^ cells KMT2A and AFF1 binding as well as the levels of H3K4me3, H3K79me2, and H3K27ac to some extent remained intact (Fig. 3F).

In addition to *PROM1*, the expression of other putative KMT2A::AFF1 target genes, including *RUNX2*, *PRSS12, ZC3H12, SERPINB1*, *GNAQ* and *BANK1* were severely downregulated in SEM^PINO−RES^ with a logFC of > 3-fold (Fig. 2F and Figure S3A) and exhibited similar patterns of RNA-, ChIP-, and ATAC-seq as observed for *PROM1* (Figure S3B), indicating their dependence on KMT2A::AFF1-mediated epigenetic control. In contrast, at *SERPINB1* only moderate levels of H3K79me2 were observed, accompanied by rather weak binding of KMT2A and absence of AFF1, suggesting that this gene may not necessarily be regulated KMT2A fusion proteins and DOT1L (Figure S3A, B),).

Collectively, these data demonstrate that a selection of known KMT2A::AFF1 target genes that are responsive to pinometostat-mediated DOT1L inhibition in SEM cells are relieved from the epigenetic control of KMT2A::AFF1 and become transcriptionally silenced in SEM^PINO_RES^ cells.

**Fig. 4:**
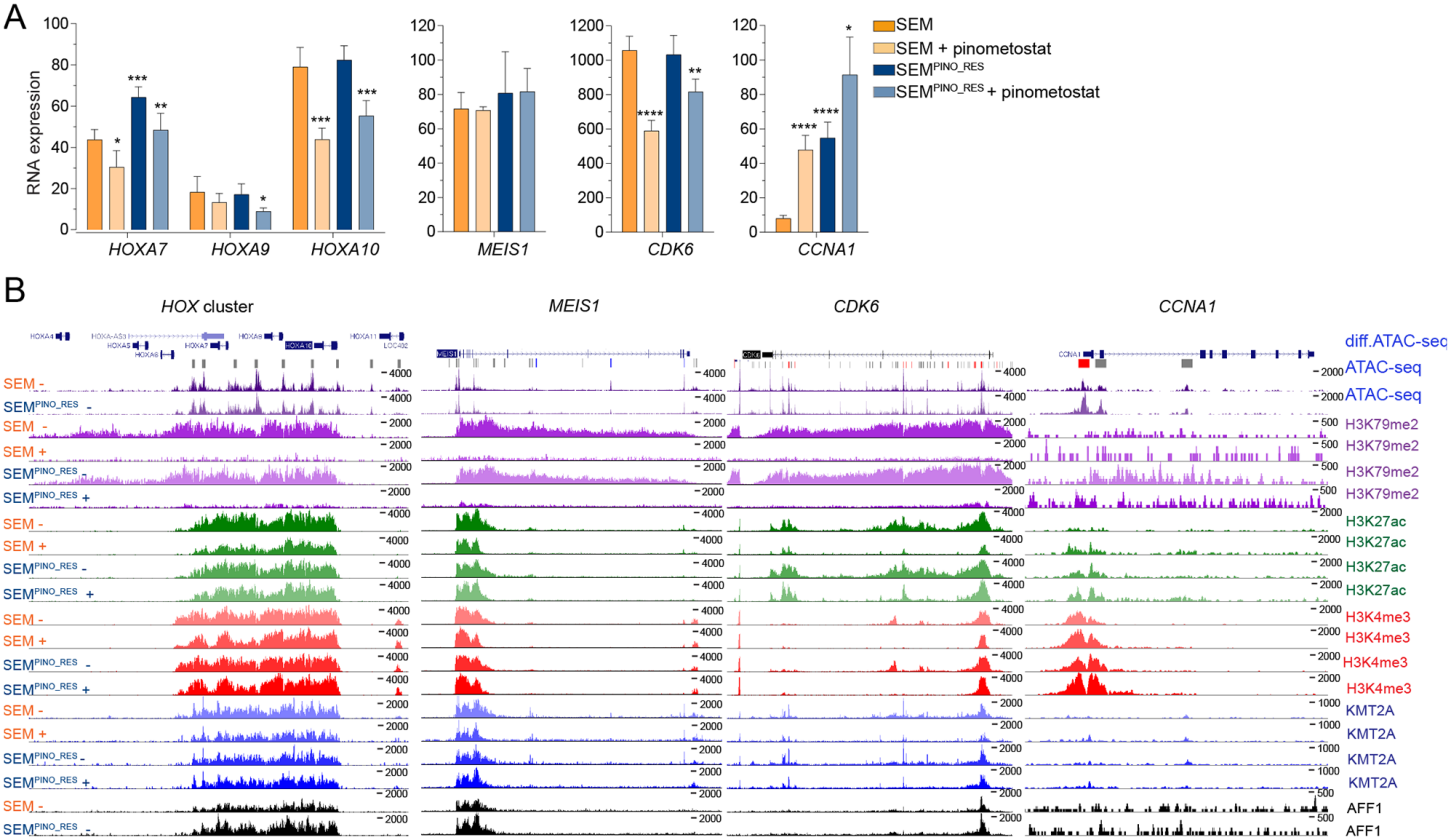
Unaltered or upregulated gene expression of KMT2A-fusion targets after acquired resistance to DOT1L inhibition. **A**. *HOXA7, HOXA9, HOXA10*, *MEIS1*, *CKD6*, and *CCNA1* mRNA expression SEM and SEM^PINO_RES^ cells cultured for 7 days in the absence (-) or presence (+) of 50 µM pinometostat, as determined by RNA-seq. Values indiacte normalized counts with standard deviation (SD) derived from four biological replicates for each cell line and condition. Differences in expression were statistically evaluated using unpaired t-tests; * *p* < 0.05, * *p* < 0.05, ** *p* < 0.005, *** *p* < 0.0005, **** *p* < 0.0001. Differences in chomatin accessibility at the *HOXA*, *MEIS1*, *CKD6*, and *CCNA1* gene loci between SEM^PINO_RES^ and SEM cells as determined by ATAC-sequencing by two biological replicates (on top). Vertical blue lines indicate significant decreases of chromatin accessibility in SEM^PINO_RES^ cells, whereas grey lines indicate equal chromatin accessibility in both SEM^PINO_RES^ and SEM. Red lines indicate significant increases in chromatin accessibility in SEM^PINO_RES^. Below the ATAC-sequencing data, ChIPseq tracks showning the presence of H3K79Me2, H3K27Ac, H3K4Me3, KMT2A, and AFF1 at the corresponding gene loci in SEM and SEM^PINO_RES^ cells cultured for 7 days in the abscence (-) or presence (+) of 50 µM pinometostat

Following previous evidence on the role of DOT1L in *HOXA* gene expression in KMT2A::AFF1^+^ ALL cells, [9, 24] we examined the *HOXA* locus and found that *HOXA9* and *HOXA10* were expression at comparable levels in both SEM and SEM^PINO_RES^ (Fig. 4A). Inhibition of DOT1L-mediated H3K79 methylation by pinometostat resulted in moderately decreased expression of *HOXA9*, *HOXA7*, and *HOXA10*, while the levels of H3K27ac, H3K4me3, KMT2A, and AFF1 remained unchanged in both cell lines (Fig. 4A,B). Similar patterns were found for other KMT2A-fusion target genes, including *CDK6*, involved in cell proliferation in *KMT2A* rearranged ALL [20] (Fig. 4A,B and S4A-C). The expression of *MEIS1*, which encodes a required co-factor of HOXA9-driven leukemogenesis, [9, 24, 31, 32] remained unaltered upon pinometostat exposure despite reductions in the levels of H3K79me2 in both SEM and SEM^PINO_RES^ (Fig. 4A,B and S4A,B,C). Collectively, this indicates that a subset of KMT2A-fusion target genes continued to be regulated by DOT1L in pinometostat-resistant ALL cells, while a separate group of genes showed no transcriptional response to the inhibition of DOT1L-mediated H3K79 methylation.

Intriguingly, we also found the expression of some putative KMT2A-fusion target genes to be upregulated in SEM^PINO_RES^ in the absence of pinometostat (Figure S4D), including *HOXA7*, *NLGN4X*, *CCNA1*, *FCRLA*, *IL7R*, *LYN* and *FUT4* (Fig. 4A,B and Figure S4D,E).

### Upregulation of myeloid-associated gene expression in *KMT2A*-rearranged ALL cells upon acquired resistance to DOT1L inhibition

**Fig. 5:**
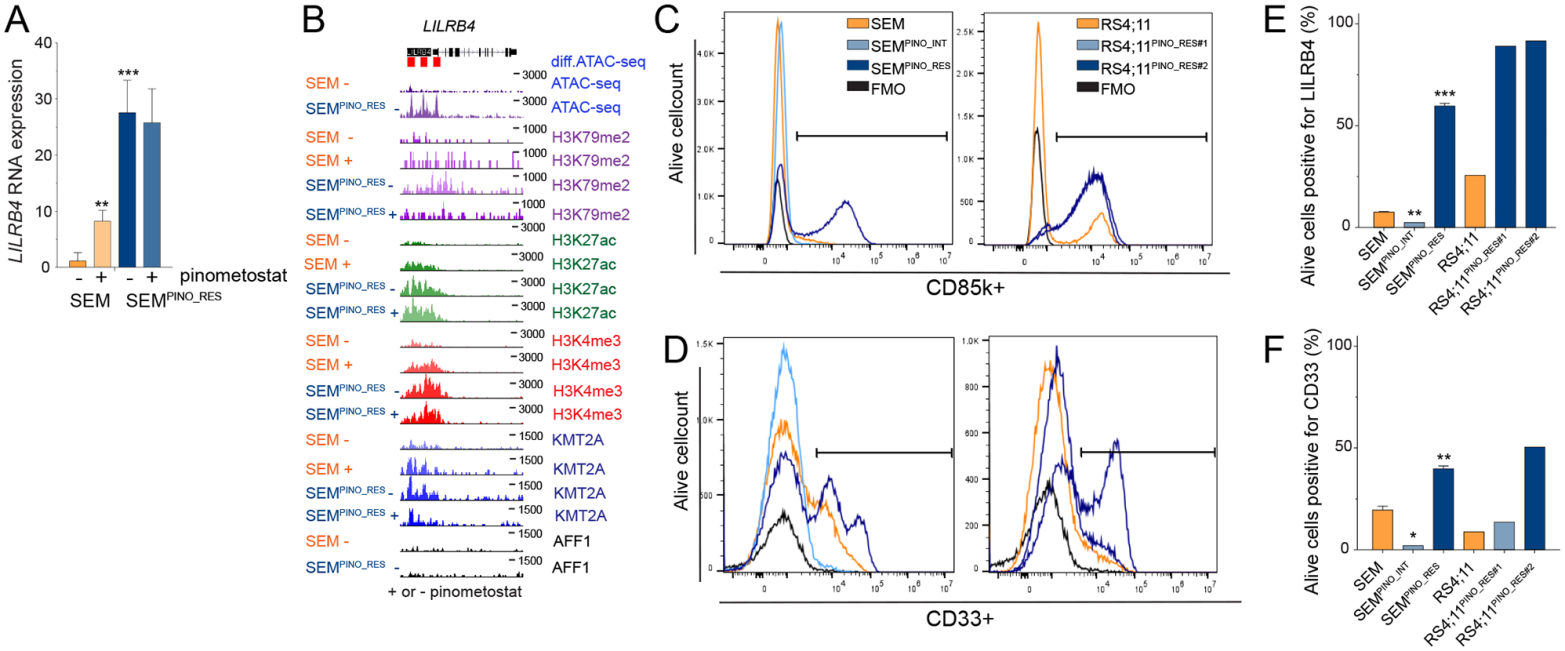
Upregulation of myeloid-associated CD85k/LILRB4 and CD33 expression during the acquirement of resistance to DOT1L inhibition. **A***LILRB4* mRNA expression in SEM and SEM^PINO_RES^ cells cultured for 7 days in the absence (-) or presence (+) of 50 µM pinometostat, as determined by RNA-seq. Values indiacte normalized counts with SD derived from four biological replicates for each cell line and condition. Differences in expression were statistically evaluated using unpaired t-tests; **p < 0.005, ***p < 0.0005. **B.** Differences in chomatin accessibility at the *LILRB4* gene locus between SEM^PINO_RES^ and SEM cells as determined by ATAC-sequencing by 2 biological replicates (on top). Red boxes indicate locations within the *LILRB4* gene locus of significantly increased of chromatin accessibility in SEM^PINO_RES^ as compared to SEM cells. In addtionm, ChIPseq tracks are presented showing the presence of H3K79Me2, H3K27Ac, H3K4Me3, KMT2A, and AFF1 at the same locus in and indicated cell line models cultured for 7 days in the absence (-) or presence (+) of 50 µM pinometostat. **C.** Histograms showing the counts of viable cells positive for CD85k/LILRB4 and **D.** CD33 protein surface expression of indicated cell line models, as determined by flow cytometry (FACS) analysis. Fluorescence Minus One (FMO) controls were used to determine the cut-off point for the positive cell population. **E.** Quantification of CD85k/LILRB4 and **F**. CD33 expression represented as the mean ± SD, determined through either one (RS4;11 cells) or two (SEM cells) independent FACS experiments, each involving biological replicates. Differences in expression were statistically evaluated using unpaired t-tests; *p < 0.05, **p < 0.005, ***p < 0.0005

Apart from differential gene expression of putative KMT2A::AFF1 target genes, our data also revealed changes in expression of genes not associated with KMT2A fusions and/or H3K79 methylation (Fig. 2F). One of the most enriched and upregulated genes upon acquired pinometostat-resistance according to our GSEA is *LILRB4* (Figs. 2F and 5A), encoding the monocytic differentiation marker CD85k [33–35]. *LILRB4* is hardly expressed in SEM cells but is moderately upregulated during pinometostat exposure and substantially expressed in SEM^PINO_RES^ cells (Fig. 5A). In SEM cells, pinometostat induced an increase of chromatin accessibility as well as an increase in the levels of H3K27ac and KMT2A binding at the *LILRB4* locus, yet no H3K79me2 or binding of AFF1 was detected, suggesting that upregulated of *LILRB4* expression is not dependent on DOT1L or KMT2A::AFF1 (Fig. 5B).

FACS analysis confirmed an increased population of ~ 60% in LILRB4/CD85k positive cells in SEM^PINO_RES^ compared to only ~ 7% in SEM (Fig. 5C,E). Counterintuitively, instead of an expected moderate increase in LILRB4/CD85k-positive cells, we found SEM^PINO_INT^ to have lost LILRB4/CD85k expression almost completely (Fig. 5C,E). In RS4;11 already 25% of the cells were positive for LILRB4/CD85k, which tremendously increased to approximately 90% of the cells in both RS4;11^PINO_RES^ daughter lines (Fig. 5C,E). Interestingly, apart from *LILRB4/CD85k*, we found additional myeloid-associated genes to be upregulated in SEM^PINO_RES^, including *CD33, CCL5*, *LIMK1, and MPEG1*, revealing similar patterns of RNA-, ChIP- and ATAC-seq as *LILRB4*, although less prominent (Fig. 2F, Figures S5A, S5B). CD33, commonly expressed in a subpopulation in *KMT2A*-rearranged infant ALL [36–38], serves as an important immunophenotypic marker for the characterization of pediatric acute myeloid leukemia (AML) by EuroFlow [39–42] and has been exploited as a therapeutic target for AML. In SEM a subpopulation of 20% of CD33-positive cells was identified, which was increased in SEM^PINO_RES^ to about 40%, yet CD33-positive cells again were largely absent in SEM^PINO−INT^ (Fig. 5D,F). Similarly, in RS4;11 a CD33-positive subpopulation of 9% was increased upon pinometostat resistance to 14% in RS4;11^PINO_RES#1^ and up to 50% in RS4;11^PINO_RES#2^ (Fig. 5D,F).

Together these data indicate that under prolonged pressure of DOT1L inhibition, *KMT2A*-rearranged ALL cells seem to initiate a reprogramming process that involves the acquisition (or selection) of myeloid-like characteristics.

### Drug screens reveal minimal cross resistance, and sensitization towards venetoclax after acquired pinometostat resistance

Finally, acquired pinometostat resistance led to the upregulation of the multidrug efflux pump ABCB1 (Fig. 2F and Figure S6A,B), associated with multidrug resistance and previously reported as the mechanism of pinometostat resistance in *KMT2A*-rearranged acute leukemia cell lines [17]. However, our data challenges the concept that elevated ABCB1 expression alone is the mechanism of resistance to DOT1L inhibition as reported previously [17]. Despite significant ABCB1 upregulation, we still observe comparable inhibition of H3K79 methylation in SEM and SEM^PINO_RES^ (Fig. 1D and E), indicating that the amount of pinometostat and/or its retention in SEM^PINO_RES^ cells is sufficient to effectively reduce H3K79me2 levels, overriding the impact of ABCB1 upregulation.

**Fig. 6:**
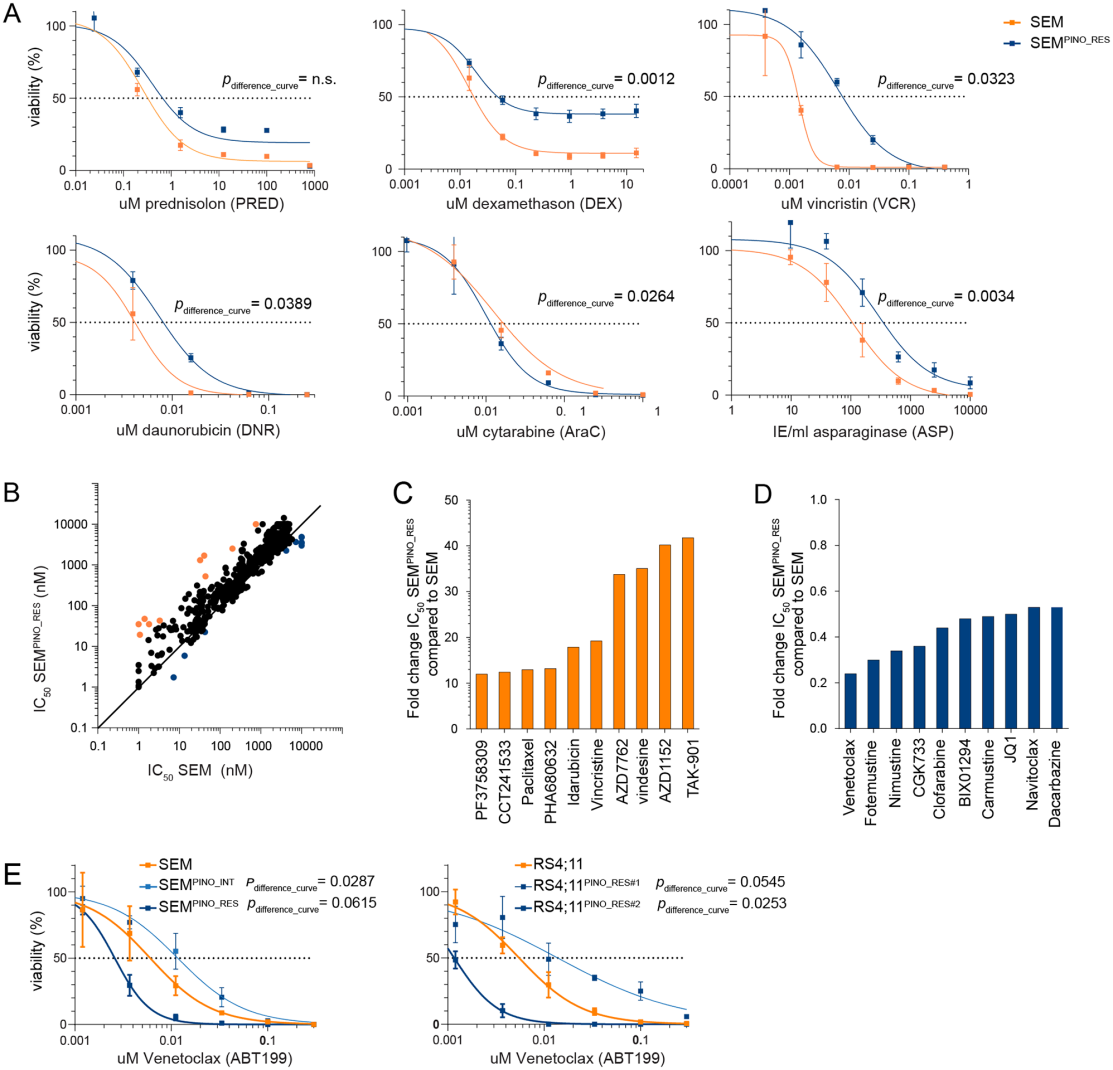
Moderate levels of cross-resistance and substantial sensitization towards venetoclax in pinometostat-resistant *KMT2A*-rearranged ALL cells. **A**. Drug response curves for prednisolone, dexamethasone, vincristine, daunorubicin, cladribine, cytarabine, and L-asparaginase as determined by 4-day MTT assays in SEM and SEM^PINO_RES^ cells with n = 4 biological replicates, each comprising n = 3 technical replicates, and presented as the mean ± standard error of the mean (SEM). **B**. Comparison of the IC_50_ in SEM and SEM^PINO_RES^ cells for a total of 679 compounds tested in drug library screens. **C**. Showing the top 10 agents with the highest fold-changes in IC_50_ values in SEM^PINO_RES^ as compared to SEM, indicating enhanced resistance in SEM^PINO_RES^ cells. **D.** The top 10 drugs with the lowest fold-changes in IC_50_ values in SEM^PINO_RES^ as compared to SEM, indicating enhanced sensitivity in SEM^PINO_RES^ cells. **E**. Drug response curves for venetoclax as determined by 4-day MTT assays in indicated cell lines models (in duplicate), presented as the mean ± standard error of the mean (SEM)

Since multidrug efflux pumps are associated with multidrug resistance [43, 44], we assessed whether SEM^PINO_RES^ cells had become more resistant to current chemotherapeutics for *KMT2A*-rearranged infant ALL [1, 2] and whether we could identify agents to which SEM^PINO_RES^ cells had become more sensitive by performing drug library screens (Table S2, Fig. 6A,B).

This revealed an increased resistance to the glucocorticoids dexamethasone and prednisolone (the liver-activated form of prednisone), vincristine, daunorubicin, and L-asparaginase, and increased sensitivity to cytarabine and for instance to the BCL-2 inhibitor venetoclax (Fig. 6A-E). Interestingly, cytarabine typically represents a drug commonly used in the treatment of myeloid leukemias, and the combination of venetoclax and cytarabine has successfully been tested in AML patients [45–47].

## Discussion

The currently accepted dogma of KMT2A-fusion driven leukemogenesis dictates the requirement of DOT1L-mediated activation of KMT2A target genes through H3K79 methylation [6, 9, 48, 49]. Therefore, targeting DOT1L [13, 18, 19] represents an attractive therapeutic option for patients diagnosed with *KMT2A*-rearranged acute leukemia, despite the first-in-class DOT1L inhibitor pinometostat showing dissatisfying results in adult patients [15]. While next generation DOT1L inhibitors with improved pharmacokinetic profiles are in development, [18, 19] we reasoned that the mechanisms by which *KMT2*A-rearranged acute leukemia cells evade DOT1L inhibition may provide novel insights into the biology of these unique malignancies. Therefore, and in a similar fashion as published by Campbell and colleagues [17], we efficiently induced acquired pinometostat resistance in various cell line models, demonstrating how readily *KMT2A*-rearranged acute leukemia cells become resistant to DOT1L inhibition. The study of Campbell et al., mainly focused on examples of possible mechanisms of pinometostat resistance including increased expression of drug efflux transporters and activation of the PI3K/AKT and RAS/RAF/MEK/ERK pathways [17]. In contrast, we here specifically focused on the behavior and epigenetic regulation of DOT1L-associated *KMT2A* fusion-driven target genes and on how the transcriptomic landscape changes in *KMT2A*-rearranged ALL cells that are able to evade leukemic cell death during prolonged inhibition of DOT1L-mediated H3K79 methylation.

In concordance with the previous finding by Campbell et al., we found increased expression of the multi-drug efflux pump *ABCB1* in our pinometostat-resistant SEM^PINO_RES^ cells. However, despite the elevated levels of *ABCB1* expression, pinometostat continued to inhibit H3K79 methylation in SEM^PINO_RES^. This strongly indicates that the increased levels of ABCB1 are insufficient to prevent pinometostat from exerting its inhibitory effects, and therefore cannot be the sole mechanism of acquired pinometostat resistance.

Interestingly, while cell viability of SEM^PINO_RES^ cells was no longer affected by pinometostat-induced inhibition of DOT1L-mediated H3K79 methylation, these cells remained dependent on the physical presence of DOT1L protein. This may indicate that recently described biological functions of DOT1L that are independent of H3K79 methylation [50–52] are also important for *KMT2A*-rearranged acute leukemia cells. Thus, in addition to its enzymatic methylatransferase activity, DOT1L clearly has a scaffold function in assembling transcriptionally competent complexes. Therefore, therapeutic degradation of DOT1L instead of solely inhibiting its catalytic activities might be beneficial in the treatment of *KMT2A*-rearranged acute leukemia.

Another intriguing aspect of our model of acquired pinometostat resistance is the observation that SEM^PINO_RES^ cells remained vulnerable to knockdown of the KMT2A::AFF1 fusion gene. This may suggest that inappropriate recruitment of DOT1L to loci otherwise not associated with H3K79me2 may not represent the sole KMT2A fusion-mediated attribute driving leukemogenesis and/or leukemia maintenance. If so, the identification of such DOT1L-independent oncogenic properties may well uncover important therapeutic targets and more effective treatment options for *KMT2A*-rearranged acute leukemias.

As shown, acquired resistance to pinometostat led to marked transcriptional downregulation of putative KMT2A-fusion target genes, which was accompanied by reductions in H3K79me2, as well as loss of binding of KMT2A and AFF1, and chromatin condensation at the corresponding loci. The complete loss of PROM1/CD133, which was shown to be transcriptionally regulated via KMT2A::AFF1-mediated H3K79me2/3 enhancer–promoter interactions, [29] in SEM^PINO_RES^ is highly remarkable, since the expression of PROM1/CD133 was reported to be essential for leukemic cell growth in *KMT2A*-rearranged ALL [29, 30]. Consequently, targeting PROM1/CD133-positive cells has been proposed as a therapeutic option for *KMT2A*-rearranged ALL, although the expression of PROM1/CD133 on both fetal and adult hematopoietic stem cells (HSCs) may compromise the specificity of such an approach [29, 30, 53, 54]. Moreover, PROM1/CD133 is expressed in most, but not all, *KMT2A*-rearranged acute leukemia patients, and its presence seems to reflect the immunophenotype and/or cell of origin of the leukemia, as HSCs and early progenitors typically express PROM1/CD133, while more differentiated B-cell progenitors do not [29, 54, 55]. As acquiring resistance to DOT1L inhibition was accompanied by a complete loss of PROM1/CD133 expression, this may suggest that prolonged exposure to pinometostat triggered the differentiation towards (or selection of) a more mature immunophenotype. On the other hand, our data revealed that prolonged exposure of *KMT2A*-rearranged ALL cells to pinometostat seems to initiate a reprogramming process that involves the acquirement (or selection) of myeloid-like characteristics. Co-expression of myeloid CD markers, including CD33, [36] represents a familiar phenomenon in *KMT2A*-rearranged infant ALL with prognostic relevance [37, 38]. Moreover, a recent single-cell multiomics study by Chen and co-workers revealed the presence of pre-existing lymphomyeloid primed progenitors and myeloid blasts in diagnostic samples derived from *KMT2A*-rearranged B-ALL patients [56]. From this perspective, prolonged inhibition of DOT1L seems to favor *KMT2A*-rearranged leukemia cells that completely lack PROM1/CD133 but do display LILRB4/CD85k and CD33 expression. Interestingly, both LILRB4/CD85k and CD33 are therapeutic targets in AML [57–63] and have shown potential as therapeutic vulnerabilities in *KMT2A*-rearranged ALL. Targeting LILRB4/CD85k with antibody-conjugates [57] or anti-LILRB4 CAR-T cells [64, 65] and/or CD33 with gemtuzumab ozogamicin, could prevent resistance to DOT1L inhibitors in *KMT2A*-rearranged ALL. Moreover, combining BCL-2 inhibition by venetoclax with agents targeting DOT1L, LILRB4/CD85k, and/or CD33 may enhance the efficacy of these drug combinations. Venetoclax was found to synergize with DOT1L inhibitors [25, 66] and is being evaluated in clinical trials for pediatric *KMT2A*-rearranged leukemias [67, 68].

Taken together, we present an in vitro model of acquired resistance to DOT1L inhibition in *KMT2A*-rearranged ALL, revealing selective loss of epigenetic regulation and gene expression of KMT2A-fusion target genes, accompanied by upregulation of myeloid-like characteristics. This study may not only impact the development of novel DOT1L inhibitors, but also reveal key characteristics of *KMT2A*-rearranged ALL cells that are able to evade therapy, providing therapeutic targets to prevent that.

### Electronic supplementary material

Below is the link to the electronic supplementary material.

Supplementary Material 1

## Data Availability

The cell lines resistant to DOT1L inhibitor pinometostat generated in this study is available from the lead contact upon request. Data and code availability: Sequencing data generated for this publication have been deposited in the Gene Expression Omnibus (GEO), accession GSE230807. Any additional information required to reanalyze the data reported in this paper is available from the lead contact upon request.
