# Supplementary material for: Modelling acquired resistance to DOT1L inhibition exhibits the adaptive potential of KMT2A-rearranged acute lymphoblastic leukemia
Source: Exp Hematol Oncol. 2023 Sep 22;12:81. doi: 10.1186/s40164-023-00445-8 (PMC10517487; doi:10.1186/s40164-023-00445-8)
Supplement: Supplementary file 1 — Supplementary Material 1 [file 40164_2023_445_MOESM1_ESM.pdf]

# Supplementals

## Modelling acquired resistance to DOT1L inhibition exhibits the adaptive potential of *KMT2A*-rearranged Acute Lymphoblastic Leukemia

Pauline Schneider[1], Nicholas T. Crump[2,3], Susan T.C.J.M. Arentsen-Peters[1], Alastair L. Smith[2], Rico Hagelaar[1,4], Fabienne R.S. Adriaanse[1], Romy S. Bos[1], Anja de Jong[1], Stefan Nierkens[1], Bianca Koopmans[1], Thomas A. Milne[2], Rob Pieters[1], Ronald W. Stam[1]

# Supplementary methods

### Cell line cultering conditions

All cell lines, including the generated pinometostat-resistant daughter lines were cultured in RPMI-1640 medium containing GlutaMAX™ supplemented with 10% fetal calf serum, 100 IU/ml Penicillin and Streptomycin, and 0.125µg/ml Amphotericin B (Life Technologies), at 37°C under a 5% $CO_2$ containing atmosphere. Cell lines passed every 3-4 days and routinely tested for the absence of mycoplasma and DNA fingerprinted to assure cell line authenticity.

### Immunoblotting

For the presence of histone modifications, protein was isolated by using the flow-through of RNA isolation using the RNeasy Mini Kit (Qiagen), as described previously.[1] For the expression levels of other proteins, protein was extracted using RIPA buffer supplemented with protease inhibitors (ThermoScienific). Protein extractions were resolved on precast TGX™ gels and transferred to an 0.2 µm nitrocellulose membrane using a Transblot

Turbo Transfer System (Bio-Rad). Blots were then probed with antibodies against H3K79Me2, H3K79Ac, H4K20Ac (cat. nrs. 39143, 39565, 61531, Active Motif), H3K79Me3 (cat.nr. 4260, Cell signaling), H1K25Me3 (cat.nr. 68370, Epigentek), H3K122A, total H3 (cat. nrs. ab33309, ab10799, Abcam), CD133 (PROM1), or GAPDH (cat. nrs. 64326S, 97166S, Cell Signaling). Proteins were visualized using an Odyssey Infrared Imaging System (LI-COR), and protein expression was quantified using the Odyssey software Image Studio Lite ver 4.0.

**RNA sequencing (RNA-seq)**

RNA was isolated from leukemic cells using the RNeasy Mini Kit (Qiagen) and RNA samples were sequenced in quadruplicate on a NextSeq® 500 System (Illumina®). QC was performed with fastQC (http://www.bioinformatics.babraham.ac.uk/projects/ fastqc) and reads were aligned against the human genome assembly (hg19) with STAR.[2] Gene expression levels were quantified using the featureCounts function of the Subread package[3] and read counts were used to identify differential gene expression using DESeq2 (v3.12).[4] Genes were considered differentially expressed between sample groups at a false discovery rate (FDR) adjusted *p*-values of <0.05.

Read counts were used for Gene Set Enrichment Analysis (GSEA) using the GSEA version 4.1.0 software with the Hallmarks gene set database[5]. The heatmap of genes with the most differential GSEA scores was created using the GenePattern software using the Heat Map Image Module[6], and Venn diagrams were created using the interactive Venny tool.[7]

**Chromatin Immunoprecipitation sequencing (ChIP-seq)**

Up to 20 million leukemic cells were crosslinked and lysed using the SimpleChIP kit (Cell Signaling Technology®) according to the manufacturer's recommendations and subsequently sonicated using a Bioruptor sonicator (Diagenode) to generate 150-300 bp fragment size. Next, immunoprecipitation and antibody-protein-DNA precipitation were performed according to the guidelines of the manufacturer.

Antibodies against KMT2A (Bethyl labs; cat.nr. A300-086-A), AFF1 (Abcam; cat.nr. ab31812), H3K4Me3, H3K27Ac and H3K79Me2 (Diagenode; cat. nrs. pAB-003-050, C15410196 and C15410051) were used. ChIP-seq DNA libraries were generated using the NEB Next Ultra DNA library preparation kit for Illumina (New England Biolabs) according to the manufacturer's recommendations and sequenced paired-end on a NextSeq® 500 System (Illumina®). Following QC analysis by fastQC (https://www.bioinformatics.babraham.ac.uk/projects/fastqc/), reads were trimmed using trim_galore (https://www.bioinformatics.babraham.ac.uk/projects/trim_galore/). Reads were then aligned to the human genome, hg19, with bowtie2.[8] Duplicate reads were removed using DeepTools alignmentSieve, with the flag –ignore duplicates.[9] BigWigs were generated using the DeepTools bamCoverage command, with the flags –extendReads –normalize using RPKM, and visualized in the UCSC genome browser.[10] Peaks were called using the Homer tool findPeaks,[11] with the input track provided for background correction, using the –style histone flag. KMT2A::AFF1 peaks were generated from the overlap of KMT2A and AFF1 peaks, after which overlapping peaks closer than 5 kb were stitched together. Heatmaps were generated using DeepTools.

**Assay for Transposase-Accessible Chromatin sequencing (ATAC-seq)**

100.000 SEM cells or $SEM^{PINO\_RES}$ cells were viable sent in duplicate and further processed by Active Motif. Samples were sequenced on a NextSeq® 500 System (Illumina®)) and analyzed as for ChIP-seq, except that duplicate read removal with DeepTools alignmentSieve included the –ATAC shift flag to correct for adapter insertion.

**Flow cytometry (FACS) analysis**

All FACS analysis experiments were performed on a CytoFlex Flow Cytometer (Beckman Coulter). For flow cytometric assays determining the protein expression cells were blocked with Human TruStain FcX™ (BioLegend) and subsequently labeled with ViaKrome 808 (Beckman Coulter) to select for viable cells, as well as with CD133(PROM1)-FITC, CD33-APC (BD Biosciences, cat. nrs. 567029, 551378) or CD85k(LILRB4)-PE (BioLegend, cat. nr. 333008) according to the manufacturer's recommendation. Raw CytoFLEX data were processed using the CytExpert software version2.3 (Beckman Coulter) or FlowJo™ software version7.6.5 (BD Biosiences).

**RNA interference**

Electroporation was performed in 400 µL culture medium without antibiotics containing $4x10^6$/mL cells in 4 mm cuvettes at 350 V for 10 milliseconds using a Gene Pulser Xcell™ Electroporation System (Bio-Rad) in the presence of 10 nM siRNAs directed against *DOT1L*, *HOXA9* (siGENOME SMARTpool Dharmacon™/Horizon),

*KMT2A::AFF1* (named siMA6) targeting the *KMT2A* exon 9–*AFF1* exon 4 *KMT2A::AFF1* fusion site characteristic for SEM cells[12] or AML1-MTG8 fusion gene (named siAGF1), not present in *KMT2A*-rearranged acute leukemias, as non-targeting control.[13]

**Quantitative reverse-transcription PCR analysis**

RNA, isolated using the RNeasy Mini Kit (QIAGEN), was reverse transcribed and the obtained cDNA was used for quantitative reverse-transcription PCR (qRT-PCR) analysis as described previously,[14] and is described in the supplemental methods. The sequences of the used primers were designed to detect the target genes *KM2TA::AFF1* (forward: 5′-ACAGAAAAAAGTGG CTCCCCG-3′; reverse: 5′-TATTGCTGTCAAAGGAGGCGG-3′),[1] *DOT1L* (forward 5′-GGCCCAGATGATTGATGAGA-3′; reverse 5′-CATTTCATCCACTTCCTGAACTC-3′), *HOXA9* (forward 5′-GCGCCTTCTCTGAAAAC-3’; reverse 5′- TGCTCGGTCTTT GTTGA), and the references genes *B2M* (forward 5′-ATGCGGCATCTTCAAA-3′; reverse 5′-GGAGCATTCAGACTTGTCTT-3′), and GUS (forward: 5′-GCGCCGACTT CTCTG-3′; reverse: 5′-CTCCGGCAGGATCAC-3′).

**Mod Spec® mass spectrometry**

The quantification of >80 different histone post-translational modifications (PTMs) by Mod Spec® mass spectrometry was outsourced to Active Motif (Mod Spec® Service: https://www.activemotif.com/catalog/1235/mod-spec). For this, histones were acid extracted, derivatized via propionylation and digested with trypsin. Newly formed N-termini were propionylated as previously described,[15] and measured 3 separate times using the Thermo Scientific TSQ Quantum Ultra mass spectrometer coupled with an

UltiMate 3000 Dionex nano-liquid chromatography system. The data was quantified using Skyline,[16] and represents the percent of each modification within the total pool of that amino acid residue.

**High-throughput drug screening**

For high-throughput drug screening, leukemic cells were semi-automatically seeded in 384-well plates at 10.000 cells/well (Corning) using a MultidropTM dispenser (Thermo Fisher Scientific). Drugs were added using a SciClone ALH3000 liquid handling robot (Caliper Life Sciences) to a final concentration of 1 nM, 10 nM, 100 nM, or 1000 nM. All tested drugs came from commercially available drug libraries, including the Enzo SCREEN-WELL® epigenetics library (BML-2836, 41 compounds; Enzo Life Sciences), the Cayman epigenetics library (11076, 64 compounds; Cayman Chemical), the Sequoia FDA approved anti-neoplastic drug library (165 compounds Sequoia Research Products), the MCE Cell Cycle/DNA Damage Compound Library (HY-L0043; 387 compounds; MedChem Express) and an additional 22 compounds of interest (purchased from Selleckchem). All compounds tested are listed in Supplementary Table 1. Cell viability upon drug exposure was assessed by 4-day thiazolyl blue tetrazolium bromide (MTT; Sigma-Aldrich) assays as previously described,[17] and normalized against DMSO (i.e., no drug) controls. Normalized cell viabilities at the various concentrations of each compound were used to calculate $IC_{50}$ values using GraphPad Prism8, version 8.3.4.

**Cell viability assays**

For the validation of the hits from the high-throughput drug screening as well as evaluation of the chemotherapeutic agents currently used in the treatment of *KMT2A*-rearranged infant ALL cell viability assays were performed using flow cytometry with the 7-AAD viability dye (BioLegend) to discriminate between viable and dead cells. Expanded dose response curves were made using the Tecan D300 Digital Dispenser (Tecan) to dispense venetoclax, prednisolone, dexamethasone, vincristine, daunorubicin, cladribine, cytarabine (all purchased from Selleckchem), and L-asparaginase (Oncospar).

## References


1. Gessner A, Thomas M, Castro PG, et al. Leukemic fusion genes MLL/AF4 and AML1/MTG8 support leukemic self-renewal by controlling expression of the telomerase subunit TERT. *Leukemia.* 2010;24(10):1751-1759.
2. Dobin A, Davis CA, Schlesinger F, et al. STAR: ultrafast universal RNA-seq aligner. *Bioinformatics.* 2013;29(1):15-21.
3. Liao Y, Smyth GK, Shi W. The Subread aligner: fast, accurate and scalable read mapping by seed-and-vote. *Nucleic Acids Res.* 2013;41(10):e108.
4. Love MI, Huber W, Anders S. Moderated estimation of fold change and dispersion for RNA-seq data with DESeq2. *Genome Biol.* 2014;15(12):550.
5. Subramanian A, Tamayo P, Mootha VK, et al. Gene set enrichment analysis: a knowledge-based approach for interpreting genome-wide expression profiles. *Proc Natl Acad Sci U S A.* 2005;102(43):15545-15550.
6. Reich M, Liefeld T, Gould J, Lerner J, Tamayo P, Mesirov JP. GenePattern 2.0. *Nat Genet.* 2006;38(5):500-501.
7. *Venny. An interactive tool for comparing lists with Venn's diagrams.* *https://bioinfogp.cnb.csic.es/tools/venny/index.html* [computer program]. (2007-2015).
8. Langmead B, Trapnell C, Pop M, Salzberg SL. Ultrafast and memory-efficient alignment of short DNA sequences to the human genome. *Genome Biol.* 2009;10(3):R25.
9. Ramirez F, Ryan DP, Gruning B, et al. deepTools2: a next generation web server for deep-sequencing data analysis. *Nucleic Acids Res.* 2016;44(W1):W160-165.
10. Kent WJ, Sugnet CW, Furey TS, et al. The human genome browser at UCSC. *Genome Res.* 2002;12(6):996-1006.
11. Heinz S, Benner C, Spann N, et al. Simple combinations of lineage-determining transcription factors prime cis-regulatory elements required for macrophage and B cell identities. *Mol Cell.* 2010;38(4):576-589.
12. Thomas M, Gessner A, Vornlocher HP, Hadwiger P, Greil J, Heidenreich O. Targeting MLL-AF4 with short interfering RNAs inhibits clonogenicity and engraftment of t(4;11)-positive human leukemic cells. *Blood.* 2005;106(10):3559-3566.

13. Heidenreich O, Krauter J, Riehle H, et al. AML1/MTG8 oncogene suppression by small interfering RNAs supports myeloid differentiation of t(8;21)-positive leukemic cells. *Blood.* 2003;101(8):3157-3163.
14. Spijkers-Hagelstein JA, Pinhancos SS, Schneider P, Pieters R, Stam RW. Chemical genomic screening identifies LY294002 as a modulator of glucocorticoid resistance in MLL-rearranged infant ALL. *Leukemia.* 2014;28(4):761-769.
15. Garcia BA, Mollah S, Ueberheide BM, et al. Chemical derivatization of histones for facilitated analysis by mass spectrometry. *Nat Protoc.* 2007;2(4):933-938.
16. MacLean B, Tomazela DM, Shulman N, et al. Skyline: an open source document editor for creating and analyzing targeted proteomics experiments. *Bioinformatics.* 2010;26(7):966-968.
17. Pieters R, Loonen AH, Huismans DR, et al. In vitro drug sensitivity of cells from children with leukemia using the MTT assay with improved culture conditions. *Blood.* 1990;76(11):2327-2336.

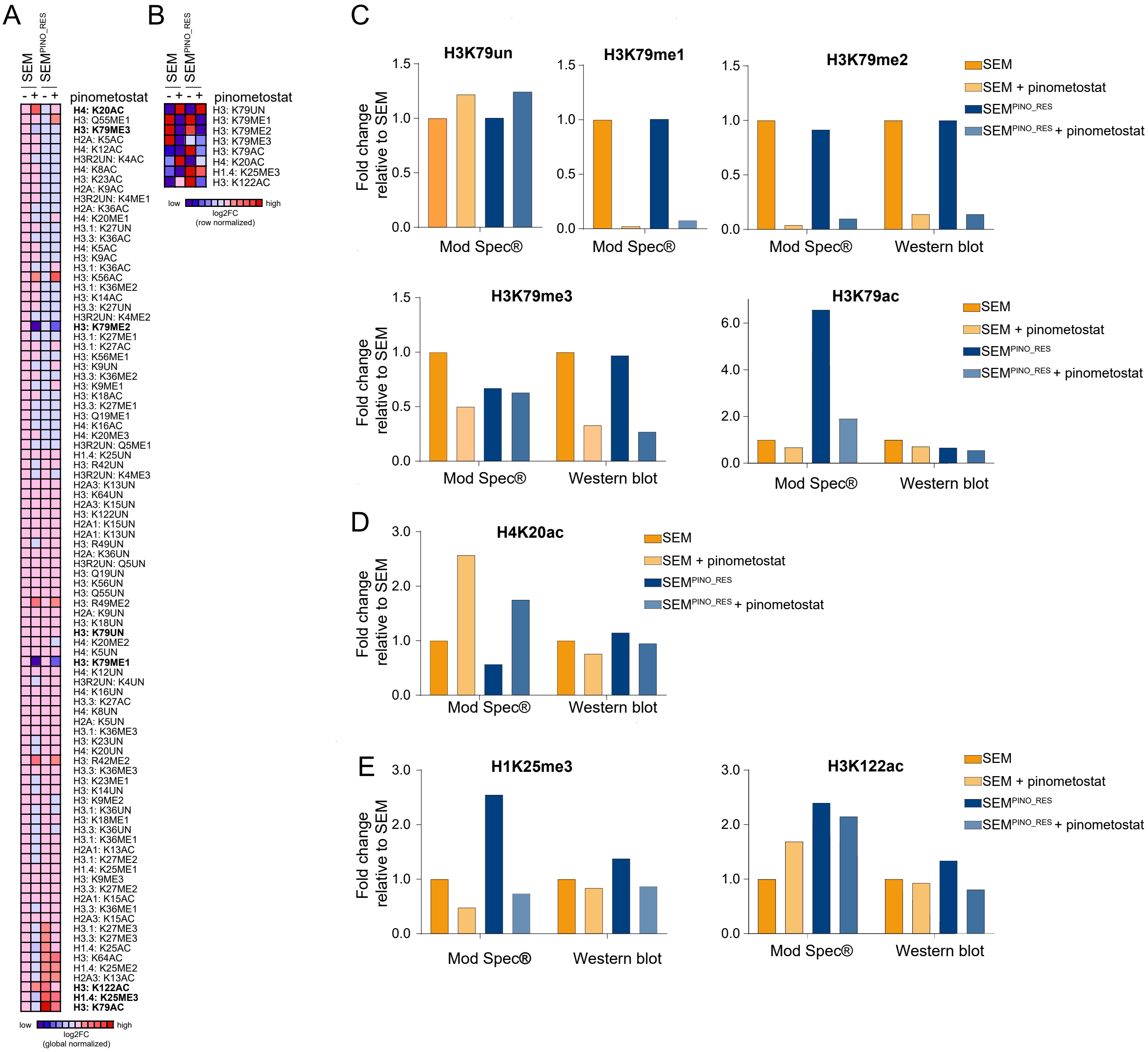


**Figure S1: Global histone modification differences assesement. Related to Figure 1**

Mod Spec®, a mass spectrometry-based measurement for the relative abundance of over 80 distinct histone marks, was performed on SEM and SEM$^{PINO_RES}$ in the presence and absence of pinometostat.

**A**. Heatmap showing the log2 fold change (log2FC) comparisons of histone modification level of SEM + 7 days 50µM pinometostat, SEM$^{PINO_RES}$ or SEM$^{PINO_RES}$ + 7 days 50µM pinometostatin SEM and SEM$^{PINO_RES}$,relative to SEM, based on the percentages of each histone mark modification within the total pool of modifications measured by Mod Spec® at a specific amino acid residue, ranked from lowest to highest log2FC with global normalization. This analysis confirmed no differences in the levels of H3K79 mono-, di-, and tri-methylation (i.e., H3K79me1, H3K79me2, and H3K79me3, respectively) between SEM and SEM$^{PINO_RES}$, and showed equal reduction of these histone marks upon pinometostat exposure in both cell lines. In addition, some histone modifications appeared to be present at differential levels between SEM and SEM$^{PINO_RES}$, including higher levels of histone 4 lysine 20 acetylation (H4K20ac) in SEM, and higher levels of histone 1 lysine 25 tri-methylation (H1K25me3) and histone 3 lysine 122 acetylation (H3K122ac) in SEM$^{PINO_RES}$. **B.** Heatmap showing the log2FC comparisons of all H3K79 histone modifications measured by Mod Spec® of SEM + 7 days 50µM pinometostat, SEM$^{PINO_RES}$ or SEM$^{PINO_RES}$ + 7 days 50µM pinometostatin SEM and SEM$^{PINO_RES}$,relative to SEM, as well as the histone modifications showing the highest log2FC, H4:K20ac, H1K25me3 and H3:K122ac, with row normalization **C.** Fold change of histone modification level of SEM + 7 days 50µM pinometostat, SEM$^{PINO_RES}$ or SEM$^{PINO_RES}$ + 7 days 50µM pinometostat relative to the level in SEM, measured by Mod Spec® or by immunoblot analysis for all H3K79 histone modifications. **D.** fold change of histone modification level of SEM + 7 days 50µM pinometostat, SEM$^{PINO_RES}$ or SEM$^{PINO_RES}$ + 7 days 50µM pinometostat compared to the level in SEM, measured by mass spec or by immunoblot analysis for histone modification most reduced in SEM$^{PINO_RES}$ H4K20ac. **E** fold change of histone modification level of SEM + 7 days 50µM pinometostat, SEM$^{PINO_RES}$ or SEM$^{PINO_RES}$ + 7 days 50µM pinometostat compared to the level in SEM, measured by mass spec or by immunoblot analysis for the histone modifications most enhanced in SEM$^{PINO_RES}$, H1K25me3 and H3K122ac. This reveals that the differences found for H4:K20ac, H1K25me3 and H3:K122ac could not be validated by immunoblot analyses. Moreover, these data demonstrated that the global landscape of histone modifications between SEM cells and SEM$^{PINO_RES}$ largely remained similar. The only histone modification that is downregulated in response to pinometostat exposure appeared to be H3K79 methylation, demonstrating the specificity of this agent.

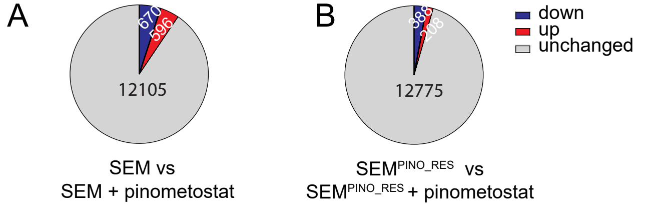


**Figure S2: Gene expression comparisons of SEM and SEM$^{PINO_RES}$ treated with pinometostat compared to untreated cells. Related to Figure 2**

**A-B.** Pie charts showing the number of genes that are significantly downregulated (blue), upregulated (red), or remain unchanged (gray) between **A** SEM and SEM treated for 7 days with 50 uM pinometostat and **B** between SEM and SEM$^{PINO_RES}$ treated for 7 days with 50 uM pinometostat, 4 biological replicates each.

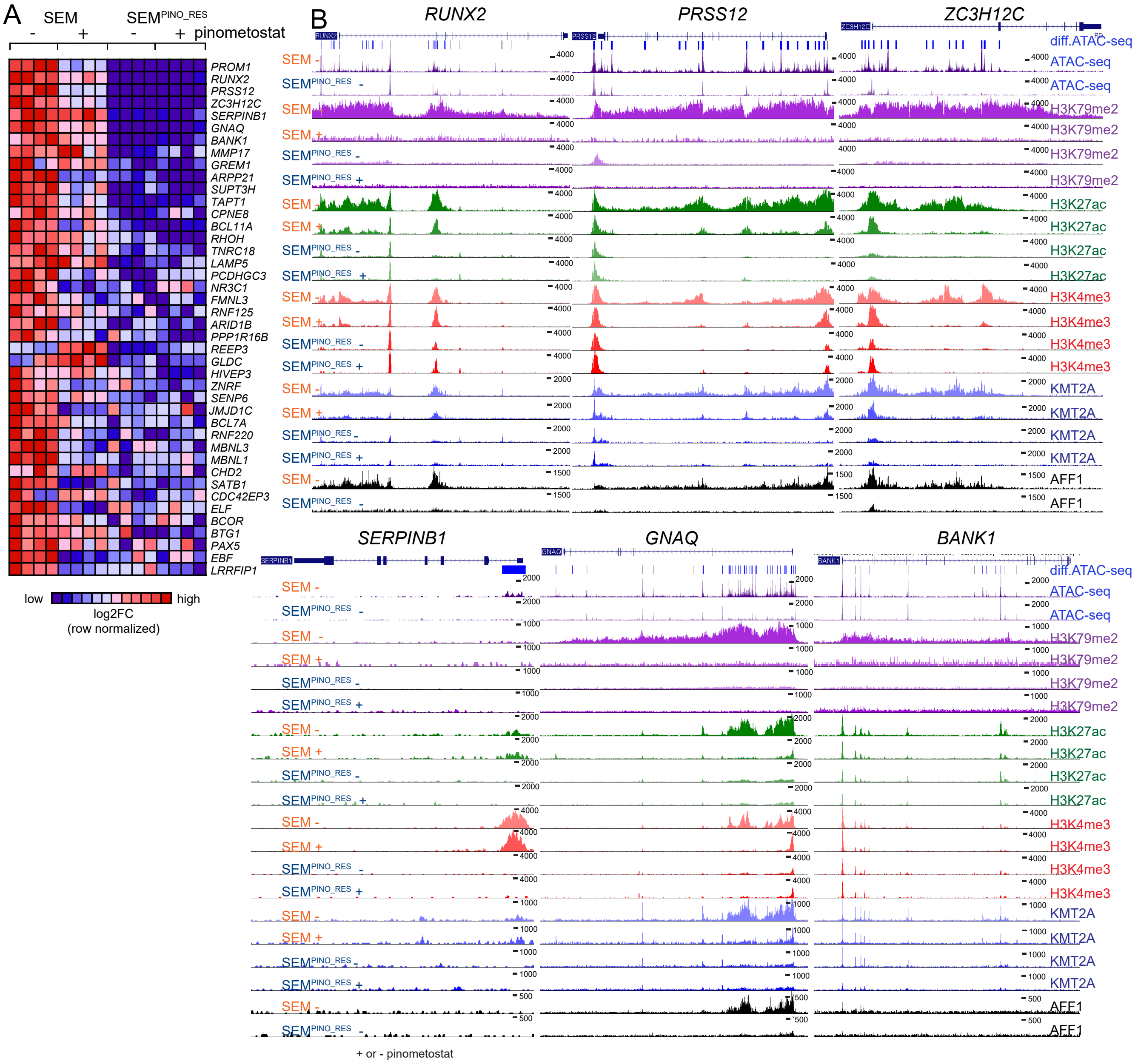


**Figure S3: RNA expression in SEM$^{PINO_RES}$ of genes previously associated with KMT2A-rearranged leukemias and/or high levels of H3K79Me2 reduced in SEM$^{PINO_RES}$. Related to Figure 3**

**A** Heatmap of RNA expression of putative KMT2A fusion target genes downregulated in SEM$^{PINO_RES}$ compared to SEM cells. Normalized counts of RNAseq shown of SEM and SEM$^{PINO_RES}$, both untreated or teated 7 days with 50µM pinometostat, 4 biological replicates each, mean with standard deviation (SD) .

**B** ATACseq differences between untreated SEM and SEM$^{PINO_RES}$ cells at *PRSS12*, *ZCH12C*, *SERPINB1*, *GNAQ* and *BANK1*. Blue lines indicate significant decrease of chromatin accessibility in SEM$^{PINO_RES}$ cells compared to SEM, grey lines indicate equal chromatin accessibility in both cell lines. 2 biological replicates; ChIPseq tracks showing H3K79me2, H3K27ac, H3K4me3, KMT2A in SEM and SEM$^{PINO_RES}$ cells after 7 days treatment + or - 50µM pinometostat and AFF1 in untreated SEM and SEM$^{PINO_RES}$ cells at genes *PRSS12*, *ZCH12C*, *SERPINB1*, *GNAQ* and *BANK1*

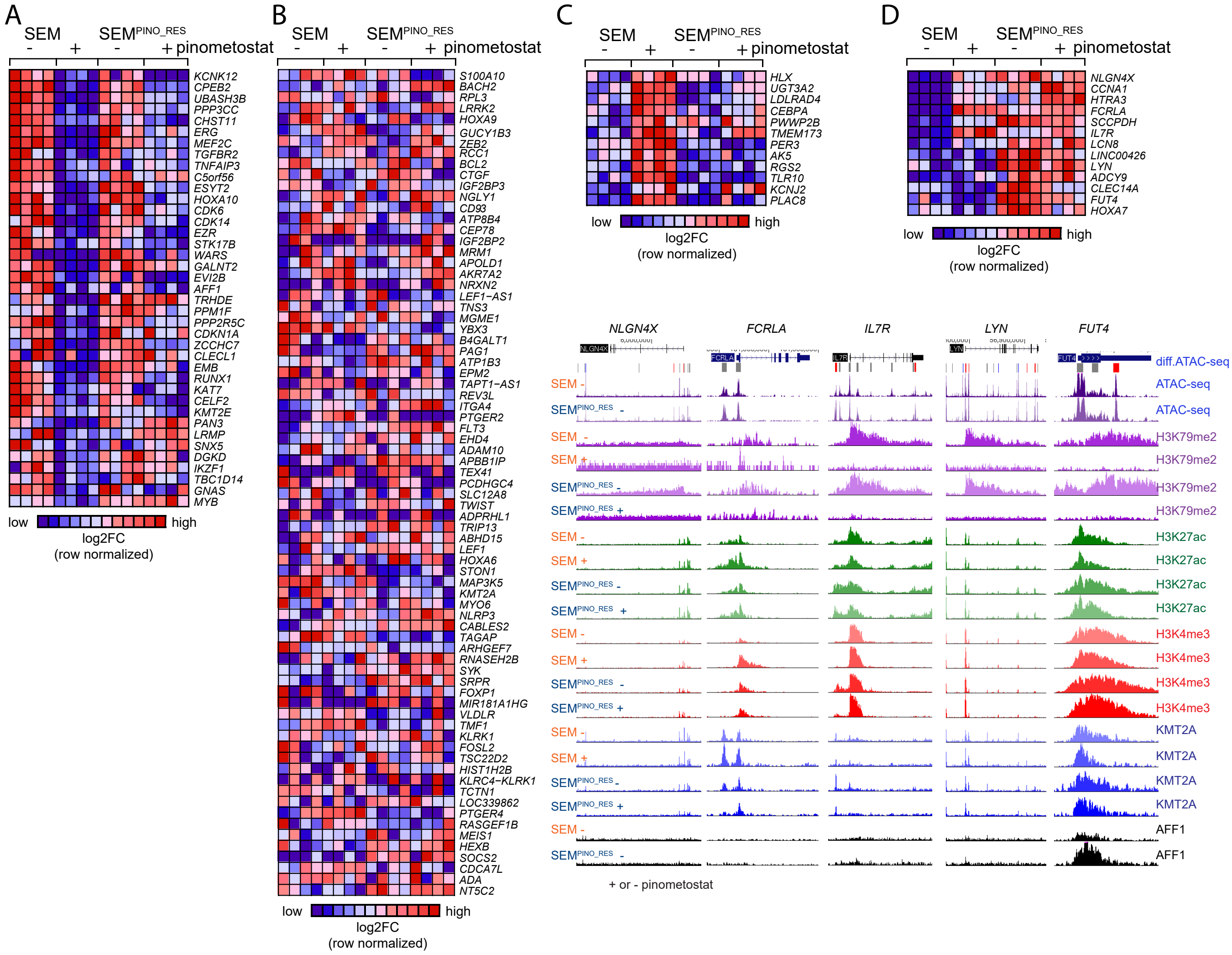


**Figure S4: Putative KMT2A fusion target genes with no transcriptional changes or upregulated in SEM$^{PINO_RES}$ compared to SEM. Related to Figure 4**

**A-D** Normalized counts of RNAseq shown of SEM and SEM$^{PINO_RES}$, both untreated or teated 7 days with 50μM pinometostat, 4 biological replicates each.

**A.** Heatmap of RNA expression of putative KMT2A fusion target genes with no transcriptional changes between SEM and SEM$^{PINO_RES}$, yet downregulated in SEM treated with pinometostat compared to untreated SEM cells. **B.** Heatmap of RNA expression of putative KMT2A fusion target genes with no transcriptional changes between SEM and SEM$^{PINO_RES}$ and no differences in SEM treated with pinometostat compared to untreated SEM cells. **C.** Heatmap of RNA expression of putative KMT2A fusion target genes with no transcriptional changes between SEM and SEM$^{PINO_RES}$, yet upregulated in SEM treated with pinometostat compared to untreated SEM cells. **D.** Heatmap of RNA expression of putative KMT2A fusion target genes upregulated in SEM$^{PINO_RES}$ compared to SEM cells.

**E.** ATACseq and ChIPseq tracks of SEM and SEM$^{PINO_RES}$ cells for the putative KMT2A fusion target genes significantly upregulated in SEM$^{PINO_RES}$ compared to SEM cells. Blue lines of the ATACseq indicate significant more open chromatin acces in SEM compared to SEM$^{PINO_RES}$ cells. 2 biological replicates; ChIPseq tracks show H3K79me2, H3K27ac, H3K4me3, KMT2A in SEM and SEM$^{PINO_RES}$ cells after 7 days treatment + or - 50μM pinometostat and AFF1 in untreated SEM and SEM$^{PINO_RES}$ cells at the putative KMT2A fusion target genes significantly upregulated in SEM$^{PINO_RES}$ compared to SEM cells .

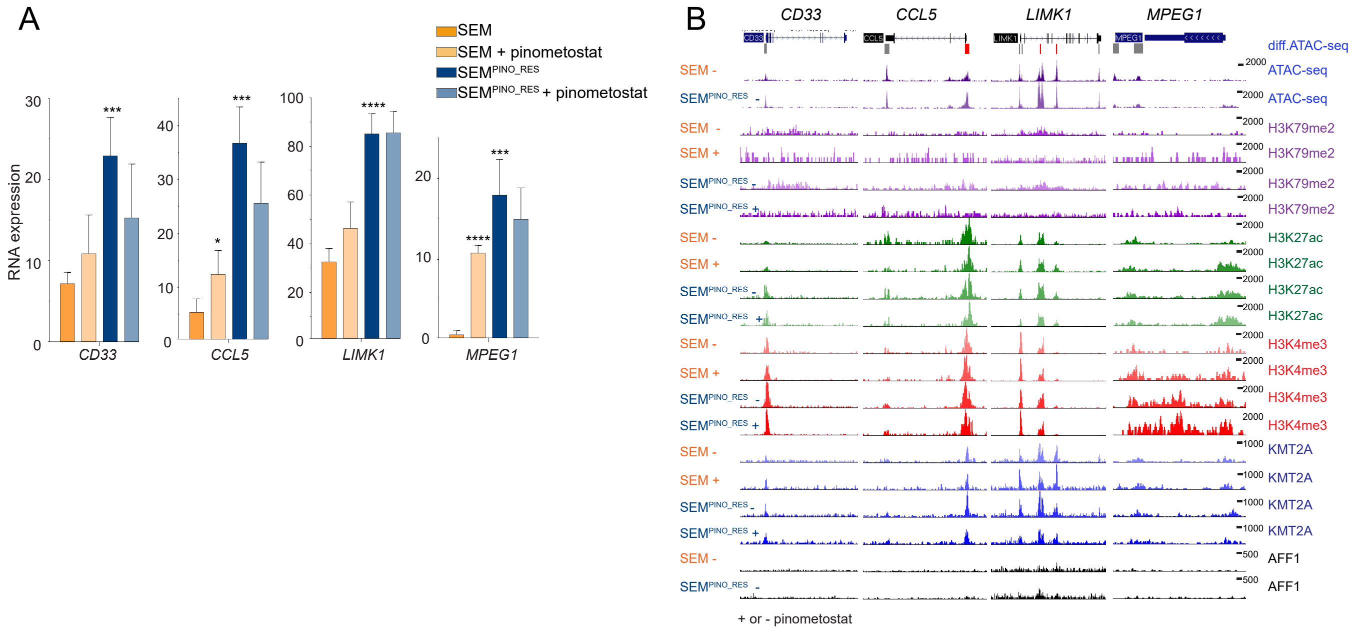


**Figure S5: Myeloid associated genes upregulated in SEM$^{PINO_RES}$ compared to SEM. Related to Figure 5**

**A** *CD33*, *CCL5*, *LIMK1* and *MPEG1* RNA expression of SEM and SEM$^{PINO_RES}$ after 7 days treatment + or - 50µM pinometostat. Mean of normalized counts of RNAseq data with SD depicted, 4 biological replicates each. *p* values calculated with unpaired t-test, * *p*<0.05, * *p*<0.05, ** *p*<0.005, *** *p*<0.0005, **** *p*<0.0001.
**B** ATACseq differences between untreated SEM and SEM$^{PINO_RES}$ cells at *CD33*, *CCL5*, *LIMK1* and *MPEG1*. Red lines indicate significant increase of chromatin accessibility in SEM$^{PINO_RES}$ cells compared to SEM, grey lines indicate equal chromatin accessibility in both cell lines. 2 biological replicates; ChIPseq tracks showing H3K79me2, H3K27ac, H3K4me3, KMT2A in SEM and SEM$^{PINO_RES}$ cells after 7 days treatment + or - 50µM pinometostat and AFF1 in untreated SEM and SEM$^{PINO_RES}$ cells at genes *CD33*, *CCL5*, *LIMK1* and *MPEG1*.

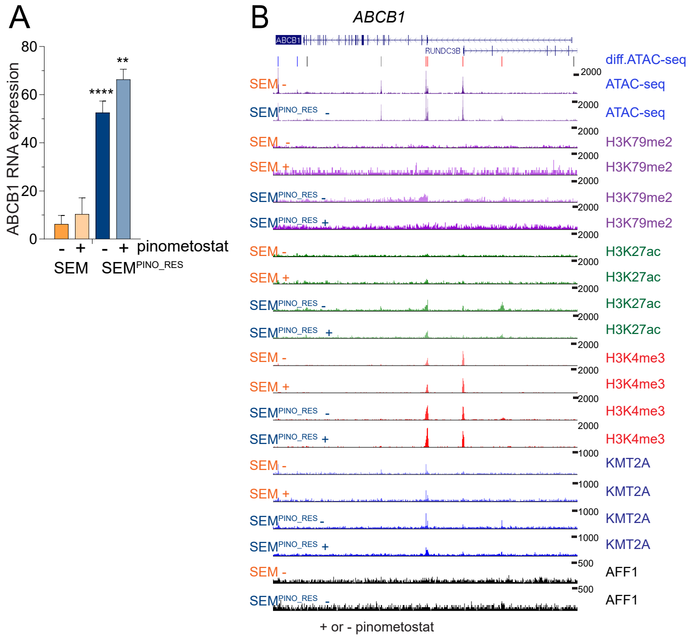


**Figure S6: Validation of sensitizing hits nimustine, carmustine and BIX01294 reveals cell line specificity. Related to Figure 6**

**A.** *ABCB1* expression of SEM and SEM$^{PINO_RES}$ after 7 days treatment + or - 50µM pinometostat. Mean of normalized counts of RNAseq data with SD depicted, 4 biological replicates each. *p* values calculated with unpaired t-test, * *p*<0.05, * *p*<0.05, ** *p*<0.005, *** *p*<0.0005, **** *p*<0.0001. **B.** ATACseq differences between untreated SEM and SEM$^{PINO_RES}$ cells at *ABCB1*. Red lines indicate significant increase of chromatin accessibility in SEM$^{PINO_RES}$ cells compared to SEM, grey lines indicate equal chromatin accessibility in both cell lines. 2 biological replicates; ChIPseq tracks showing H3K79me2, H3K27ac, H3K4me3, KMT2A in SEM and SEM$^{PINO_RES}$ cells after 7 days treatment + or - 50µM pinometostat and AFF1 in untreated SEM and SEM$^{PINO_RES}$ cells at genes *ABCB1*.

# Supplementary Table S1: List of genes Venn diagramms

| Figure 2B | | Figure 2C | | Figure 2D | | Figure 2D | | Figure 2D | | Figure 2D | | Figure S2A | | Figure S2B | |
|---|---|---|---|---|---|---|---|---|---|---|---|---|---|---|---|
| All RNAseq data | | KMT2A fusion target genes | | KMT2A::AFF1 driven gene expression Guenther *et al*. | | Top 50 genes H3K79me2 ChIPseq Krivtsov *et al*. | | siKMT2A:AFF1/ENL knockdown van der Linden *et al*. | | spreading genes KMT2A::AFF1 patients Kerry *et al*. | | All RNAseq data | | All RNAseq data | |
| $SEM^{PINO\_RES}$ compared to SEM | | $SEM^{PINO\_RES}$ compared to SEM | | $SEM^{PINO\_RES}$ compared to SEM | | $SEM^{PINO\_RES}$ compared to SEM | | $SEM^{PINO\_RES}$ compared to SEM | | $SEM^{PINO\_RES}$ compared to SEM | | SEM + pinometostat compared to SEM maternal | | $SEM^{PINO\_RES}$ + pinometostat compared to $SEM^{PINO\_RES}$ | |
| up | down | up | down | up | down | up | down | up | down | up | down | up | down | up | down |
| A2M | ABR | ADCY9 | ARID1B | HOXA7 | PRSS12 | ADCY9 | PROM1 | FCRLA | SATB1 | IL7R | GNAQ | ABCB10 | ABHD12 | ABCB10 | SEPTIN6 |
| AARS | ACAP2 | CCNA1 | ARPP21 | | PROM1 | CCNA1 | CDC42EP3 | HOXA7 | GNAQ | LINC00426 | RHOBTB1 | ABCG2 | ABHD4 | ABCD3 | MARCHF8 |
| AASS | ACBD3 | CLEC14A | BANK1 | | TAPT1 | CLEC14A | GNAQ | NLGN4X | ARID1B | LYN | TRIB2 | ABLIM1 | ABRACL | S100A6 | ABCA2 |
| ABCB1 | ACSS2 | FCRLA | BCL11A | | JMJD1C | FUT4 | RNF220 | | SENP6 | | RHOBTB3 | ABTB2 | ACAP2 | ADAM22 | ABRACL |
| ABCF2 | ACTN1 | FUT4 | BCL7A | | GNAQ | HOXA7 | LAMP5 | | MBNL3 | | PTCH1 | ACER3 | ACBD3 | ADGRL2 | ACKR4 |
| ACAT1 | ACVR2B | HOXA7 | BCOR | | EBF1 | HTRA3 | GREM1 | | HIVEP3 | | ATRNL1 | ACVR1C | ACSL1 | AHDC1 | ADAM17 |
| ACKR4 | ACY3 | HTRA3 | BTG1 | | BCL7A | LCN8 | ZNRF1 | | REEP3 | | PARP15 | ADAM22 | ACTN1 | AHSA1 | ADGRE5 |
| ACTR3B | ADAM19 | IL7R | CDC42EP3 | | ARID1B | SCCPDH | RUNX2 | | GLDC | | MARCKS | ADAM23 | ACTN4 | ANKRD13B | ADK |
| ADAM15 | ADARB1 | LCN8 | CHD2 | | RNF220 | | PCDHGC3 | | | | ZDBF2 | ADAM28 | ACTR3 | APAF1 | AES |
| ADAM23 | ADGRL1 | LINC00426 | CPNE8 | | SENP6 | | SERPINB1 | | | | LCK | ADAMTS1 | ACVR1B | ARAP1 | AK4 |
| ADAP2 | ADGRL3 | LYN | EBF1 | | LAMP5 | | MMP17 | | | | VAT1L | ADAP2 | ADAM17 | ARHGEF18 | ALDH3B1 |
| ADCY9 | ADNP2 | NLGN4X | ELF1 | | TNRC18 | | | | | | ZNF292 | ADGRL2 | ADGRE5 | ARPC1A | AMDHD2 |
| AEBP1 | ADPRH | SCCPDH | FMNL3 | | ZC3H12C | | | | | | ARRDC3 | ADI1 | ADGRL3 | ATAD2 | ANGPT1 |
| AHCY | AFF4 | | GLDC | | RUNX2 | | | | | | AFF4 | AEBP1 | ADK | ATP6V1B2 | ANKRD28 |
| AHCYL2 | AGAP1 | | GNAQ | | REEP3 | | | | | | NDRG1 | AFF3 | ADNP2 | ATP6V1D | AP5B1 |
| AIM1 | AGGF1 | | GREM1 | | SUPT3H | | | | | | ZC4H2 | AHCYL2 | ADRBK1 | BACH2 | APP |
| AKR1B1 | AGO4 | | HIVEP3 | | CPNE8 | | | | | | LNPEP | AHDC1 | AES | SCN9A | ARFIP2 |
| ANGPT1 | AGPAT3 | | JMJD1C | | RHOH | | | | | | REEP3 | AHRR | AFF1 | BBS10 | ARHGDIB |
| ANKH | AHCYL1 | | LAMP5 | | | | | | | | ATP6V1A | AK5 | AFF4 | BCR | ARHGEF6 |
| ANO2 | AKNA | | LRRFIP1 | | | | | | | | ALDOA | ALCAM | AGO2 | BLM | ARID5A |
| AP1S1 | ALDH7A1 | | MBNL1 | | | | | | | | PGM1 | ALDH3A2 | AGTPBP1 | BRCA1 | ARRDC4 |
| APOA1BP | ALDOA | | MBNL3 | | | | | | | | CXXC5 | ANKRD44 | AKAP13 | BRWD3 | ASF1A |
| APOL3 | ALDOC | | MMP17 | | | | | | | | PHKB | ANKRD52 | AKNA | BUB1 | ATF5 |
| ARF5 | ALOX5AP | | NR3C1 | | | | | | | | CCDC50 | API5 | AKR1A1 | C2CD5 | ATG101 |
| ARID3C | AMMECR1L | | PAX5 | | | | | | | | NFYA | ARAP1 | ALDOC | C2orf44 | ATP8A1 |
| ARID5B | AMOT | | PCDHGC3 | | | | | | | | BTG1 | ARHGEF18 | ALG5 | CACNA2D1 | ATRN |
| ARIH2 | AMOTL1 | | PPP1R16B | | | | | | | | CD47 | ARHGEF40 | ALMS1-IT1 | CADPS2 | B4GALT2 |
| ARMCX2 | ANKRD18DP | | PROM1 | | | | | | | | | ARPC1A | ALOX5AP | CCDC14 | BCAS4 |
| ARPC1A | ANTXR1 | | PRSS12 | | | | | | | | | EPHA3 | AMFR | CCNA1 | BCAT1 |
| ARPC1B | APBA1 | | REEP3 | | | | | | | | | ASUN | ANK2 | CCND3 | BID |
| ASAH1 | ARAP2 | | RHOH | | | | | | | | | ATP6V0A1 | ANKRD18D | CCNE2 | BSG |
| ATG101 | ARHGAP19 | | RNF125 | | | | | | | | | ATP6V0E2- | ANKRD28 | CCNF | BZRAP1-AS |
| ATG16L2 | ARHGEF1 | | RNF220 | | | | | | | | | B4GALT6 | ANXA11 | CDAN1 | C12orf77 |
| ATP5J2 | ARID1B | | RUNX2 | | | | | | | | | ROR1 | ANXA2 | CDC5L | C16orf13 |
| ATP6V1F | ARL6IP5 | | SATB1 | | | | | | | | | BBS2 | ANXA2P2 | CDK1 | C19orf70 |
| AXIN1 | ARPP21 | | SENP6 | | | | | | | | | BCL6 | ANXA2R | VCAN | C8orf82 |
| B2M | ARRB1 | | SERPINB1 | | | | | | | | | BCORL1 | APOBEC3C | CELF1 | CA5B |
| B4GALT2 | ARRB2 | | SUPT3H | | | | | | | | | BMF | APOBEC3D | CENPE | CACNA2D4 |
| BAHCC1 | ARRDC3 | | TAPT1 | | | | | | | | | SLC16A3 | ARHGAP19 | CENPI | CAMK2D |
| BAX | ASCC3 | | TNRC18 | | | | | | | | | BRCA1 | ARHGAP21 | CGN | CAMKK1 |
| BAZ1B | ATAD2B | | ZC3H12C | | | | | | | | | BRF1 | ARHGAP30 | CHORDC1 | CCDC162P |
| BCAP29 | ATF2 | | ZNRF1 | | | | | | | | | BRWD3 | ARHGDIA | CLASP2 | CCDC85B |
| BCL6 | ATF3 | | | | | | | | | | | EYA1 | ARHGDIB | TRIM9 | CCNB1IP1 |
| BLK | ATP10A | | | | | | | | | | | BUB1 | ARHGEF6 | PABPC4L | CD151 |
| BLNK | ATP13A2 | | | | | | | | | | | BUB1B | ARID1B | CLTCL1 | CD248 |
| BRD7 | ATP2A3 | | | | | | | | | | | BVES | ARPC2 | CNTRL | CD44 |
| BUD31 | ATP2B4 | | | | | | | | | | | C11orf54 | ARPC5L | CPED1 | CD52 |
| BZRAP1-AS1 | ATP6V1A | | | | | | | | | | | C12orf29 | ARPP21 | ALOX5AP | CD58 |
| C12orf75 | ATP7A | | | | | | | | | | | C14orf37 | ARRDC3 | CRYBG3 | CDK14 |
| C17orf51 | ATPAF1 | | | | | | | | | | | C17orf96 | ASF1A | CERK | CDK6 |
| C17orf96 | ATRNL1 | | | | | | | | | | | C18orf54 | ASNS | CSNK1G1 | CDKN2A |
| C19orf70 | B3GNT2 | | | | | | | | | | | C2CD5 | ATF3 | ANTXR1 | CELF2 |
| C2orf81 | BACH1 | | | | | | | | | | | C2orf81 | ATF5 | CXCR4 | CFAP97 |
| C4orf32 | BAIAP2L1 | | | | | | | | | | | C7orf60 | ATF7IP | DACT1 | CHST11 |
| C7orf43 | BANK1 | | | | | | | | | | | C9orf64 | ATG101 | DDIAS | CIRBP |
| C7orf49 | BBS1 | | | | | | | | | | | CA2 | ATP10A | DNA2 | CLEC11A |
| C7orf73 | BCL11A | | | | | | | | | | | CADPS2 | ATP5S | DNAH10 | CLEC14A |
| C8orf31 | BCL7A | | | | | | | | | | | CALR | ATP8A1 | DNAJA1 | FSCN1 |
| C8orf33 | BCOR | | | | | | | | | | | CAPSL | BACH1 | DNMT1 | CLIC1 |
| C9orf114 | BDH1 | | | | | | | | | | | CASC5 | BANK1 | DSCC1 | CLIC4 |
| CACNB1 | BEND5 | | | | | | | | | | | TNS1 | BCAS4 | DYNLL1 | CNBP |
| CALR | BEX4 | | | | | | | | | | | IKZF3 | BCAT1 | EDRF1 | CPEB2 |
| CALU | BHLHE41 | | | | | | | | | | | CAV1 | BCL11A | EID2B | ARPP21 |
| CAPSL | BIN1 | | | | | | | | | | | CBFA2T3 | BCL7A | EML6 | CPXM1 |
| CAPZA2 | BIN2 | | | | | | | | | | | CBX1 | BCL7B | ESCO2 | CRACR2A |
| CASC4 | BIRC6 | | | | | | | | | | | CBX5 | BEX4 | ETNK1 | CRIM1 |
| CCDC124 | BMP2K | | | | | | | | | | | CBX6 | BID | AGGF1 | CRTC3 |
| CCDC146 | BMPR1A | | | | | | | | | | | CCAR1 | BIN1 | FAM111A | CSF2RB |
| CCDC155 | BMS1P20 | | | | | | | | | | | CCDC102A | BIN2 | FAM129C | CST7 |
| CCDC25 | BNIP3 | | | | | | | | | | | CCDC110 | BMP2K | FANCA | CTBP2 |
| CCDC26 | BNIP3L | | | | | | | | | | | CCDC15 | BMPR1A | FANCD2 | CTNNBL1 |
| CCDC81 | BOLA1 | | | | | | | | | | | CCDC81 | BNIP1 | FANCI | CTSO |
| CCDC86 | BRWD1 | | | | | | | | | | | CCL5 | BNIP2 | ATAD2B | CUTC |
| CCL5 | BTBD11 | | | | | | | | | | | CCNA1 | BRPF1 | FBN2 | CYFIP2 |
| CCNA1 | BTBD3 | | | | | | | | | | | SORBS1 | BTF3 | FBXO9 | DALRD3 |
| CCT6A | BTG1 | | | | | | | | | | | CD180 | BTG3 | FERMT2 | DAZAP2 |
| CD180 | C11orf65 | | | | | | | | | | | CD24 | C10orf12 | KLHL14 | DCAF4 |
| CD33 | C12orf57 | | | | | | | | | | | CD86 | C10orf54 | G3BP1 | DDX5 |
| CD3EAP | C14orf28 | | | | | | | | | | | CDAN1 | C12orf77 | GALNT3 | DEAF1 |
| CD52 | C16orf54 | | | | | | | | | | | CDC20 | C16orf54 | GBP3 | DFNA5 |
| CD68 | C1orf115 | | | | | | | | | | | CDH11 | C20orf196 | GCSAM | DIAPH1 |
| CD70 | C21orf91 | | | | | | | | | | | CDKL3 | C4orf46 | GNG11 | DLGAP4 |
| CD79A | C3orf38 | | | | | | | | | | | CDON | C5orf56 | GPATCH11 | DNASE2 |
| CD79B | C4orf46 | | | | | | | | | | | CDX2 | C9orf16 | GSDMB | DNM1 |
| CDHR3 | C5orf63 | | | | | | | | | | | CEBPA | CA11 | GXYLT1 | DNPH1 |
| CDK5 | C6orf223 | | | | | | | | | | | CENPE | CA5B | HNRNPH1 | CACNB4 |
| CEP164 | CACHD1 | | | | | | | | | | | CENPF | CACNB4 | HNRNPH3 | EDARADD |

| | | | | | |
|---|---|---|---|---|---|
| CEP19 | CACNB4 | CEP164 | CALM1 | HPGD | EDEM1 |
| CEP41 | CALM1 | CEP19 | CAMK1D | HSPA4L | EEF1B2 |
| CHCHD2 | CAMK1D | CGN | CAMK2D | HSPA8 | EEF1G |
| CHP1 | CAND1 | CHORDC1 | CAP1 | HSPH1 | EEF2KMT |
| CHPF2 | CAPG | CHST2 | CAPG | HTRA3 | EGLN1 |
| CHST6 | CASKIN2 | CIDEB | CARS | IDO1 | EIF3E |
| CLASRP | CASP8AP2 | CKAP4 | CASC15 | IER3 | EIF3L |
| CLCF1 | CBLB | CKAP5 | CBS | IGFBP3 | EMP3 |
| CLCN7 | CCDC107 | CLASP2 | CBX4 | IKBKAP | EPB41L4A-A |
| CLDN12 | CCDC117 | CLMN | CCDC50 | INTS2 | ERCC1 |
| CLDN15 | CCDC50 | CLU | CCDC69 | INTS8 | ERGIC3 |
| CLEC14A | CCDC69 | CMAHP | CCL28 | ISCU | ESYT2 |
| CLN6 | CCDC85C | CNOT11 | CCNB1IP1 | ITGAL | EVI2B |
| CLPP | CCL28 | COQ9 | CCZ1B | ITPKB | EVL |
| COLGALT1 | CCNG2 | CPED1 | CD109 | ITSN1 | EZR |
| COPS5 | CCP110 | CPNE1 | CD151 | KANK2 | FAM107B |
| COPS6 | CD109 | CRLS1 | CD164 | KCNJ2 | FAM117A |
| COX17 | CD164 | CROCC | CD19 | MARCKS | LINC01108 |
| COX6C | CD19 | CROCCP2 | CD248 | KIAA0125 | FAM60A |
| CPED1 | CD37 | CRYBG3 | CD44 | KIAA0922 | FAM65B |
| CPNE7 | CD47 | CERK | CDK14 | KIAA1524 | FBRSL1 |
| CPPED1 | CD59 | CSNK1G1 | CDK17 | KIF21A | FBXL5 |
| CPTP | CD72 | CSRP2 | CDK6 | LAIR1 | FBXO31 |
| CRCP | CDC42EP3 | ANTXR1 | CDKN1A | LBR | FCGRT |
| CSF2RB | CDK17 | CTR9 | CDKN1B | LRRCC1 | FCMR |
| CSTB | CDK2AP1 | LSP1 | CDKN2A | LRRK2 | FKBP10 |
| CTNNBL1 | CDKN1B | CTTN | CDKN2D | LYZ | FKBP8 |
| CTSA | CDKN2C | CXCR4 | CEBPB | MAP2 | FLJ23867 |
| CTTNBP2 | CENPV | CXorf21 | CEBPG | MARK1 | FNBP1 |
| CUEDC2 | CEP170 | CXXC1 | CELF2 | DBT | SNX30 |
| CUL1 | CEPT1 | CXXC4 | CENPU | MDM2 | FRMD4B |
| CYB5R3 | CERK | CYB561A3 | CENPV | MECOM | FUBP3 |
| CYCS | CERS4 | CYFIP1 | CHAC1 | MED21 | FUS |
| CYP51A1 | CHD2 | DAAM1 | CHI3L2 | MLLT4 | FUT4 |
| DBF4 | CHD3 | DACT1 | CHN2 | MTCH2 | GAA |
| DCAF13 | CHD6 | DCHS1 | CHST11 | MTIF3 | GAB1 |
| DGCR14 | CHD9 | DDX23 | CHSY1 | MYBPC2 | GADD45B |
| DHRS9 | CHN2 | DDX52 | CIRBP | NBPF1 | GAS5 |
| DHX33 | CHST15 | DDX58 | CLEC14A | NCAPD2 | GCAT |
| DLEU7-AS1 | CIITA | DEPDC1B | CLEC2B | NCAPD3 | GLG1 |
| DLGAP4 | CKAP2 | DHRS3 | CLEC9A | CLCN3 | GLTSCR2 |
| DNAJA1 | CLCN3 | DLL4 | CLECL1 | NPEPPS | GNAI1 |
| DNAJC11 | CLCN5 | DMD | CLIC1 | PAN2 | GNB2L1 |
| DNAJC2 | CLEC2B | DNAJA1 | CLIC4 | NEK7 | GOLIM4 |
| DNAJC8 | CLEC9A | DNAJC18 | CLPX | SOS1 | GOLM1 |
| DNTT | CLN5 | DPY19L2P2 | CLTC | PEX6 | ALDOC |
| DOK3 | CMTM3 | DPY19L3 | CNBP | PHTF1 | GSTZ1 |
| DOK4 | CMTM4 | DPY19L4 | CNDP2 | PIGN | GUCD1 |
| DST | CPNE3 | DPYD | COMT | DCP2 | H3F3B |
| DTNA | CPNE8 | DTNB | COX7A2L | KLHL9 | DBN1 |
| DUS1L | CREB3L4 | DTX4 | CPEB2 | PLEKHA2 | HEXA |
| DYSF | CREBZF | DUSP7 | CPNE8 | PLS1 | SERINC5 |
| E2F4 | CREG1 | DYNLL1 | CRACR2A | POLA2 | HIVEP2 |
| EFHD2 | CRKL | EDEM3 | CRMP1 | POLQ | HLA-A |
| EFNB2 | CRMP1 | EDIL3 | CSPG4 | POP1 | HLA-B |
| EGFL7 | CSAD | EFCAB2 | CSRNP1 | PPID | HLA-C |
| EIF5AL1 | CSGALNACT2 | EGLN3 | CTBP2 | PPIL2 | HLA-E |
| EIF5B | CSNK1G3 | EHD1 | CTDSPL2 | PPM1E | HNRNPDL |
| ELP6 | CSPG4 | EHMT2 | CTNNA1 | PPP2R1B | HOXA10 |
| ENDOD1 | CSRNP1 | EIF2B4 | CTSC | PRKG1 | HPDL |
| ERGIC1 | CTDSP2 | ELMOD2 | CUX1 | LRP5 | HS6ST2 |
| ERICH1 | CTDSPL | ENDOD1 | CYFIP2 | RAD54B | BIN1 |
| ESRRA | CTDSPL2 | ENHO | CYLD | RAD54L | ICAM3 |
| ETFB | CTNNA1 | EPHA7 | CYTH1 | RAG1 | IDS |
| F2RL3 | CTSC | EPHB3 | CYTIP | RBM5 | IFITM2 |
| FAM129C | CTXN1 | NELL1 | DAD1 | EIF4A2 | MED12L |
| FAM200A | CWF19L1 | EPHX1 | DALRD3 | SCD5 | IGFBP7 |
| FAM46C | CXXC5 | ESCO2 | DAZAP2 | RPAIN | IGFLR1 |
| FAM73B | CYP4V2 | ETNK1 | DBN1 | RPE | IL2RG |
| FBXL6 | CYTH1 | EXOC5 | DCK | RSRP1 | IRAK1 |
| FCRLA | CYTIP | EYA3 | DDAH2 | FAM111B | ISG20 |
| FERMT1 | DAB2IP | FAM120A | DDIT3 | SAMD9 | ITGA6 |
| FIS1 | DARS | PLOD2 | DDN | SDC2 | KLF6 |
| FJX1 | DBN1 | FAM129C | DDX19B | REEP3 | SERINC2 |
| FOXRED1 | DBT | FAM63A | DDX5 | SKP2 | ITM2B |
| FOXRED2 | DCHS2 | FAM81A | DDX6 | SLC27A4 | JCHAIN |
| FSTL5 | DCK | BNIP3 | DEAF1 | SLC8A1 | JUND |
| FUS | DCP2 | FANCA | DENND3 | SMC2 | KCNG1 |
| FUT4 | DCTN5 | FANCD2 | DESI2 | SNAPC3 | KCNK12 |
| FUT7 | DCUN1D3 | FANCG | DFNA5 | SNX7 | KCNMB1 |
| FXYD5 | DDAH2 | FBN2 | DGKD | SORL1 | DPYSL3 |
| G6PC3 | DDN | FBXL7 | DGKH | GSTCD | SLAMF1 |
| GAB1 | DGKH | FBXO42 | DHTKD1 | SPIB | KLF10 |
| GABRA4 | DGKQ | FBXO9 | DIAPH1 | SRSF11 | LCK |
| GAR1 | DHCR24 | FCRLA | DNAJB12 | STIP1 | LAPTM4B |
| GATAD2A | DIP2B | FCRLB | DNAJC4 | STK35 | LATS1 |
| GBA2 | DLEU2L | FERMT1 | DNASE2 | STRIP2 | LCP1 |
| GCH1 | DLG3 | FERMT2 | DNHD1 | CCDC117 | LDB3 |
| GFOD1 | DLG5 | FLNB | DNM1 | TAF9B | LDHB |
| GGH | DLL1 | FLVCR1 | DNM2 | TARBP1 | LGALS3BP |
| GIGYF1 | DNAAF5 | FMOD | DNMT3A | TBC1D9 | LGALS9 |
| GINS4 | DNAJB1 | FN1 | DOCK10 | TM7SF2 | LGR5 |
| GLA | DNM2 | FTO | DOCK11 | TM7SF3 | LINC00426 |
| GLCCI1 | DOCK1 | ATP2B4 | DOK3 | TMEM106C | LINC01358 |
| GLIPR1 | DOCK10 | FUT7 | DSE | TMEM119 | LMF1 |
| GLRX | DPH1 | FZD3 | DYNC1H1 | TMEM173 | LOC1019274 |
| GLYR1 | DPYSL3 | G2E3 | DYRK1A | TMTC2 | LOC440311 |
| GNB2 | DSE | GABRA3 | E2F2 | TOP1 | LPAR5 |
| GNG5 | DSEL | GABRA4 | E2F6 | TOP2A | LPAR6 |
| GOLIM4 | DYNC1H1 | GAS1 | EBF1 | TPP2 | LPIN1 |
| GOLM1 | DYNC2H1 | GBA2 | ECM1 | TRA2A | LRRFIP2 |

| | | | | | |
|---|---|---|---|---|---|
| GOT2 | DYRK1A | GCN1 | EDEM1 | LGR6 | TSC22D3 |
| GPATCH4 | DYRK2 | GCSAM | EHBP1L1 | TRMT1L | LY6E |
| GPC4 | DZIP1 | GDF11 | EIF1 | TSPAN1 | LY86 |
| GPM6B | EBF1 | GIGYF1 | EIF2S3 | UBR7 | MACROD1 |
| GPR153 | ECHDC3 | GIMAP2 | EIF4EBP1 | TTI1 | MAGI1 |
| GPR173 | EFNB1 | GLCCI1 | ELF1 | ASCC3 | CSRNP1 |
| GPR85 | EIF4A2 | GLIPR1 | ELK1 | UHRF1 | MAPK3 |
| GRINA | EIF4EBP3 | GMCL1 | EMB | USF1 | PPP1R16B |
| GSE1 | ELF1 | GNA11 | EMP1 | USP28 | ARRDC3 |
| GSPT1 | ELOVL6 | GNPAT | EMP3 | PNRC2 | MEF2C |
| GSTK1 | EMP1 | GOLGA4 | ENO1 | WDR5B | MGAT5 |
| GSTZ1 | ENC1 | GPD2 | EPT1 | XPO7 | MIF |
| GTF3A | ENO1 | GPLD1 | ERBB2IP | XRCC2 | MIR3654 |
| GUSB | ENO2 | GPM6B | ERG | ZDHHC6 | MIR4426 |
| GYG1 | ENTPD1 | GPR146 | ESYT1 | ZFC3H1 | MIR663AHG |
| HAP1 | EPHA3 | GPR85 | ESYT2 | ZFP30 | MLLT11 |
| HDAC9 | EPHB4 | GRAMD4 | ETF1 | RRM2 | MMP14 |
| HDGF | ERBB2IP | GRID1 | ETS2 | ZNF738 | MRPL23 |
| HDHD1 | ESD | GTF2I | ETV5 | ZNF780B | MTMR14 |
| HENMT1 | ESYT1 | GUCY1A2 | EVI2B | ZNF782 | MXD4 |
| HGSNAT | ETS2 | GYG1 | EVL | | MYL12A |
| HHLA3 | ETV4 | H19 | EZR | | MYLK |
| HIRA | ETV5 | HAL | FAM101B | | MZT2B |
| HLA-A | EYA1 | KIAA1211 | FAM107B | | NAB2 |
| HLA-B | EYA4 | HDAC7 | FAM117A | | NACA |
| HLA-C | FADS1 | HEATR5A | FAM118A | | NACA2 |
| HMMR | FAM101B | HECW1 | FAM20B | | VAT1 |
| HMOX2 | FAM111B | HELLS | FAM214B | | NIPSNAP1 |
| HNRNPAB | FAM117B | HHLA3 | FAM35A | | NMB |
| HOXA7 | FAM120C | HIBCH | FAM3C | | NME4 |
| HR | FAM129A | HILPDA | FAM49A | | CRMP1 |
| HSBP1 | FAM129B | HK1 | FAM60A | | NTN3 |
| HSF1 | FAM134B | SLC12A7 | FAM8A1 | | MEF2A |
| HSPA8 | FAM134C | HLX | FBXO11 | | ORAI2 |
| HSPB1 | FAM162A | HMMR | FBXO31 | | P2RX1 |
| HSPE1 | FAM171B | HNRNPH1 | FBXO7 | | PACS2 |
| HSPH1 | FAM53B | HPGD | FCGRT | | PAXIP1-AS1 |
| HTATIP2 | FAM63B | HPSE2 | FERMT3 | | PDF |
| HTR7 | FAM89A | HS6ST1 | FGFR1 | | PDK1 |
| HTRA3 | FAM90A1 | HSP90AA1 | FHL1 | | PECAM1 |
| HYAL3 | FAN1 | HSPA4L | FKBP8 | | PELI1 |
| ICAM3 | FARP1 | HSPA8 | FLJ10038 | | PEMT |
| ID2 | FASN | HSPH1 | FMNL2 | | PER3 |
| IFI30 | FBXO16 | HTRA3 | FMNL3 | | PEX10 |
| IFRD1 | FBXO44 | IDH1 | FNBP1 | | HIVEP3 |
| IGFBP7 | FGFBP2 | IER3 | FNDC3B | | PHLPP1 |
| IGLL1 | FGFR1 | IFT172 | FOXN3 | | PIK3IP1 |
| IKZF2 | FHL1 | IGIP | FSCN1 | | PITPNC1 |
| IL12RB1 | FKBP15 | IKBKAP | FUT4 | | PIWIL4 |
| IL1B | FLNA | IKZF2 | G3BP2 | | ADGRL3 |
| IL7R | FMNL3 | IL7R | GAB2 | | PLCB1 |
| IMPDH1 | FNIP2 | ILDR2 | GALNT2 | | PLD4 |
| INPP5D | FOXF2 | ILK | GARS | | PLEKHO1 |
| IPCEF1 | FOXN2 | INCENP | GAS7 | | PLP2 |
| IRAK1 | FOXO1 | INSIG2 | GDPD5 | | POLR2I |
| IRF5 | FOXO3 | INTS8 | GFPT1 | | PPHLN1 |
| ISG20 | FOXP4 | IPO13 | GLG1 | | PPM1H |
| ITGA11 | FRMPD1 | IQSEC1 | GMFB | | PIK3C2B |
| ITGAX | FSCN1 | ISCU | GMFG | | PPP1R15A |
| ITPKA | FSTL1 | ITGA9 | GMPS | | PPP2R5C |
| KAT2A | FTH1 | ITGAL | GNA12 | | PPP3CC |
| KCNMB2 | FUT11 | JMJD1C-AS | GNAI1 | | PRKAB1 |
| KCNMB3 | FYN | KANK2 | GNAQ | | PRKCE |
| KIAA1549 | GAB2 | KANSL1L | GNAS | | PROSER1 |
| KIF26B | GABPA | KCNJ2 | GNB1 | | PRR7 |
| KIR3DX1 | GALM | KCNMB2 | GNB4 | | PRSS57 |
| KLC2 | GAPDH | KCNMB3 | GNG2 | | TXNIP |
| KLHL5 | GATM | KCNQ3 | GNL1 | | PTPN14 |
| KLK1 | GBE1 | KDM2A | GOLPH3 | | PTPN7 |
| LAMTOR4 | GGT7 | CLCN5 | GOT1 | | PTPRK |
| LAPTM4B | GINS3 | KIAA0125 | GPCPD1 | | PVRL2 |
| LARP1 | GJC1 | KIAA0907 | GPSM3 | | PYCR1 |
| LAS1L | GLDC | KIAA0922 | GPT2 | | QSOX1 |
| LAT2 | GM2A | KIAA1456 | GRB2 | | R3HCC1L |
| LCN6 | GNAI3 | KIF15 | GRK6 | | RAB27A |
| LCN8 | GNAQ | KIF20A | GSN | | RABGAP1L |
| LDB3 | GNE | KIF21A | GTF3C4 | | RANBP9 |
| LGALS9 | GNG2 | KIFC1 | GYPC | | RASSF3 |
| LGR5 | GPC1 | VAT1L | H1FX | | RBM3 |
| LILRA2 | GPCPD1 | LAIR1 | H3F3B | | RCBTB2 |
| LILRB4 | GPI | LAMA2 | HAR1A | | RERE |
| LIMK1 | GPR155 | LDLRAD4 | HBEGF | | RGS1 |
| LINC00426 | GPR63 | LFNG | HDAC9 | | RGS13 |
| LINC00462 | GPRC5C | LGI2 | HES6 | | GPCPD1 |
| LINC00997 | GRB10 | LGR4 | HEXA | | FHL1 |
| LINC00998 | GREM1 | LILRB4 | HIF1A | | RHOF |
| LMO2 | GSN | LIMD1 | HIPK3 | | PGK1 |
| OC10192884 | GSTA4 | LIX1L | HIST1H4H | | RNASET2 |
| LOC646762 | GYPC | LMO7 | HIVEP2 | | RNF11 |
| LONRF1 | H1FX | LOC100131 | HLA-A | | RNF126 |
| LRFN1 | HACD2 | LOC101927 | HLA-DOA | | RNF135 |
| LRRC15 | HACD4 | LOC153684 | HLA-DQB1 | | ROBO1 |
| LRWD1 | HACE1 | LOC730101 | HLA-DRB1 | | RPL10 |
| LSM3 | HADH | LPIN2 | HLA-DRB6 | | RPL10A |
| LTC4S | HCFC2 | LRFN1 | HLA-E | | RPL11 |
| LTK | HDAC4 | LRP11 | HMGA1 | | RPL12 |
| LY86 | HEG1 | LRRC27 | HMGA2 | | RPL13 |
| LYAR | HES6 | LRRCC1 | HMHA1 | | RPL15 |
| LYN | HEXIM1 | LTBP1 | HNRNPDL | | RPL18 |
| LYZ | HINT3 | LTBP2 | HOMER1 | | RPL18A |
| MACC1 | HIP1 | LUC7L | HOXA10 | | RPL22 |
| MAD1L1 | HIPK1 | LUC7L3 | HOXA7 | | RPL23 |

| | | | | |
|---|---|---|---|---|
| MAF1 | HIPK3 | LYRM7 | HSD17B12 | RPL24 |
| MAGI1 | HIVEP3 | LYST | HSPA1B | RPL29 |
| MAN1C1 | HK2 | LYZ | HSPA6 | RPL3 |
| MAP2K3 | HLA-DMA | MACC1 | HSPA9 | RPL30 |
| MAPT | HLA-DMB | MAGEF1 | HSPG2 | RPL31 |
| MARS | HLA-DOA | MAGEH1 | HUNK | RPL32 |
| MCM7 | HMGA2 | MAGI1 | HVCN1 | RPL34 |
| MCOLN2 | HMGB3 | MAGI3 | ICAM3 | RPL35 |
| MCTP2 | HMGCR | MARK1 | IGF1R | RPL36 |
| MDH2 | HMGN2 | MAST3 | IGFBP2 | RPL37 |
| MEPCE | HOMER2 | MBTPS1 | IKZF1 | RPL4 |
| MGRN1 | HOPX | MCOLN2 | IL2RG | RPL5 |
| MICALL1 | HOXB7 | ME3 | IMPAD1 | RPLP2 |
| MKRN1 | HSD17B12 | METTL25 | INHBE | RPS11 |
| MLF2 | HSPA13 | METTL3 | INSR | RPS15 |
| MMP24-AS1 | HSPA1A | METTL7A | IRAK1 | RPS2 |
| MOGS | HSPA1B | MEX3A | IRF1 | RPS23 |
| MON1A | HSPA6 | MGAT3 | ITGA2 | RPS24 |
| MPEG1 | HSPB7 | MINK1 | ITGA6 | RPS4X |
| MREG | HSPG2 | MIS18BP1 | ITGB5 | RREB1 |
| MRPL12 | HUNK | MITF | ITPR1 | RSL1D1 |
| MRPL28 | IFITM1 | MKI67 | ITPRIP | RUFY3 |
| MRPL33 | IGF1R | MLEC | IZUMO4 | RUNX1 |
| MRPS17 | IGFBP2 | MLLT4 | JADE3 | DDAH2 |
| MRPS33 | IKZF3 | MME | JAK1 | S100A1 |
| MS4A1 | IKZF4 | BHLHE41 | JMJD1C | S100A10 |
| MTIF2 | IL1RAP | MOGS | JOSD1 | SAMD4A |
| MTIF3 | ING5 | MPEG1 | JUN | HLA-DOA |
| MTMR11 | IQGAP1 | MPHOSPH1 | JUND | SEPN1 |
| MYBBP1A | IQGAP2 | MRE11A | JUP | HOMER2 |
| MYEOV2 | IRS1 | MRPL24 | KAT7 | SH2D2A |
| MYLK | IRS2 | MSL3 | KCNK12 | SH3D21 |
| MYOM1 | ITGA2 | MTIF3 | KIAA0195 | SLA2 |
| MZB1 | ITGA2B | MYEF2 | KIAA0355 | RHOH |
| NAMPT | ITGB1 | MYLK3 | KIF21B | SLC15A3 |
| NCF4 | ITGB2 | MYOF | KLF10 | CCDC69 |
| NDST1 | ITGB5 | MYOM1 | KLF12 | SLC25A26 |
| NDUFAF3 | ITGB8 | NASP | KLF6 | SLC25A29 |
| NDUFAF6 | ITM2C | NBPF1 | KLF9 | SLC2A4RG |
| NDUFB2 | IZUMO4 | NCAPD2 | KLHL15 | SLC38A1 |
| NES | JAK1 | NCAPD3 | KLRF2 | SLC39A8 |
| NEURL1B | JAM2 | NCBP1 | KMT2E | SLC3A2 |
| NFIB | JARID2 | NDFIP1 | KPNA1 | SLC44A2 |
| NFKB2 | JMJD1C | NDRG1 | KPNA4 | SLC52A2 |
| NHP2L1 | JUN | NEURL1B | KSR1 | SLC7A7 |
| NINJ1 | JUP | NEURL4 | LAPTM4B | SMARCA2 |
| NKD2 | KCNMA1 | NFE2 | LAPTM5 | SMDT1 |
| NLGN4X | KCTD12 | NKD2 | LCK | SMYD3 |
| NLRC5 | KCTD18 | NKTR | LCP1 | SNX8 |
| NME1 | KDM3A | NLGN4X | LGALS3BP | H1FX |
| NOG | KDM4B | NOG | LIMK2 | SOX4 |
| NOLC1 | KDM4C | NPEPPS | LINC00938 | SPHK1 |
| NOM1 | KIAA0195 | NR2F6 | LINC01215 | SPP1 |
| NOP16 | KIAA1033 | NRCAM | LINC01237 | SRPK3 |
| NOP2 | KIAA1211 | NREP | LINC01534 | ST3GAL4 |
| NPM3 | KIF3C | NSUN6 | LINC01578 | ST6GAL1 |
| NQO1 | KIF7 | NUDT9 | LINC-PINT | STC2 |
| NR3C2 | KLF13 | NUP210 | LITAF | STK17B |
| NR4A1 | KLF3 | OMA1 | LOC101927497 | SUN2 |
| NRAP | KLF6 | OPN3 | LOC102546299 | SYT1 |
| NSMAF | KLF7 | OSTM1 | LOC728175 | SYTL1 |
| NSMF | KLF9 | OXTR | LPAR6 | GAB2 |
| NSUN5 | KLHL14 | PAQR3 | LPIN1 | TAF1D |
| NUB1 | KLHL15 | PARP2 | LPXN | TAGLN2 |
| NUDT19 | KLHL24 | PATZ1 | LRIG1 | TBC1D15 |
| NUP205 | KLHL9 | PAXBP1 | LRMP | TCF12 |
| OAF | KLRF2 | PCDH18 | LRP1 | TEF |
| OAS2 | KRAS | PCDH9 | LRRC8C | TIE1 |
| ODC1 | KRBA1 | PDCD7 | LRRFIP1 | TMSB4X |
| ORC5 | KSR1 | PDGFRB | LRRFIP2 | TNFRSF1B |
| OSBP2 | LAMP5 | PDIA4 | MAP1LC3B | TNKS1BP1 |
| OSCAR | LASP1 | PER3 | MAP2K2 | TOMM20 |
| P2RX7 | LAT | PEX3 | MAP3K2 | TPD52 |
| PARP12 | LBH | PFKFB3 | MAP4K4 | TPM4 |
| PAXIP1 | LCK | PFKFB4 | MAP7D1 | TRIB3 |
| PCNXL3 | LDOC1L | PFN2 | MAPKAPK2 | TRIM52-AS1 |
| PDAP1 | LGR6 | PGD | MARCKSL1 | TSKU |
| PDCD5 | LINC01108 | PGM2 | MBNL1 | TTF1 |
| PDIA4 | LINC01534 | PHF19 | MBNL3 | UBASH3B |
| PDPN | LINC01578 | PHLPP2 | MBP | UBXN6 |
| PDZD7 | LITAF | PIF1 | MDK | UCN2 |
| PEX3 | LMAN1 | PIGF | MED12L | ENO1 |
| PGP | LMNB1 | PIM2 | MED13L | UNC93B1 |
| PHB | LNPEP | PIP5K1B | MEF2A | UXS1 |
| PIK3CG | OC100129034 | PLA2G12A | MEF2C | VASP |
| PITPNA | OC101928358 | PLAC8 | MEGF10 | CD109 |
| PITPNM2 | LOC93622 | PLCB1 | MFI2 | VPS51 |
| PIWIL4 | LPAR1 | PLD3 | MFN2 | HDAC9 |
| PLCB1 | LPCAT1 | PLD4 | MGAT5 | VWA5A |
| PLD4 | LRIG1 | PLEKHB2 | MICAL1 | WARS |
| PLEK | LRP1 | PLOD1 | MIF | WASF3 |
| PLEKHG3 | LRP5 | PLXND1 | MOAP1 | YDJC |
| PLOD3 | LRRC2 | PODXL | MPC1 | ZMIZ1 |
| PMPCB | LRRFIP1 | POLR2H | MPP6 | ZCCHC7 |
| PMS2P1 | LSP1 | PPP1R9A | MSN | ZFHX3 |
| PNKD | LXN | PPP2R1B | MTHFD1L | LRRFIP1 |
| PODXL | MAML2 | PPP2R5D | MTMR10 | ZNF787 |
| POLR1D | MAML3 | PPWD1 | MTMR12 | ZYX |
| POLR2I | MAN2A1 | PRDM11 | MUL1 | |
| POLR2J | MAP4K5 | PRDM8 | MXRA7 | |
| POLR3H | MAPK14 | PREPL | MYB | |

| | | | | | | | | | |
|---|---|---|---|---|---|---|---|---|---|
| POM121 | MARCHF1 | | | | | | PRICKLE1 | MYBL2 | |
| POMP | MARCKS | | | | | | PRIMPOL | MYH9 | |
| PON2 | MARCKSL1 | | | | | | PRKAR2B | MYL12A | |
| POP7 | MBNL1 | | | | | | PRKCB | MYL12B | |
| POR | MBNL3 | | | | | | PRKCD | MYO7B | |
| POU2AF1 | MCAM | | | | | | PRKG1 | N4BP3 | |
| PPHLN1 | MDGA2 | | | | | | PRKX | NAA25 | |
| PPIB | MDK | | | | | | PRKXP1 | NAB2 | |
| PPM1G | MED12L | | | | | | PRPF38B | NBEAL2 | |
| PPM1H | MED13 | | | | | | PRRC2A | NCF1B | |
| PPP1R35 | MED13L | | | | | | PTPN12 | NCK2 | |
| PPP2R4 | MEF2A | | | | | | PTX3 | NEK6 | |
| PPRC1 | MEGF6 | | | | | | PWWP2B | NFATC3 | |
| PPT1 | MEGF9 | | | | | | PYGL | NFIL3 | |
| PRKAR2B | MESP1 | | | | | | PYGO1 | NIPA1 | |
| PRKCD | METRN | | | | | | RAD54L | NIPA2 | |
| PRKX | MFAP3L | | | | | | RAPGEF2 | NOP10 | |
| PSMB8 | MFGE8 | | | | | | RASGRP2 | NR1D2 | |
| PSMB9 | MFI2 | | | | | | RASSF4 | NR1H2 | |
| PSMC2 | MFSD6 | | | | | | RBM4B | NR3C1 | |
| PSME2 | MGMT | | | | | | RBM5 | NRAS | |
| PSME3 | MIDN | | | | | | RCBTB1 | NRSN2 | |
| PSMG1 | MIR210HG | | | | | | RCCD1 | OAS3 | |
| PSPH | MLC1 | | | | | | REPIN1 | OAZ1 | |
| PTCRA | MMP17 | | | | | | RFC4 | OGFRL1 | |
| PTGES2 | MOB3A | | | | | | RFC5 | OPTN | |
| PTPN12 | MPC1 | | | | | | RGMA | ORAI2 | |
| PTPN7 | MRC2 | | | | | | RGS2 | OSBPL8 | |
| PTPRK | MTFR1L | | | | | | RGS4 | P2RY11 | |
| PTPRU | MTMR10 | | | | | | RHOBTB2 | PAN3 | |
| PTRH2 | MTMR12 | | | | | | RIMBP3 | PAQR4 | |
| PUS7 | MUL1 | | | | | | RMND5B | PARVG | |
| PYCRL | MVB12B | | | | | | RNF144A | PBX3 | |
| PYGL | MXRA8 | | | | | | RNF170 | PCBP4 | |
| RAB31 | MYC | | | | | | RNF20 | PCDHGC3 | |
| RABEP1 | MYH10 | | | | | | RORA | PCK2 | |
| RABGAP1L | MYO15B | | | | | | RPAP1 | PDE7A | |
| RAG2 | N4BP2 | | | | | | RPGRIP1L | PDLIM7 | |
| RAP1GAP2 | NAP1L5 | | | | | | P4HA1 | PELI1 | |
| RAPGEF3 | NCK2 | | | | | | RUFY1 | PELI2 | |
| RASGRP2 | NCR3LG1 | | | | | | RUNX3 | PER2 | |
| RBM28 | NDRG1 | | | | | | S100A4 | PEX10 | |
| RCCD1 | NDUFA6 | | | | | | SAFB | PGK1 | |
| REEP4 | NEDD4L | | | | | | SAMD9L | PHGDH | |
| REPIN1 | NEK6 | | | | | | SAMHD1 | PHIP | |
| RFC3 | NEK7 | | | | | | SAPCD2 | PHLPP1 | |
| RFX5 | NELL1 | | | | | | SART3 | PI16 | |
| RFXAP | NELL2 | | | | | | SCAMP1 | PI4KAP2 | |
| RHOC | NFATC3 | | | | | | SCCPDH | PIK3C2B | |
| RNF170 | NFIC | | | | | | SDCCAG8 | PIK3CG | |
| RNFT1 | NFYA | | | | | | SEL1L3 | PIP4K2A | |
| RNH1 | NHSL1 | | | | | | SELPLG | PITPNC1 | |
| ROMO1 | NIPA1 | | | | | | SEMA4B | PKDCC | |
| RPP38 | NKX3-1 | | | | | | SEMA4C | PLCH1 | |
| RPS6KA5 | NPC1 | | | | | | REEP3 | PLEKHO1 | |
| RRS1 | NPR1 | | | | | | SETD1B | PLXNC1 | |
| RSBN1L | NPW | | | | | | SFR1 | POMP | |
| RTCB | NR2C2 | | | | | | SFXN2 | PPM1A | |
| S100A11 | NR3C1 | | | | | | SGOL2 | PPM1F | |
| SAPCD2 | NRSN2 | | | | | | SH2D3C | PPP1R15A | |
| SBDS | NUCB2 | | | | | | CD38 | PPP1R16B | |
| SCARB1 | NUDT10 | | | | | | SHISA2 | PPP2R5C | |
| SCARF1 | OAS3 | | | | | | SIGLEC10 | PPP3CA | |
| SCCPDH | OCIAD2 | | | | | | SKP2 | PPP3CC | |
| SCYL1 | OPHN1 | | | | | | SLA | PPP3R1 | |
| SDR39U1 | OSBPL8 | | | | | | CDCA3 | PPP4R1 | |
| SEC11A | OTUD1 | | | | | | SLC16A10 | PPP6R1 | |
| SEL1L3 | P4HA1 | | | | | | SLC25A25-A | PRDX1 | |
| SEPTIN10 | PABPC4L | | | | | | SLC25A30 | PRKAA1 | |
| SERPINB12 | PALLD | | | | | | SLC25A41 | PRKAB1 | |
| SFXN2 | PARD6G | | | | | | SLC26A9 | PRKCE | |
| SFXN3 | PARP15 | | | | | | SLC27A4 | PRKCQ | |
| SGCE | PAX5 | | | | | | HK2 | PRKD2 | |
| SH3BGRL | PCBP4 | | | | | | SLC2A9 | PRNP | |
| SH3TC1 | PCDHGA11 | | | | | | SLC30A4 | PROM1 | |
| SLA | PCDHGA12 | | | | | | SLC40A1 | PRSS12 | |
| SLC12A9 | PCDHGC3 | | | | | | SLC48A1 | PSAP | |
| SLC25A13 | PCED1A | | | | | | SLC4A4 | PSAT1 | |
| SLC25A22 | PCGF2 | | | | | | SLC4A8 | PSMB10 | |
| SLC25A23 | PCGF6 | | | | | | SLC5A3 | PSMD7 | |
| SLC25A32 | PCYT1B | | | | | | SLC5A6 | PSME4 | |
| SLC25A41 | PDGFD | | | | | | SLC8A1 | PTBP3 | |
| SLC29A1 | PDIK1L | | | | | | SLIT2 | PTGS1 | |
| SLC35B4 | PDK3 | | | | | | SLITRK6 | PTK2 | |
| SLC35C1 | PELI2 | | | | | | SMAD3 | PTP4A3 | |
| SLC35C2 | PFKL | | | | | | SMC1A | PTPN1 | |
| SLC39A13 | PGBD5 | | | | | | SMIM14 | PTPN7 | |
| SLC39A14 | PGK1 | | | | | | SNCB | PTPN9 | |
| SLC48A1 | PGM1 | | | | | | SNRNP40 | PVT1 | |
| SLC4A2 | PHF14 | | | | | | SNTB2 | PWAR5 | |
| SLC4A8 | PHF2 | | | | | | SORBS2 | PXK | |
| SLC52A2 | PHIP | | | | | | SORL1 | PYCR1 | |
| SLC5A6 | PHKB | | | | | | GSTCD | R3HDM4 | |
| SLC7A6 | PIK3C2B | | | | | | SPAG5 | RABGAP1L | |
| SLC7A7 | PIK3R6 | | | | | | SPDL1 | RAI1 | |
| SMAD3 | PIM1 | | | | | | SPIB | RAP1B | |
| SMIM3 | PKM | | | | | | SPOCK3 | RASA3 | |
| SND1 | PLAUR | | | | | | SPP1 | RASSF1 | |
| SNHG6 | PLCB2 | | | | | | SPTLC3 | RASSF3 | |
| SNX14 | PLCG1 | | | | | | SRGAP3 | RBM3 | |
| SOX4 | PLCH1 | | | | | | SRGN | RBM38 | |

| | | | | | | | | | |
|---|---|---|---|---|---|---|---|---|---|
| SPA17 | PLEKHB1 | | | | | | SRI | RBMS1 | |
| SPG20 | PLOD2 | | | | | | SRSF1 | RCBTB2 | |
| SPIRE1 | PLXNA1 | | | | | | SSBP2 | RDX | |
| SPRYD3 | PLXNB1 | | | | | | ST3GAL6 | RELB | |
| SPTBN2 | PLXNB2 | | | | | | STAU2 | RERE | |
| SRGN | PNRC1 | | | | | | STEAP3 | RFTN1 | |
| SRI | PNRC2 | | | | | | STIP1 | RGL1 | |
| SRM | PPARA | | | | | | STRA13 | RGS1 | |
| SRPK2 | PPFIA4 | | | | | | SUSD6 | RHOA | |
| SSBP1 | PPM1M | | | | | | SYNPO | RHOB | |
| STAG3L2 | PPP1CB | | | | | | TADA2A | RICTOR | |
| STAU2 | PPP1R16B | | | | | | TAF1A | RIMS3 | |
| STEAP3 | PPP3CA | | | | | | TBC1D30 | RLTPR | |
| STIM2 | PRDX2 | | | | | | TBC1D9 | RMND5A | |
| STS | PREX1 | | | | | | TBCCD1 | RNASET2 | |
| STYXL1 | PRKAA1 | | | | | | TBCK | RNF11 | |
| SUOX | PRKCQ | | | | | | TCOF1 | RNF139 | |
| SUSD6 | PROM1 | | | | | | TCONS_000 | RNF187 | |
| SYNGR2 | PRSS12 | | | | | | TECPR1 | RNF213 | |
| SYNPO2L | PRUNE2 | | | | | | TENM3 | RNF220 | |
| SYVN1 | PSD3 | | | | | | TENM4 | ROBO1 | |
| TAF6 | PSIP1 | | | | | | TET2 | RPL10 | |
| TAP2 | PTBP3 | | | | | | ZNF395 | RPL22 | |
| TBL2 | PTCH1 | | | | | | TFRC | RPL4 | |
| TCL1A | PTK7 | | | | | | TGFA | RPS6KA3 | |
| TCOF1 | PTP4A3 | | | | | | THSD7A | RRAS2 | |
| TEC | PTPRJ | | | | | | TIMMDC1 | RUNX1 | |
| TECPR1 | PTPRR | | | | | | TJP2 | RUNX2 | |
| TENM4 | PVT1 | | | | | | TLR10 | SARS | |
| TES | PWAR5 | | | | | | TLR4 | SATB1 | |
| TEX19 | PXDN | | | | | | TMEM106C | SBF2-AS1 | |
| TEX40 | QKI | | | | | | TMEM119 | SCPEP1 | |
| TIE1 | RAB28 | | | | | | TMEM165 | SELT | |
| TIGD5 | RAB34 | | | | | | TMEM170B | SEMA3A | |
| TIMP2 | RAI1 | | | | | | TMEM173 | SENP2 | |
| TMCC1-AS1 | RAI14 | | | | | | TMEM50B | SEPTIN1 | |
| TMEM119 | RAPGEF6 | | | | | | TOB1-AS1 | SEPTIN6 | |
| TMEM120B | RASAL2 | | | | | | TONSL | SEPTIN9 | |
| TMEM147 | RBSN | | | | | | TOP1 | SERINC5 | |
| TMEM209 | RC3H2 | | | | | | TOP2A | SERPINB8 | |
| TMEM248 | RCHY1 | | | | | | TP53I11 | SESN2 | |
| TMEM8A | RCSD1 | | | | | | TPP2 | SESTD1 | |
| TNFRSF8 | RDX | | | | | | TRIM25 | SETX | |
| TNPO3 | REC8 | | | | | | TRIM4 | SH2D4B | |
| TOB2P1 | REEP3 | | | | | | TRIM56 | SH3BP2 | |
| TOP1MT | RENBP | | | | | | TRIM58 | SH3GL1 | |
| TPRN | RHBDD1 | | | | | | TRMU | SH3KBP1 | |
| TRAIP | RHOA | | | | | | TSPAN12 | SHMT2 | |
| TRAP1 | RHOB | | | | | | TST | SHOC2 | |
| TRIM24 | RHOBTB1 | | | | | | TTC21B | SIAH2 | |
| TRIM72 | RHOBTB3 | | | | | | TTC28 | SIRT7 | |
| TRIP6 | RHOH | | | | | | TTI1 | SKA2 | |
| TRMT10B | RIF1 | | | | | | TTPA | SKIL | |
| TRMU | RIT1 | | | | | | TUBB4A | SLAIN1 | |
| TRPM4 | RLF | | | | | | UBE2C | SLAMF1 | |
| TSPAN12 | RNASE6 | | | | | | UBE4B | SLC16A7 | |
| TSPAN33 | RNASET2 | | | | | | UBFD1 | SLC17A5 | |
| TSPAN4 | RNF125 | | | | | | UGDH | SLC1A5 | |
| TST | RNF130 | | | | | | UGT3A2 | SLC38A1 | |
| TTC26 | RNF145 | | | | | | ULK3 | SLC39A8 | |
| TTC7A | RNF150 | | | | | | USF1 | SLC3A2 | |
| TXNRD2 | RNF213 | | | | | | USP28 | SLC41A3 | |
| UACA | RNF220 | | | | | | UST | SLC43A1 | |
| UBE2L6 | RNF24 | | | | | | UTP14A | SLC4A7 | |
| UBL3 | ROR1 | | | | | | VDAC3 | SLC7A1 | |
| UCN2 | RPH3AL | | | | | | VEGFC | SLC7A11 | |
| UNC119 | RPS6KA3 | | | | | | VGLL3 | SLC7A5 | |
| UQCC2 | RRAS2 | | | | | | TUBA1A | SLC7A7 | |
| USMG5 | RRM2 | | | | | | VNN1 | SMAGP | |
| USP30-AS1 | RUNX2 | | | | | | VPREB3 | SMARCA2 | |
| VAMP8 | RXRA | | | | | | VSIG10 | SMIM7 | |
| VDAC3 | RYBP | | | | | | WSB1 | SNORA71B | |
| VKORC1L1 | S100A6 | | | | | | ZBTB40 | SNX10 | |
| VPREB3 | SACS | | | | | | ZC3H12B | SNX30 | |
| VSIG10L | SALL2 | | | | | | ZFHX2 | SNX5 | |
| WASF3 | SATB1 | | | | | | ZFP62 | SNX8 | |
| WBSCR22 | SBF1 | | | | | | ZGRF1 | SORCS2 | |
| WWC3 | SBF2-AS1 | | | | | | ZMYND19 | SOWAHD | |
| YDJC | SCD | | | | | | ZNF106 | SPNS3 | |
| YWHAG | SCD5 | | | | | | ZNF189 | SPRY4 | |
| ZC3H12D | SCN9A | | | | | | ZNF211 | SREBF2 | |
| ZC3HAV1 | SECISBP2L | | | | | | ZNF286A | SRSF5 | |
| ZC3HC1 | SEMA3A | | | | | | ZNF311 | SRSF6 | |
| ZCWPW1 | SEMA3F | | | | | | ZNF318 | ST3GAL1 | |
| ZDHHC2 | SEMA6C | | | | | | ZNF37BP | ST6GAL1 | |
| ZFHX3 | SENP6 | | | | | | ZNF436 | STAT3 | |
| ZFPM2 | SEPTIN8 | | | | | | ZNF445 | STAT5A | |
| ZGRF1 | SERINC2 | | | | | | ZNF45 | STIM2 | |
| ZMYND19 | SERINC5 | | | | | | ZNF503-AS2 | STK10 | |
| ZNF117 | SERPINB1 | | | | | | ZNF547 | STK17A | |
| ZNF208 | SERPINE2 | | | | | | ZNF565 | STK17B | |
| ZNF223 | SESN1 | | | | | | ZNF608 | STRN4 | |
| ZNF440 | SESN3 | | | | | | ZNF616 | STT3B | |
| ZNF45 | SF3B1 | | | | | | ZNF638 | SUCLG2 | |
| ZNHIT1 | SFMBT2 | | | | | | ZNF704 | SUMO3 | |
| ZYX | SFT2D2 | | | | | | ZNF730 | SUN2 | |
| | SGK1 | | | | | | ZNF737 | SUPT3H | |
| | SH3YL1 | | | | | | ZNF738 | SYT1 | |
| | SHFM1 | | | | | | ZNF804A | SYTL1 | |
| | SHOC2 | | | | | | ZNF91 | TAB3 | |
| | SIGIRR | | | | | | ZNRD1 | TACC1 | |

| | | | | | | | | |
|---|---|---|---|---|---|---|---|---|
| SIK2 | | | | | | ZSCAN31 | TAF9 | |
| SIRPA | | | | | | ZWILCH | TAGLN2 | |
| SIRT1 | | | | | | ZWINT | TAPT1 | |
| SIT1 | | | | | | | TARS | |
| SLAMF1 | | | | | | | TBC1D14 | |
| SLAMF8 | | | | | | | TBKBP1 | |
| SLC12A6 | | | | | | | TBL1X | |
| SLC12A7 | | | | | | | TBL1XR1 | |
| SLC16A2 | | | | | | | TCTA | |
| SLC16A3 | | | | | | | TGFBR1 | |
| SLC23A2 | | | | | | | TGFBR2 | |
| SLC25A1 | | | | | | | TGFBRAP1 | |
| SLC26A2 | | | | | | | TM6SF1 | |
| SLC27A2 | | | | | | | TM9SF3 | |
| SLC2A3 | | | | | | | TMC8 | |
| SLC2A5 | | | | | | | TMED2 | |
| SLC37A2 | | | | | | | TMEM167B | |
| SLC4A7 | | | | | | | TMEM87A | |
| SLC8A3 | | | | | | | TNFAIP3 | |
| SLC9A3R1 | | | | | | | TNFRSF1B | |
| SLCO4C1 | | | | | | | TNKS1BP1 | |
| SMAGP | | | | | | | TNRC18 | |
| SMIM15 | | | | | | | TOMM20 | |
| SNN | | | | | | | TPBG | |
| SNX18 | | | | | | | TPD52 | |
| SNX20 | | | | | | | TPH2 | |
| SNX30 | | | | | | | TPM4 | |
| SNX9 | | | | | | | TRAM2 | |
| SORBS1 | | | | | | | TRDMT1 | |
| SORBS3 | | | | | | | TRHDE | |
| SORCS2 | | | | | | | TRIB3 | |
| SOS1 | | | | | | | TRIM52-AS1 | |
| SOWAHD | | | | | | | TRPM2 | |
| SP1 | | | | | | | TSC22D3 | |
| SP7 | | | | | | | TSPAN13 | |
| SPARC | | | | | | | TSPAN17 | |
| SPNS2 | | | | | | | TTC38 | |
| SPNS3 | | | | | | | TUBA4A | |
| SPRY2 | | | | | | | TWISTNB | |
| SPTLC2 | | | | | | | TWSG1 | |
| SREBF2 | | | | | | | TXNIP | |
| SREK1 | | | | | | | UBALD2 | |
| SSFA2 | | | | | | | UBASH3B | |
| ST8SIA5 | | | | | | | UBE2E1 | |
| STAG2 | | | | | | | UBE2J1 | |
| STARD13 | | | | | | | UBQLN1 | |
| STAT3 | | | | | | | UNC13B | |
| STAT5A | | | | | | | UNC5B | |
| STK10 | | | | | | | UNC93B1 | |
| STX6 | | | | | | | USP1 | |
| SUCLG2 | | | | | | | USP3 | |
| SUPT3H | | | | | | | USP38 | |
| SYN1 | | | | | | | VASP | |
| SYNJ2 | | | | | | | VAV3 | |
| SYNRG | | | | | | | VEGFB | |
| TAB3 | | | | | | | VPS37B | |
| TACC1 | | | | | | | VPS51 | |
| TAPT1 | | | | | | | WAPAL | |
| TARSL2 | | | | | | | WARS | |
| TBKBP1 | | | | | | | WDR13 | |
| TESK1 | | | | | | | WDR45B | |
| TEX30 | | | | | | | WIPF1 | |
| TFCP2 | | | | | | | XBP1 | |
| TGFB1 | | | | | | | XYLT1 | |
| THEMIS2 | | | | | | | YARS | |
| TIAM1 | | | | | | | YES1 | |
| TIMP1 | | | | | | | YPEL5 | |
| TK1 | | | | | | | YTHDF1 | |
| TLE3 | | | | | | | ZBTB2 | |
| TLN1 | | | | | | | ZC3H12C | |
| TLR1 | | | | | | | ZCCHC7 | |
| TLR9 | | | | | | | ZFAND3 | |
| TMC8 | | | | | | | ZNF217 | |
| TMED2 | | | | | | | ZNF274 | |
| TMEM136 | | | | | | | ZNF423 | |
| TMEM185B | | | | | | | ZNF516 | |
| TMEM216 | | | | | | | ZNRF1 | |
| TMEM245 | | | | | | | | |
| TMEM45A | | | | | | | | |
| TMPO | | | | | | | | |
| TMSB15A | | | | | | | | |
| TNRC18 | | | | | | | | |
| TNRC6B | | | | | | | | |
| TNS1 | | | | | | | | |
| TOLLIP-AS1 | | | | | | | | |
| TOM1L1 | | | | | | | | |
| TOX | | | | | | | | |
| TPCN1 | | | | | | | | |
| TRIB2 | | | | | | | | |
| TRIM14 | | | | | | | | |
| TRIM9 | | | | | | | | |
| TRO | | | | | | | | |
| TRPM2 | | | | | | | | |
| TRPT1 | | | | | | | | |
| TRRAP | | | | | | | | |
| TRUB1 | | | | | | | | |
| TSC22D1 | | | | | | | | |
| TSC22D3 | | | | | | | | |
| TSPAN14 | | | | | | | | |
| TSPYL1 | | | | | | | | |
| TSPYL4 | | | | | | | | |

| | | | | | | | | |
|---|---|---|---|---|---|---|---|---|
| TTC33 | | | | | | | | |
| TTC38 | | | | | | | | |
| TTLL5 | | | | | | | | |
| TTYH3 | | | | | | | | |
| TUBA1A | | | | | | | | |
| TUBA8 | | | | | | | | |
| TUBGCP3 | | | | | | | | |
| TUBGCP5 | | | | | | | | |
| TXNIP | | | | | | | | |
| UBR7 | | | | | | | | |
| UBXN7 | | | | | | | | |
| UFL1 | | | | | | | | |
| UNC13B | | | | | | | | |
| UNC5B | | | | | | | | |
| USE1 | | | | | | | | |
| VAMP5 | | | | | | | | |
| VAT1 | | | | | | | | |
| VAT1L | | | | | | | | |
| VAV1 | | | | | | | | |
| VAV3 | | | | | | | | |
| VCAN | | | | | | | | |
| VEGFA | | | | | | | | |
| VEGFB | | | | | | | | |
| VIM | | | | | | | | |
| VKORC1 | | | | | | | | |
| VPS36 | | | | | | | | |
| WAPAL | | | | | | | | |
| WASF2 | | | | | | | | |
| WDFY1 | | | | | | | | |
| WDFY3 | | | | | | | | |
| WDR66 | | | | | | | | |
| WDR76 | | | | | | | | |
| WDTC1 | | | | | | | | |
| YES1 | | | | | | | | |
| YPEL5 | | | | | | | | |
| ZAP70 | | | | | | | | |
| ZBTB1 | | | | | | | | |
| ZBTB17 | | | | | | | | |
| ZBTB41 | | | | | | | | |
| ZC3H12C | | | | | | | | |
| ZC4H2 | | | | | | | | |
| ZCCHC24 | | | | | | | | |
| ZDBF2 | | | | | | | | |
| ZFAND4 | | | | | | | | |
| ZMIZ1 | | | | | | | | |
| ZMYM2 | | | | | | | | |
| ZNF114 | | | | | | | | |
| ZNF160 | | | | | | | | |
| ZNF217 | | | | | | | | |
| ZNF24 | | | | | | | | |
| ZNF264 | | | | | | | | |
| ZNF292 | | | | | | | | |
| ZNF337-AS1 | | | | | | | | |
| ZNF395 | | | | | | | | |
| ZNF423 | | | | | | | | |
| ZNF451 | | | | | | | | |
| ZNF629 | | | | | | | | |
| ZNF649 | | | | | | | | |
| ZNF675 | | | | | | | | |
| ZNF709 | | | | | | | | |
| ZNF711 | | | | | | | | |
| ZNF714 | | | | | | | | |
| ZNF718 | | | | | | | | |
| ZNF84 | | | | | | | | |
| ZNRF1 | | | | | | | | |
| ZP3 | | | | | | | | |

# Supplementary Table S2: Normalized cell viability of the various concentrations of each compound

| Compound | Origen | SEM 1nM | SEM$^{PINO_RES}$ 1nM | SEM 10 nM | SEM 10 nM | SEM$^{PINO_RES}$ 10 nM | SEM$^{PINO_RES}$ 10 nM | SEM 100 nM | SEM 100 nM | SEM$^{PINO_RES}$ 100 nM | SEM$^{PINO_RES}$ 100 nM | SEM 1 µM | SEM 1 µM | SEM$^{PINO_RES}$ 1 µM | SEM$^{PINO_RES}$ 1 µM | SEM 10uM | SEM$^{PINO_RES}$ 10uM |
|---|---|---|---|---|---|---|---|---|---|---|---|---|---|---|---|---|---|
| MLN4924 | added drug, Selleckchem | | | 106 | 102 | 99 | 106 | 91 | 123 | 97 | 107 | 3 | 6 | 64 | 88 | | |
| AMG-337 | added drug, Selleckchem | | | 80 | 88 | 98 | 96 | 76 | 108 | 91 | 94 | 74 | 112 | 85 | 94 | | |
| C-DIM12 | added drug, Selleckchem | | | 96 | 95 | 102 | 98 | 90 | 116 | 97 | 101 | 73 | 108 | 81 | 89 | | |
| LBH589 | added drug, Selleckchem | | | 76 | 78 | 90 | 92 | 43 | 59 | 59 | 70 | 0 | 0 | 0 | 0 | | |
| Ipatasertib | added drug, Selleckchem | | | 106 | 100 | 103 | 104 | 95 | 125 | 97 | 112 | 68 | 75 | 83 | 95 | | |
| MK-2206 | added drug, Selleckchem | | | 101 | 104 | 100 | 100 | 88 | 99 | 90 | 92 | 38 | 43 | 69 | 57 | | |
| AZD8055 | added drug, Selleckchem | | | 72 | 76 | 87 | 89 | 38 | 50 | 52 | 60 | 17 | 30 | 29 | 26 | | |
| SBI-0640756 | added drug, Selleckchem | | | 106 | 102 | 104 | 102 | 97 | 115 | 97 | 104 | 1 | 0 | 0 | 0 | | |
| iBET151 | added drug, Selleckchem | | | 86 | 84 | 93 | 87 | 60 | 72 | 61 | 66 | 0 | 0 | 1 | 0 | | |
| Palbociclib | added drug, Selleckchem | | | 103 | 102 | 104 | 100 | 82 | 95 | 92 | 97 | 25 | 41 | 46 | 43 | | |
| AZD6738 | added drug, Selleckchem | | | 99 | 104 | 103 | 100 | 75 | 106 | 98 | 101 | 6 | 12 | 27 | 35 | | |
| Talazoparib | added drug, Selleckchem | | | 66 | 86 | 86 | 83 | 52 | 70 | 68 | 78 | 15 | 15 | 43 | 63 | | |
| EPZ5676 | added drug, Selleckchem | | | 85 | 95 | 93 | 91 | 87 | 98 | 89 | 94 | 86 | 120 | 94 | 92 | | |
| VE821 | added drug, Selleckchem | | | 115 | 111 | 104 | 95 | 104 | 109 | 100 | 102 | 37 | 38 | 71 | 59 | | |
| MRT68921 | added drug, Selleckchem | | | 93 | 97 | 98 | 90 | 85 | 101 | 92 | 85 | 22 | 9 | 48 | 34 | | |
| Abemaciclib | added drug, Selleckchem | | | 109 | 112 | 109 | 101 | 86 | 96 | 95 | 108 | 23 | 35 | 54 | 42 | | |
| GSK-J4 HCl | added drug, Selleckchem | | | 109 | 108 | 110 | 104 | 106 | 126 | 103 | 105 | 97 | 12 | 90 | 66 | | |
| ANA-12 | added drug, Selleckchem | | | 88 | 104 | 105 | 98 | 94 | 113 | 92 | 102 | 68 | 93 | 91 | 96 | | |
| CYC-065 | added drug, Selleckchem | | | 106 | 105 | 105 | 105 | 106 | 106 | 105 | 104 | 23 | 175 | 77 | 160 | | |
| JQ1 | added drug, Selleckchem | | | 111 | 105 | 115 | 107 | 49 | 114 | 54 | 106 | 0 | 180 | 0 | 161 | | |
| ABT199 | added drug, Selleckchem | | | 4 | 82 | 1 | 30 | 0 | 6 | 0 | 0 | 0 | 0 | 0 | 0 | | |
| ABT-263 | added drug, Selleckchem | | | 91 | 91 | 83 | 87 | 5 | 52 | 1 | 12 | 0 | 0 | 0 | 0 | | |
| Pimelic Diphenylamide 106 | Cayman Epigenetic screening library | | | 84 | 90 | 89 | 93 | 85 | 106 | 89 | 93 | 57 | 106 | 80 | 95 | | |
| 3-Deazaneplanocin A | Cayman Epigenetic screening library | | | 75 | 99 | 90 | 93 | 32 | 54 | 52 | 69 | 8 | 16 | 11 | 9 | | |
| 2,4-DPD | Cayman Epigenetic screening library | | | 76 | 97 | 85 | 96 | 77 | 103 | 88 | 98 | 73 | 130 | 78 | 98 | | |
| 2-PCPA (hydrochloride) | Cayman Epigenetic screening library | | | 78 | 93 | 89 | 95 | 75 | 110 | 91 | 92 | 71 | 114 | 85 | 93 | | |
| I-DET | Cayman Epigenetic screening library | | | 76 | 95 | 95 | 95 | 59 | 89 | 77 | 91 | 0 | 0 | 5 | 4 | | |
| UNC0638 | Cayman Epigenetic screening library | | | 69 | 95 | 96 | 91 | 74 | 111 | 96 | 95 | 74 | 116 | 86 | 92 | | |
| 4-iodo-SAHA | Cayman Epigenetic screening library | | | 74 | 99 | 87 | 95 | 68 | 107 | 88 | 104 | 0 | 0 | 0 | -1 | | |
| UNC0321 (trifluoroacetate salt) | Cayman Epigenetic screening library | | | 78 | 97 | 89 | 94 | 67 | 109 | 80 | 97 | 0 | 2 | 2 | 10 | | |
| isoliquiritigenin | Cayman Epigenetic screening library | | | 77 | 96 | 93 | 97 | 72 | 104 | 86 | 97 | 1 | 12 | 12 | 25 | | |
| CAY10603 | Cayman Epigenetic screening library | | | 80 | 100 | 98 | 89 | 72 | 105 | 83 | 89 | 0 | 0 | 1 | 2 | | |
| Pictilisib | Cayman Epigenetic screening library | | | 96 | 94 | 101 | 101 | 69 | 99 | 75 | 94 | 23 | 37 | 50 | 46 | | |
| (S)-HDAC-42 | Cayman Epigenetic screening library | | | 86 | 90 | 84 | 85 | 46 | 75 | 49 | 63 | 0 | 0 | 0 | -1 | | |
| trans-Resveratrol | Cayman Epigenetic screening library | | | 91 | 88 | 93 | 90 | 90 | 98 | 91 | 93 | 104 | 133 | 94 | 93 | | |
| DMOG | Cayman Epigenetic screening library | | | 89 | 93 | 94 | 89 | 90 | 99 | 89 | 96 | 102 | 135 | 92 | 98 | | |
| Cl-Amidine | Cayman Epigenetic screening library | | | 81 | 95 | 89 | 92 | 83 | 99 | 87 | 92 | 88 | 128 | 85 | 101 | | |
| Sodium Butyrate | Cayman Epigenetic screening library | | | 85 | 94 | 95 | 91 | 85 | 102 | 93 | 94 | 91 | 122 | 93 | 96 | | |
| Anacardic Acid | Cayman Epigenetic screening library | | | 86 | 98 | 94 | 99 | 83 | 102 | 86 | 97 | 91 | 112 | 92 | 95 | | |
| Tubastatin A (trifluoroacetate salt) | Cayman Epigenetic screening library | | | 88 | 95 | 91 | 95 | 85 | 101 | 92 | 94 | 64 | 77 | 81 | 92 | | |
| Nicotinamide | Cayman Epigenetic screening library | | | 90 | 99 | 96 | 89 | 90 | 102 | 86 | 92 | 98 | 111 | 88 | 97 | | |
| Zebularine | Cayman Epigenetic screening library | | | 87 | 99 | 97 | 94 | 88 | 97 | 93 | 96 | 98 | 126 | 94 | 90 | | |
| S-Adenosylhomocysteine | Cayman Epigenetic screening library | | | 92 | 96 | 92 | 85 | 85 | 98 | 87 | 89 | 65 | 104 | 69 | 89 | | |
| MS-275 | Cayman Epigenetic screening library | | | 80 | 102 | 94 | 94 | 67 | 93 | 76 | 78 | 0 | 0 | 1 | -1 | | |
| Trichostatin A | Cayman Epigenetic screening library | | | 83 | 93 | 92 | 93 | 39 | 90 | 73 | 86 | 0 | 0 | 0 | 0 | | |
| CCG-100602 | Cayman Epigenetic screening library | | | 84 | 96 | 93 | 94 | 77 | 104 | 92 | 95 | 92 | 128 | 90 | 93 | | |
| CAY10398 | Cayman Epigenetic screening library | | | 81 | 88 | 93 | 94 | 78 | 104 | 88 | 90 | 81 | 117 | 81 | 95 | | |
| Oxamflatin | Cayman Epigenetic screening library | | | 83 | 99 | 93 | 98 | 74 | 108 | 80 | 89 | 0 | 2 | 1 | 3 | | |
| Salermide | Cayman Epigenetic screening library | | | 82 | 95 | 99 | 90 | 79 | 102 | 97 | 104 | 86 | 146 | 98 | 106 | | |
| Garcinol | Cayman Epigenetic screening library | | | 82 | 96 | 98 | 98 | 81 | 103 | 94 | 94 | 89 | 119 | 99 | 97 | | |
| BIX01294 (hydrochloride hydrate) | Cayman Epigenetic screening library | | | 78 | 91 | 96 | 89 | 80 | 95 | 92 | 87 | 88 | 119 | 84 | 84 | | |
| Valproic Acid (sodium salt) | Cayman Epigenetic screening library | | | 80 | 97 | 87 | 91 | 80 | 100 | 88 | 94 | 88 | 125 | 88 | 102 | | |
| Splitomicin | Cayman Epigenetic screening library | | | 80 | 96 | 91 | 94 | 77 | 102 | 90 | 93 | 89 | 136 | 89 | 97 | | |
| HNHA | Cayman Epigenetic screening library | | | 101 | 89 | 94 | 93 | 100 | 98 | 93 | 97 | 116 | 124 | 109 | 96 | | |
| Benzenebutanoic Acid (sodium salt) | Cayman Epigenetic screening library | | | 100 | 91 | 92 | 93 | 100 | 93 | 90 | 90 | 103 | 136 | 101 | 93 | | |
| (-)-Neplanocin A | Cayman Epigenetic screening library | | | 84 | 95 | 89 | 97 | 7 | 22 | 7 | 19 | 4 | 13 | 4 | 3 | | |
| 1-Naphthoic Acid | Cayman Epigenetic screening library | | | 100 | 100 | 94 | 94 | 93 | 109 | 89 | 94 | 97 | 117 | 91 | 94 | | |
| 2',3',5'-triacetyl-5-Azacytidine | Cayman Epigenetic screening library | | | 96 | 91 | 96 | 97 | 103 | 103 | 89 | 90 | 111 | 105 | 91 | 98 | | |
| UNC0224 | Cayman Epigenetic screening library | | | 93 | 95 | 97 | 96 | 98 | 97 | 87 | 95 | 87 | 107 | 91 | 109 | | |
| Ellagic Acid | Cayman Epigenetic screening library | | | 101 | 98 | 96 | 96 | 91 | 106 | 92 | 88 | 91 | 111 | 101 | 91 | | |
| Suramin (sodium salt) | Cayman Epigenetic screening library | | | 100 | 88 | 95 | 90 | 93 | 94 | 90 | 84 | 110 | 119 | 96 | 79 | | |
| Tenovin-1 | Cayman Epigenetic screening library | | | 103 | 91 | 93 | 91 | 95 | 91 | 88 | 94 | 68 | 92 | 76 | 75 | | |
| CBHA | Cayman Epigenetic screening library | | | 101 | 95 | 93 | 93 | 76 | 95 | 63 | 77 | 1 | 2 | 1 | 0 | | |
| GDC0994 | Cayman Epigenetic screening library | | | 106 | 110 | 106 | 100 | 104 | 108 | 101 | 98 | 86 | 105 | 94 | 90 | | |
| Lorlatinib | Cayman Epigenetic screening library | | | 102 | 96 | 98 | 86 | 94 | 93 | 89 | 85 | 85 | 96 | 85 | 87 | | |
| RG-108 | Cayman Epigenetic screening library | | | 90 | 93 | 94 | 93 | 91 | 96 | 94 | 93 | 121 | 136 | 101 | 97 | | |
| CAY10433 | Cayman Epigenetic screening library | | | 93 | 91 | 95 | 90 | 86 | 103 | 96 | 97 | 88 | 115 | 98 | 95 | | |
| Sinefungin | Cayman Epigenetic screening library | | | 91 | 89 | 89 | 87 | 87 | 90 | 90 | 88 | 78 | 111 | 95 | 92 | | |
| Piceatannol | Cayman Epigenetic screening library | | | 95 | 90 | 92 | 97 | 87 | 92 | 90 | 90 | 100 | 111 | 97 | 99 | | |
| EX-527 | Cayman Epigenetic screening library | | | 92 | 98 | 96 | 98 | 87 | 103 | 98 | 104 | 95 | 118 | 103 | 98 | | |
| Scriptaid | Cayman Epigenetic screening library | | | 89 | 103 | 97 | 104 | 79 | 95 | 82 | 94 | 0 | 0 | 1 | 2 | | |
| MS023 | Cayman Epigenetic screening library | | | 111 | 109 | 106 | 102 | 94 | 101 | 90 | 100 | 39 | 59 | 54 | 47 | | |
| JGB1741 | Cayman Epigenetic screening library | | | 88 | 100 | 93 | 87 | 86 | 93 | 93 | 87 | 89 | 120 | 94 | 85 | | |
| Tenovin-6 | Cayman Epigenetic screening library | | | 90 | 93 | 94 | 84 | 98 | 92 | 100 | 92 | 86 | 117 | 93 | 89 | | |
| M 344 | Cayman Epigenetic screening library | | | 93 | 91 | 96 | 95 | 81 | 98 | 82 | 93 | 0 | 0 | 2 | 0 | | |
| Selinexor | Cayman Epigenetic screening library | | | 109 | 108 | 103 | 108 | 83 | 119 | 77 | 102 | 1 | 2 | 4 | 2 | | |
| Mirin | Cayman Epigenetic screening library | | | 87 | 91 | 98 | 97 | 86 | 101 | 97 | 96 | 89 | 117 | 99 | 103 | | |
| Chidamide | Cayman Epigenetic screening library | | | 81 | 94 | 100 | 95 | 68 | 98 | 85 | 91 | 1 | 1 | 21 | 8 | | |
| SAHA | Cayman Epigenetic screening library | | | 87 | 95 | 92 | 88 | 79 | 101 | 90 | 87 | 2 | 2 | 15 | 8 | | |
| f-Amidine (trifluoroacetate salt) | Cayman Epigenetic screening library | | | 82 | 95 | 99 | 96 | 83 | 107 | 96 | 98 | 88 | 119 | 91 | 93 | | |
| Suberohydroxamic Acid | Cayman Epigenetic screening library | | | 87 | 101 | 99 | 101 | 82 | 107 | 94 | 96 | 58 | 90 | 75 | 91 | | |
| Apicidin | Cayman Epigenetic screening library | | | 89 | 102 | 102 | 98 | 82 | 107 | 95 | 108 | 51 | 80 | 83 | 86 | | |
| 3-amino Benzamide | Cayman Epigenetic screening library | | | 75 | 97 | 101 | 102 | 83 | 111 | 96 | 102 | 75 | 110 | 90 | 94 | | |
| HC Toxin | Cayman Epigenetic screening library | | | 40 | 77 | 46 | 79 | 0 | 0 | 0 | 0 | 0 | 0 | 0 | 0 | | |
| Phthalazinone pyrazole | Cayman Epigenetic screening library | | | 87 | 101 | 98 | 97 | 69 | 91 | 89 | 90 | 2 | 14 | 46 | 42 | | |
| AGK2 | Cayman Epigenetic screening library | | | 90 | 100 | 95 | 96 | 89 | 106 | 102 | 103 | 94 | 141 | 99 | 116 | | |
| 2,4-Pyridinedicarboxylic Acid | Enzo SCREEN-WELL® epigenetics library | | | 88 | 86 | 102 | 98 | 89 | 83 | 90 | 81 | 90 | 91 | 88 | 93 | | |
| 5-Aza-2'-deoxycytidine (Decitabine) | Enzo SCREEN-WELL® epigenetics library | | | 92 | 91 | 95 | 89 | 48 | 61 | 56 | 72 | 22 | 25 | 24 | 39 | | |
| AGK2 | Enzo SCREEN-WELL® epigenetics library | | | 97 | 93 | 102 | 92 | 102 | 83 | 99 | 87 | 103 | 102 | 90 | 101 | | |
| Aminoresveratrol sulfate | Enzo SCREEN-WELL® epigenetics library | | | 98 | 98 | 104 | 97 | 98 | 100 | 105 | 94 | 98 | 69 | 103 | 88 | | |
| Anacardic acid | Enzo SCREEN-WELL® epigenetics library | | | 104 | 92 | 107 | 93 | 100 | 96 | 103 | 94 | 108 | 89 | 99 | 91 | | |
| Apicidin | Enzo SCREEN-WELL® epigenetics library | | | 93 | 96 | 93 | 92 | 19 | 67 | 38 | 81 | 0 | 0 | 0 | 0 | | |
| B2 | Enzo SCREEN-WELL® epigenetics library | | | 99 | 101 | 106 | 98 | 106 | 100 | 109 | 102 | 95 | 78 | 100 | 97 | | |
| BIX-01294·3HCl | Enzo SCREEN-WELL® epigenetics library | | | 102 | 91 | 98 | 92 | 96 | 85 | 94 | 93 | 92 | 90 | 83 | 81 | | |
| BML-210 | Enzo SCREEN-WELL® epigenetics library | | | 96 | 90 | 98 | 96 | 97 | 93 | 97 | 94 | 74 | 59 | 92 | 86 | | |
| BML-266 | Enzo SCREEN-WELL® epigenetics library | | | 100 | 92 | 102 | 93 | 106 | 90 | 101 | 90 | 115 | 90 | 105 | 90 | | |
| BML-278 | Enzo SCREEN-WELL® epigenetics library | | | 97 | 90 | 102 | 96 | 93 | 94 | 100 | 92 | 90 | 85 | 92 | 88 | | |
| BML-281 | Enzo SCREEN-WELL® epigenetics library | | | 102 | 100 | 106 | 104 | 109 | 95 | 98 | 98 | 105 | 0 | 108 | 0 | | |
| Butyrolactone 3 | Enzo SCREEN-WELL® epigenetics library | | | 100 | 90 | 100 | 90 | 97 | 82 | 96 | 82 | 96 | 70 | 90 | 93 | | |
| CI-994 | Enzo SCREEN-WELL® epigenetics library | | | 97 | 90 | 102 | 97 | 97 | 91 | 97 | 89 | 102 | 88 | 106 | 91 | | |
| CTPB | Enzo SCREEN-WELL® epigenetics library | | | 105 | 90 | 105 | 91 | 100 | 88 | 102 | 88 | 108 | 85 | 103 | 91 | | |
| EX-527 | Enzo SCREEN-WELL® epigenetics library | | | 91 | 101 | 101 | 105 | 108 | 100 | 103 | 97 | 103 | 75 | 106 | 104 | | |
| Fluoro-SAHA | Enzo SCREEN-WELL® epigenetics library | | | 95 | 97 | 101 | 101 | 103 | 100 | 102 | 95 | 23 | 7 | 44 | 18 | | |
| Garcinol | Enzo SCREEN-WELL® epigenetics library | | | 90 | 94 | 99 | 92 | 93 | 91 | 99 | 87 | 96 | 91 | 94 | 102 | | |
| Isonicotinamide | Enzo SCREEN-WELL® epigenetics library | | | 95 | 98 | 97 | 90 | 99 | 93 | 97 | 85 | 87 | 103 | 92 | 89 | | |
| ITSA-1 | Enzo SCREEN-WELL® epigenetics library | | | 92 | 87 | 96 | 89 | 91 | 82 | 96 | 85 | 94 | 91 | 91 | 91 | | |
| M-344 | Enzo SCREEN-WELL® epigenetics library | | | 91 | 88 | 98 | 91 | 71 | 73 | 71 | 74 | 0 | 0 | 0 | 0 | | |
| MC-1293 | Enzo SCREEN-WELL® epigenetics library | | | 103 | 91 | 106 | 95 | 99 | 90 | 100 | 85 | 94 | 90 | 92 | 93 | | |
| NCH-51 | Enzo SCREEN-WELL® epigenetics library | | | 93 | 88 | 98 | 90 | 92 | 84 | 90 | 87 | 93 | 78 | 94 | 92 | | |
| Nicotinamide | Enzo SCREEN-WELL® epigenetics library | | | 96 | 93 | 101 | 91 | 94 | 86 | 102 | 90 | 101 | 97 | 96 | 95 | | |
| NSC-3852 | Enzo SCREEN-WELL® epigenetics library | | | 101 | 94 | 106 | 93 | 105 | 78 | 100 | 83 | 102 | 0 | 102 | 6 | | |
| Oxamflatin | Enzo SCREEN-WELL® epigenetics library | | | 107 | 96 | 108 | 93 | 84 | 92 | 71 | 84 | 0 | 0 | 0 | 0 | | |
| Phenylbutyrate·Na | Enzo SCREEN-WELL® epigenetics library | | | 92 | 88 | 101 | 94 | 95 | 87 | 96 | 92 | 94 | 96 | 94 | 99 | | |
| Piceatannol | Enzo SCREEN-WELL® epigenetics library | | | 105 | 98 | 111 | 98 | 97 | 87 | 106 | 90 | 107 | 87 | 105 | 98 | | |
| Resveratrol | Enzo SCREEN-WELL® epigenetics library | | | 100 | 92 | 99 | 96 | 99 | 90 | 95 | 93 | 100 | 88 | 98 | 97 | | |
| Salermide | Enzo SCREEN-WELL® epigenetics library | | | 97 | 94 | 102 | 90 | 94 | 82 | 100 | 93 | 104 | 93 | 103 | 95 | | |
| Scriptaid | Enzo SCREEN-WELL® epigenetics library | | | 92 | 87 | 96 | 94 | 87 | 78 | 85 | 84 | 0 | 2 | 1 | 3 | | |
| Sirtinol | Enzo SCREEN-WELL® epigenetics library | | | 98 | 92 | 103 | 96 | 98 | 99 | 98 | 91 | 99 | 73 | 97 | 92 | | |
| Splitomicin | Enzo SCREEN-WELL® epigenetics library | | | 97 | 90 | 99 | 93 | 98 | 82 | 103 | 97 | 98 | 82 | 92 | 95 | | |
| Suberoyl bis-hydroxamic acid | Enzo SCREEN-WELL® epigenetics library | | | 93 | 92 | 97 | 95 | 99 | 91 | 88 | 93 | 88 | 76 | 90 | 76 | | |
| Suramin·6Na | Enzo SCREEN-WELL® epigenetics library | | | 100 | 103 | 108 | 100 | 98 | 97 | 109 | 100 | 108 | 59 | 110 | 97 | | |
| Tranylcypromine hemisulfate | Enzo SCREEN-WELL® epigenetics library | | | 95 | 91 | 102 | 95 | 95 | 97 | 98 | 89 | 97 | 88 | 96 | 90 | | |
| Triacetylresveratrol | Enzo SCREEN-WELL® epigenetics library | | | 93 | 96 | 103 | 89 | 101 | 91 | 98 | 92 | 86 | 73 | 95 | 93 | | |
| Trichostatin A | Enzo SCREEN-WELL® epigenetics library | | | 91 | 93 | 96 | 95 | 62 | 78 | 76 | 90 | 0 | 0 | 0 | 0 | | |
| Valproic acid | Enzo SCREEN-WELL® epigenetics library | | | 101 | 87 | 104 | 103 | 103 | 93 | 99 | 88 | 103 | 94 | 99 | 100 | | |

| | | | | | | | | | | | | | | | | | |
|---|---|---|---|---|---|---|---|---|---|---|---|---|---|---|---|---|---|
| Valproic acid hydroxamate | Enzo SCREEN-WELL® epigenetics library | | | 104 | 96 | 96 | 91 | 95 | 90 | 92 | 95 | 100 | 85 | 95 | 89 | | |
| Vorinostat (SAHA) | Enzo SCREEN-WELL® epigenetics library | | | 94 | 101 | 102 | 99 | 93 | 101 | 88 | 94 | 0 | 1 | 1 | 2 | | |
| Hesperadin | MCE Cell Cycle/DNA Damage Compound Library | 89 | 95 | 87 | | 93 | | 0 | | 3 | | 0 | | 0 | | -4 | 4 |
| AZD1152 | MCE Cell Cycle/DNA Damage Compound Library | 99 | 105 | 94 | | 100 | | 97 | | 98 | | 60 | | 92 | | -3 | 1 |
| SJB2-043 | MCE Cell Cycle/DNA Damage Compound Library | 124 | 120 | 117 | | 110 | | 90 | | 96 | | -1 | | 8 | | -3 | 0 |
| PFI-1 | MCE Cell Cycle/DNA Damage Compound Library | 94 | 91 | 94 | | 89 | | 91 | | 91 | | 70 | | 77 | | -3 | 0 |
| MLN8054 | MCE Cell Cycle/DNA Damage Compound Library | 104 | 103 | 99 | | 105 | | 73 | | 79 | | 10 | | 10 | | -3 | 2 |
| Triapine | MCE Cell Cycle/DNA Damage Compound Library | 94 | 92 | 98 | | 94 | | 91 | | 89 | | 0 | | 16 | | -3 | 0 |
| Mocetinostat | MCE Cell Cycle/DNA Damage Compound Library | 109 | 102 | 106 | | 103 | | 52 | | 46 | | 0 | | 0 | | -3 | 0 |
| ZM-447439 | MCE Cell Cycle/DNA Damage Compound Library | 96 | 99 | 98 | | 97 | | 91 | | 95 | | 49 | | 85 | | -3 | 1 |
| BIX-01294 | MCE Cell Cycle/DNA Damage Compound Library | 103 | 113 | 103 | | 107 | | 97 | | 107 | | 93 | | 102 | | -2 | 0 |
| AMG 900 | MCE Cell Cycle/DNA Damage Compound Library | 98 | 99 | 0 | | 1 | | 1 | | 1 | | 1 | | 1 | | -2 | 2 |
| TG-101348 | MCE Cell Cycle/DNA Damage Compound Library | 99 | 89 | 100 | | 96 | | 87 | | 85 | | 15 | | 67 | | -2 | 0 |
| Etoposide | MCE Cell Cycle/DNA Damage Compound Library | 88 | 96 | 89 | | 91 | | 49 | | 80 | | 2 | | 23 | | -2 | 0 |
| CCT241533 (hydrochloride) | MCE Cell Cycle/DNA Damage Compound Library | 112 | 113 | 112 | | 107 | | 54 | | 103 | | 32 | | 86 | | -2 | 0 |
| CI-994 | MCE Cell Cycle/DNA Damage Compound Library | 101 | 95 | 98 | | 98 | | 101 | | 95 | | 65 | | 63 | | -1 | 0 |
| Fosbretabulin (disodium) | MCE Cell Cycle/DNA Damage Compound Library | 92 | 90 | 88 | | 90 | | 95 | | 84 | | 1 | | 0 | | -1 | 0 |
| GSK-J4 | MCE Cell Cycle/DNA Damage Compound Library | 90 | 99 | 96 | | 108 | | 89 | | 94 | | 100 | | 88 | | -1 | 2 |
| NMS-873 | MCE Cell Cycle/DNA Damage Compound Library | 108 | 107 | 102 | | 104 | | 85 | | 83 | | 64 | | 64 | | -1 | -1 |
| Dacinostat | MCE Cell Cycle/DNA Damage Compound Library | 87 | 101 | 77 | | 81 | | 1 | | 3 | | -1 | | 1 | | -1 | 0 |
| Ro3280 | MCE Cell Cycle/DNA Damage Compound Library | 89 | 97 | 92 | | 85 | | 0 | | 31 | | 0 | | 1 | | -1 | 1 |
| SRT 1720 (Hydrochloride) | MCE Cell Cycle/DNA Damage Compound Library | 93 | 89 | 88 | | 95 | | 96 | | 90 | | 87 | | 84 | | -1 | 0 |
| UNC1999 | MCE Cell Cycle/DNA Damage Compound Library | 103 | 98 | 103 | | 97 | | 103 | | 102 | | 102 | | 108 | | -1 | 12 |
| Vorinostat | MCE Cell Cycle/DNA Damage Compound Library | 109 | 110 | 107 | | 111 | | 104 | | 104 | | 71 | | 65 | | -1 | -1 |
| CHR-6494 | MCE Cell Cycle/DNA Damage Compound Library | 100 | 100 | 94 | | 90 | | 49 | | 69 | | 25 | | 41 | | -1 | 0 |
| Mitomycin C | MCE Cell Cycle/DNA Damage Compound Library | 100 | 100 | 83 | | 70 | | 5 | | 3 | | 0 | | 0 | | -1 | 0 |
| AT7519 (trifluoroacetate) | MCE Cell Cycle/DNA Damage Compound Library | 86 | 92 | 102 | | 97 | | 103 | | 94 | | 2 | | 84 | | -1 | 0 |
| DBeQ | MCE Cell Cycle/DNA Damage Compound Library | 93 | 93 | 98 | | 91 | | 95 | | 84 | | 98 | | 104 | | -1 | -1 |
| HMN-214 | MCE Cell Cycle/DNA Damage Compound Library | 90 | 88 | 89 | | 87 | | 66 | | 72 | | 1 | | 1 | | -1 | 0 |
| Alisertib | MCE Cell Cycle/DNA Damage Compound Library | 88 | 87 | 81 | | 83 | | 21 | | 20 | | 1 | | 1 | | -1 | 3 |
| AT9283 | MCE Cell Cycle/DNA Damage Compound Library | 101 | 97 | 91 | | 98 | | 1 | | 10 | | 0 | | 5 | | -1 | 0 |
| Flavopiridol (Hydrochloride) | MCE Cell Cycle/DNA Damage Compound Library | 104 | 102 | 105 | | 97 | | 75 | | 81 | | 4 | | 0 | | -1 | 0 |
| M344 | MCE Cell Cycle/DNA Damage Compound Library | 91 | 92 | 87 | | 85 | | 75 | | 71 | | 0 | | 0 | | -1 | 0 |
| Ro-3306 | MCE Cell Cycle/DNA Damage Compound Library | 95 | 93 | 94 | | 94 | | 90 | | 96 | | 92 | | 88 | | -1 | 3 |
| GSK-1070916 | MCE Cell Cycle/DNA Damage Compound Library | 101 | 96 | 100 | | 103 | | 1 | | 79 | | -1 | | 1 | | 0 | 1 |
| PIK-75 | MCE Cell Cycle/DNA Damage Compound Library | 109 | 104 | 101 | | 96 | | 23 | | 40 | | 1 | | 0 | | 0 | 1 |
| 4SC-202 (free base) | MCE Cell Cycle/DNA Damage Compound Library | 99 | 100 | 93 | | 98 | | 91 | | 88 | | 0 | | 1 | | 0 | 0 |
| Bromosporine | MCE Cell Cycle/DNA Damage Compound Library | 85 | 90 | 94 | | 87 | | 83 | | 76 | | 17 | | 32 | | 0 | 0 |
| Aurora A inhibitor | MCE Cell Cycle/DNA Damage Compound Library | 93 | 94 | 87 | | 92 | | 91 | | 90 | | 32 | | 42 | | 0 | 0 |
| Epothilone D | MCE Cell Cycle/DNA Damage Compound Library | 86 | 96 | 80 | | 95 | | 0 | | 0 | | 1 | | 0 | | 0 | 0 |
| Lexibulin | MCE Cell Cycle/DNA Damage Compound Library | 111 | 102 | 105 | | 102 | | 0 | | 10 | | 0 | | 0 | | 0 | 0 |
| Pimelic Diphenylamide 106 (analog) | MCE Cell Cycle/DNA Damage Compound Library | 94 | 101 | 93 | | 106 | | 94 | | 97 | | 96 | | 96 | | 0 | 0 |
| Entinostat | MCE Cell Cycle/DNA Damage Compound Library | 107 | 107 | 104 | | 104 | | 62 | | 60 | | 0 | | 1 | | 0 | 0 |
| RGFP966 | MCE Cell Cycle/DNA Damage Compound Library | 83 | 91 | 97 | | 91 | | 80 | | 100 | | 2 | | 9 | | 0 | -1 |
| ACY-1215 | MCE Cell Cycle/DNA Damage Compound Library | 105 | 101 | 99 | | 100 | | 101 | | 106 | | 101 | | 87 | | 0 | -1 |
| TAK-901 | MCE Cell Cycle/DNA Damage Compound Library | 89 | 93 | 87 | | 93 | | 10 | | 91 | | 0 | | 42 | | 0 | 1 |
| Pracinostat | MCE Cell Cycle/DNA Damage Compound Library | 97 | 93 | 88 | | 92 | | 50 | | 49 | | 0 | | 0 | | 0 | -1 |
| D-64131 | MCE Cell Cycle/DNA Damage Compound Library | 97 | 103 | 101 | | 99 | | 27 | | 28 | | 1 | | 0 | | 0 | 0 |
| THZ1 (Hydrochloride) | MCE Cell Cycle/DNA Damage Compound Library | 90 | 93 | 87 | | 88 | | 14 | | 73 | | 0 | | 2 | | 0 | 0 |
| AZD1152-HQPA | MCE Cell Cycle/DNA Damage Compound Library | 89 | 89 | 89 | | 95 | | 13 | | 90 | | 7 | | 62 | | 0 | 1 |
| Nocodazole | MCE Cell Cycle/DNA Damage Compound Library | 90 | 93 | 88 | | 90 | | 1 | | 1 | | 0 | | 0 | | 0 | 0 |
| ENMD-2076 (Tartrate) | MCE Cell Cycle/DNA Damage Compound Library | 101 | 99 | 89 | | 97 | | 49 | | 72 | | 5 | | 3 | | 0 | 0 |
| b-AP15 | MCE Cell Cycle/DNA Damage Compound Library | 116 | 113 | 110 | | 119 | | 96 | | 102 | | 0 | | 15 | | 0 | -1 |
| β-Lapachone | MCE Cell Cycle/DNA Damage Compound Library | 94 | 96 | 87 | | 89 | | 96 | | 97 | | 93 | | 99 | | 0 | 0 |
| Topotecan (Hydrochloride) | MCE Cell Cycle/DNA Damage Compound Library | 91 | 98 | 75 | | 85 | | 0 | | 1 | | 0 | | 0 | | 0 | -1 |
| OTX-015 | MCE Cell Cycle/DNA Damage Compound Library | 104 | 100 | 104 | | 97 | | 56 | | 75 | | 1 | | 6 | | 0 | 0 |
| XL228 | MCE Cell Cycle/DNA Damage Compound Library | 90 | 97 | 77 | | 94 | | 1 | | 3 | | 0 | | 0 | | 0 | -1 |
| Flavopiridol | MCE Cell Cycle/DNA Damage Compound Library | 106 | 109 | 104 | | 107 | | 113 | | 89 | | 6 | | 1 | | 0 | 0 |
| RG2833 | MCE Cell Cycle/DNA Damage Compound Library | 106 | 97 | 104 | | 101 | | 107 | | 108 | | 64 | | 67 | | 0 | 0 |
| CP-466722 | MCE Cell Cycle/DNA Damage Compound Library | 100 | 103 | 101 | | 110 | | 96 | | 97 | | 65 | | 76 | | 0 | 1 |
| ENMD-2076 | MCE Cell Cycle/DNA Damage Compound Library | 111 | 106 | 109 | | 108 | | 71 | | 93 | | 25 | | 52 | | 0 | 1 |
| Epirubicin (hydrochloride) | MCE Cell Cycle/DNA Damage Compound Library | 98 | 94 | 70 | | 97 | | 1 | | 45 | | 1 | | 1 | | 0 | 0 |
| Gemcitabine | MCE Cell Cycle/DNA Damage Compound Library | 84 | 82 | 1 | | 0 | | 0 | | 0 | | 1 | | 0 | | 0 | -1 |
| Panobinostat | MCE Cell Cycle/DNA Damage Compound Library | 5 | 54 | 0 | | 4 | | 0 | | 0 | | 1 | | 0 | | 0 | 0 |
| SB-743921 | MCE Cell Cycle/DNA Damage Compound Library | 109 | 108 | 0 | | 29 | | 0 | | 0 | | 0 | | 0 | | 0 | 0 |
| ELR510444 | MCE Cell Cycle/DNA Damage Compound Library | 91 | 92 | 69 | | 66 | | 1 | | -1 | | 0 | | 0 | | 0 | -1 |
| Melphalan | MCE Cell Cycle/DNA Damage Compound Library | 94 | 91 | 93 | | 88 | | 92 | | 93 | | 62 | | 65 | | 0 | 0 |
| CX-5461 | MCE Cell Cycle/DNA Damage Compound Library | 105 | 105 | 101 | | 105 | | 93 | | 100 | | 35 | | 61 | | 1 | 0 |
| Trichostatin A | MCE Cell Cycle/DNA Damage Compound Library | 99 | 99 | 101 | | 102 | | 102 | | 105 | | 60 | | 79 | | 1 | 0 |
| SU9516 | MCE Cell Cycle/DNA Damage Compound Library | 113 | 103 | 106 | | 107 | | 109 | | 105 | | 76 | | 93 | | 1 | 0 |
| ARRY-520 (R enantiomer) | MCE Cell Cycle/DNA Damage Compound Library | 96 | 94 | 1 | | 34 | | 0 | | 1 | | 2 | | 1 | | 1 | 0 |
| WAY-262611 | MCE Cell Cycle/DNA Damage Compound Library | 106 | 102 | 101 | | 109 | | 101 | | 99 | | 37 | | 40 | | 1 | 0 |
| PHA-767491 (hydrochloride) | MCE Cell Cycle/DNA Damage Compound Library | 103 | 100 | 98 | | 100 | | 108 | | 97 | | 86 | | 76 | | 1 | 0 |
| SNS-314 | MCE Cell Cycle/DNA Damage Compound Library | 101 | 99 | 93 | | 92 | | -1 | | 0 | | 0 | | 1 | | 1 | 2 |
| Vinblastine (sulfate) | MCE Cell Cycle/DNA Damage Compound Library | 78 | 93 | 1 | | 10 | | 1 | | 1 | | 0 | | 0 | | 1 | 0 |
| Gemcitabine (elaidate) | MCE Cell Cycle/DNA Damage Compound Library | 64 | 85 | 1 | | 1 | | 0 | | 0 | | -1 | | 0 | | 1 | 0 |
| FRAX486 | MCE Cell Cycle/DNA Damage Compound Library | 110 | 112 | 109 | | 109 | | 108 | | 101 | | 70 | | 85 | | 1 | 0 |
| UNC 0631 | MCE Cell Cycle/DNA Damage Compound Library | 84 | 92 | 78 | | 82 | | 87 | | 92 | | 78 | | 88 | | 1 | 0 |
| SP2509 | MCE Cell Cycle/DNA Damage Compound Library | 102 | 100 | 98 | | 103 | | 73 | | 82 | | -1 | | 1 | | 1 | 1 |
| Tozasertib | MCE Cell Cycle/DNA Damage Compound Library | 90 | 95 | 85 | | 91 | | 1 | | 63 | | 0 | | 1 | | 1 | 2 |
| Pirarubicin (Hydrochloride) | MCE Cell Cycle/DNA Damage Compound Library | 104 | 97 | 96 | | 99 | | 14 | | 60 | | 0 | | 1 | | 1 | 0 |
| SN-38 | MCE Cell Cycle/DNA Damage Compound Library | 88 | 86 | 0 | | 10 | | 0 | | 0 | | 0 | | 0 | | 1 | -1 |
| Clofarabine | MCE Cell Cycle/DNA Damage Compound Library | 108 | 103 | 59 | | 29 | | -1 | | 0 | | 0 | | 0 | | 1 | -1 |
| Rigosertib (sodium) | MCE Cell Cycle/DNA Damage Compound Library | 89 | 87 | 95 | | 86 | | 78 | | 80 | | 1 | | 0 | | 1 | 1 |
| Belinostat | MCE Cell Cycle/DNA Damage Compound Library | 102 | 106 | 99 | | 101 | | 74 | | 78 | | 1 | | 0 | | 1 | 1 |
| Resminostat (hydrochloride) | MCE Cell Cycle/DNA Damage Compound Library | 100 | 100 | 101 | | 102 | | 97 | | 101 | | 2 | | 1 | | 1 | 0 |
| PR-619 | MCE Cell Cycle/DNA Damage Compound Library | 98 | 90 | 94 | | 87 | | 94 | | 85 | | 97 | | 97 | | 1 | 1 |
| PF-03814735 | MCE Cell Cycle/DNA Damage Compound Library | 91 | 95 | 87 | | 92 | | 11 | | 20 | | 1 | | 0 | | 1 | 0 |
| CDK-IN-2 | MCE Cell Cycle/DNA Damage Compound Library | 92 | 98 | 97 | | 98 | | 83 | | 92 | | 2 | | -1 | | 1 | -1 |
| UNC0379 | MCE Cell Cycle/DNA Damage Compound Library | 97 | 103 | 102 | | 103 | | 103 | | 99 | | 88 | | 100 | | 1 | 30 |
| JIB-04 | MCE Cell Cycle/DNA Damage Compound Library | 95 | 100 | 97 | | 105 | | 102 | | 103 | | 82 | | 83 | | 1 | 1 |
| Scriptaid | MCE Cell Cycle/DNA Damage Compound Library | 89 | 91 | 97 | | 84 | | 56 | | 48 | | 0 | | -1 | | 1 | 0 |
| Triciribine | MCE Cell Cycle/DNA Damage Compound Library | 103 | 102 | 103 | | 101 | | 73 | | 57 | | 5 | | 4 | | 1 | 1 |
| Daunorubicin (Hydrochloride) | MCE Cell Cycle/DNA Damage Compound Library | 90 | 102 | 6 | | 71 | | 0 | | 3 | | 0 | | 1 | | 1 | 0 |
| Dinaciclib | MCE Cell Cycle/DNA Damage Compound Library | 106 | 99 | 98 | | 94 | | -1 | | 1 | | 1 | | 0 | | 1 | 0 |
| Droxinostat | MCE Cell Cycle/DNA Damage Compound Library | 95 | 93 | 93 | | 99 | | 90 | | 92 | | 66 | | 79 | | 1 | 20 |
| Teniposide | MCE Cell Cycle/DNA Damage Compound Library | 84 | 86 | 26 | | 69 | | 0 | | 9 | | 0 | | 1 | | 1 | -1 |
| 6-Thioguanine | MCE Cell Cycle/DNA Damage Compound Library | 109 | 115 | 108 | | 111 | | 98 | | 102 | | 6 | | 9 | | 1 | 4 |
| Danusertib | MCE Cell Cycle/DNA Damage Compound Library | 104 | 102 | 105 | | 103 | | 59 | | 95 | | 1 | | 44 | | 1 | 3 |
| Pralatrexate | MCE Cell Cycle/DNA Damage Compound Library | 99 | 100 | 1 | | 1 | | 1 | | 2 | | 1 | | 0 | | 2 | 1 |
| AZ20 | MCE Cell Cycle/DNA Damage Compound Library | 103 | 96 | 103 | | 97 | | 60 | | 91 | | 0 | | 5 | | 2 | 2 |
| Mitoxantrone (dihydrochloride) | MCE Cell Cycle/DNA Damage Compound Library | 26 | 68 | 5 | | 35 | | 0 | | 1 | | 1 | | 0 | | 2 | 1 |
| Decitabine | MCE Cell Cycle/DNA Damage Compound Library | 103 | 100 | 90 | | 96 | | 42 | | 65 | | 8 | | 18 | | 2 | 5 |
| ETP-46464 | MCE Cell Cycle/DNA Damage Compound Library | 106 | 104 | 107 | | 110 | | 94 | | 102 | | 29 | | 64 | | 2 | 4 |
| Purvalanol A | MCE Cell Cycle/DNA Damage Compound Library | 118 | 104 | 112 | | 104 | | 107 | | 102 | | 100 | | 97 | | 3 | 6 |
| Amsacrine | MCE Cell Cycle/DNA Damage Compound Library | 92 | 89 | 76 | | 78 | | 4 | | 22 | | 0 | | 1 | | 3 | 2 |
| BIO | MCE Cell Cycle/DNA Damage Compound Library | 88 | 90 | 85 | | 82 | | 88 | | 88 | | 55 | | 82 | | 3 | 2 |
| NVP-HSP990 | MCE Cell Cycle/DNA Damage Compound Library | 102 | 105 | 82 | | 93 | | 4 | | 14 | | 3 | | 8 | | 3 | 9 |
| LDC000067 | MCE Cell Cycle/DNA Damage Compound Library | 96 | 104 | 92 | | 98 | | 99 | | 106 | | 103 | | 100 | | 4 | 18 |
| Treosulfan | MCE Cell Cycle/DNA Damage Compound Library | 90 | 93 | 92 | | 90 | | 88 | | 92 | | 88 | | 88 | | 5 | 14 |
| AMG 925 | MCE Cell Cycle/DNA Damage Compound Library | 104 | 99 | 100 | | 103 | | 92 | | 101 | | 61 | | 84 | | 5 | 10 |
| 5-Azacytidine | MCE Cell Cycle/DNA Damage Compound Library | 103 | 106 | 100 | | 99 | | 97 | | 99 | | 57 | | 87 | | 10 | 9 |
| Palbociclib (hydrochloride) | MCE Cell Cycle/DNA Damage Compound Library | 93 | 96 | 88 | | 87 | | 81 | | 85 | | 25 | | 50 | | 10 | 15 |
| ML324 | MCE Cell Cycle/DNA Damage Compound Library | 91 | 100 | 97 | | 98 | | 97 | | 101 | | 92 | | 98 | | 10 | 17 |
| Mps1-IN-1 | MCE Cell Cycle/DNA Damage Compound Library | 93 | 90 | 84 | | 89 | | 95 | | 90 | | 90 | | 97 | | 10 | 7 |
| LEE011 (succinate) | MCE Cell Cycle/DNA Damage Compound Library | 94 | 90 | 87 | | 90 | | 89 | | 98 | | 65 | | 79 | | 12 | 27 |
| Dacarbazine | MCE Cell Cycle/DNA Damage Compound Library | 113 | 106 | 108 | | 106 | | 112 | | 104 | | 101 | | 74 | | 13 | 9 |
| LEE011 | MCE Cell Cycle/DNA Damage Compound Library | 100 | 103 | 100 | | 107 | | 100 | | 103 | | 87 | | 94 | | 16 | 38 |
| Tubastatin A (Hydrochloride) | MCE Cell Cycle/DNA Damage Compound Library | 106 | 99 | 97 | | 102 | | 100 | | 105 | | 104 | | 96 | | 16 | 23 |
| RVX-208 | MCE Cell Cycle/DNA Damage Compound Library | 101 | 96 | 104 | | 98 | | 105 | | 97 | | 95 | | 97 | | 17 | 39 |
| AZD-2461 | MCE Cell Cycle/DNA Damage Compound Library | 100 | 96 | 109 | | 102 | | 105 | | 105 | | 89 | | 94 | | 18 | 62 |
| BG45 | MCE Cell Cycle/DNA Damage Compound Library | 108 | 103 | 105 | | 102 | | 107 | | 100 | | 103 | | 95 | | 18 | 22 |
| XL413 (hydrochloride) | MCE Cell Cycle/DNA Damage Compound Library | 95 | 100 | 94 | | 97 | | 95 | | 93 | | 90 | | 89 | | 22 | 62 |
| Inauhzin | MCE Cell Cycle/DNA Damage Compound Library | 100 | 104 | 103 | | 98 | | 107 | | 103 | | 77 | | 84 | | 24 | 36 |
| CVT-313 | MCE Cell Cycle/DNA Damage Compound Library | 85 | 94 | 94 | | 94 | | 91 | | 94 | | 99 | | 98 | | 30 | 18 |
| Poloxin | MCE Cell Cycle/DNA Damage Compound Library | 95 | 90 | 95 | | 90 | | 95 | | 92 | | 90 | | 93 | | 30 | 80 |
| 5 P 22077 | MCE Cell Cycle/DNA Damage Compound Library | 87 | 91 | 88 | | 90 | | 95 | | 88 | | 86 | | 99 | | 32 | 57 |
| GSK2656157 | MCE Cell Cycle/DNA Damage Compound Library | 100 | 102 | 95 | | 91 | | 93 | | 100 | | 92 | | 95 | | 32 | 42 |
| PRT4165 | MCE Cell Cycle/DNA Damage Compound Library | 100 | 94 | 106 | | 103 | | 96 | | 100 | | 104 | | 101 | | 33 | 72 |
| RKI-1447 | MCE Cell Cycle/DNA Damage Compound Library | 102 | 99 | 98 | | 107 | | 106 | | 96 | | 70 | | 86 | | 36 | 59 |
| SR-3677 | MCE Cell Cycle/DNA Damage Compound Library | 94 | 91 | 95 | | 89 | | 90 | | 87 | | 89 | | 85 | | 39 | 50 |

| | | | | | | | | | | | |
|---|---|---|---|---|---|---|---|---|---|---|---|
| Carmofur | MCE Cell Cycle/DNA Damage Compound Library | 83 | 95 | 84 | 90 | 92 | 95 | 78 | 77 | 40 | 44 |
| SLx-2119 | MCE Cell Cycle/DNA Damage Compound Library | 109 | 110 | 104 | 110 | 105 | 102 | 103 | 96 | 44 | 49 |
| Parthenolide | MCE Cell Cycle/DNA Damage Compound Library | 108 | 107 | 100 | 108 | 106 | 103 | 102 | 102 | 46 | 59 |
| GSK343 | MCE Cell Cycle/DNA Damage Compound Library | 89 | 95 | 90 | 98 | 92 | 98 | 92 | 96 | 47 | 75 |
| Rucaparib (phosphate) | MCE Cell Cycle/DNA Damage Compound Library | 96 | 97 | 106 | 103 | 99 | 104 | 95 | 113 | 48 | 80 |
| Triclabendazole | MCE Cell Cycle/DNA Damage Compound Library | 102 | 98 | 102 | 100 | 102 | 108 | 95 | 91 | 51 | 62 |
| T0070907 | MCE Cell Cycle/DNA Damage Compound Library | 98 | 101 | 101 | 103 | 97 | 97 | 99 | 94 | 51 | 54 |
| 5-Fluorouracil | MCE Cell Cycle/DNA Damage Compound Library | 117 | 118 | 111 | 108 | 111 | 109 | 102 | 93 | 54 | 50 |
| A-966492 | MCE Cell Cycle/DNA Damage Compound Library | 93 | 88 | 88 | 89 | 93 | 88 | 92 | 99 | 55 | 81 |
| Tubastatin-A | MCE Cell Cycle/DNA Damage Compound Library | 104 | 98 | 101 | 103 | 101 | 94 | 105 | 100 | 64 | 71 |
| BML-277 | MCE Cell Cycle/DNA Damage Compound Library | 103 | 100 | 102 | 98 | 107 | 105 | 99 | 99 | 65 | 72 |
| Balaglitazone | MCE Cell Cycle/DNA Damage Compound Library | 91 | 93 | 87 | 88 | 88 | 86 | 77 | 83 | 65 | 69 |
| IPA-3 | MCE Cell Cycle/DNA Damage Compound Library | 89 | 87 | 86 | 87 | 90 | 97 | 98 | 91 | 66 | 68 |
| Amodiaquin (dihydrochloride dihydrat | MCE Cell Cycle/DNA Damage Compound Library | 82 | 91 | 87 | 95 | 93 | 95 | 87 | 89 | 68 | 71 |
| Remodelin (hydrobromide) | MCE Cell Cycle/DNA Damage Compound Library | 87 | 90 | 93 | 90 | 91 | 91 | 92 | 102 | 69 | 73 |
| ISRIB (trans-isomer) | MCE Cell Cycle/DNA Damage Compound Library | 91 | 89 | 91 | 92 | 90 | 94 | 84 | 79 | 70 | 77 |
| Miriplatin | MCE Cell Cycle/DNA Damage Compound Library | 89 | 99 | 94 | 96 | 92 | 88 | 78 | 81 | 70 | 71 |
| SGC0946 | MCE Cell Cycle/DNA Damage Compound Library | 86 | 99 | 91 | 95 | 85 | 94 | 74 | 87 | 72 | 79 |
| Veliparib (dihydrochloride) | MCE Cell Cycle/DNA Damage Compound Library | 87 | 92 | 83 | 90 | 89 | 101 | 87 | 99 | 78 | 87 |
| NU 7026 | MCE Cell Cycle/DNA Damage Compound Library | 109 | 101 | 101 | 101 | 105 | 103 | 101 | 101 | 78 | 92 |
| DMAT | MCE Cell Cycle/DNA Damage Compound Library | 111 | 103 | 108 | 104 | 107 | 104 | 110 | 104 | 81 | 93 |
| Nelarabine | MCE Cell Cycle/DNA Damage Compound Library | 95 | 91 | 90 | 92 | 100 | 92 | 97 | 94 | 83 | 89 |
| GW9662 | MCE Cell Cycle/DNA Damage Compound Library | 99 | 102 | 99 | 100 | 102 | 105 | 104 | 98 | 87 | 73 |
| LDN-57444 | MCE Cell Cycle/DNA Damage Compound Library | 117 | 107 | 108 | 110 | 117 | 104 | 118 | 112 | 104 | 101 |
| Troglitazone | MCE Cell Cycle/DNA Damage Compound Library | 99 | 102 | 94 | 102 | 104 | 99 | 92 | 100 | 81 | 85 |
| Ellagic acid | MCE Cell Cycle/DNA Damage Compound Library | 87 | 95 | 94 | 86 | 89 | 93 | 89 | 93 | 81 | 90 |
| TG003 | MCE Cell Cycle/DNA Damage Compound Library | 89 | 91 | 90 | 90 | 93 | 93 | 90 | 92 | 82 | 87 |
| Fasudil (Hydrochloride) | MCE Cell Cycle/DNA Damage Compound Library | 99 | 105 | 104 | 107 | 106 | 107 | 101 | 101 | 83 | 85 |
| AMI-1 | MCE Cell Cycle/DNA Damage Compound Library | 94 | 96 | 91 | 97 | 97 | 96 | 94 | 92 | 85 | 100 |
| G007-LK | MCE Cell Cycle/DNA Damage Compound Library | 92 | 86 | 88 | 86 | 91 | 93 | 83 | 94 | 87 | 97 |
| Teprenone | MCE Cell Cycle/DNA Damage Compound Library | 88 | 94 | 86 | 98 | 89 | 98 | 85 | 99 | 87 | 90 |
| Oxolinic acid | MCE Cell Cycle/DNA Damage Compound Library | 101 | 109 | 102 | 106 | 101 | 115 | 102 | 100 | 88 | 95 |
| Sodium phenylbutyrate | MCE Cell Cycle/DNA Damage Compound Library | 92 | 92 | 81 | 94 | 94 | 93 | 88 | 96 | 90 | 95 |
| Folinic acid (calcium salt pentahydrat | MCE Cell Cycle/DNA Damage Compound Library | 95 | 88 | 91 | 94 | 102 | 97 | 98 | 99 | 92 | 99 |
| SBE13 (Hydrochloride) | MCE Cell Cycle/DNA Damage Compound Library | 100 | 106 | 98 | 100 | 101 | 95 | 96 | 102 | 93 | 97 |
| Altretamine | MCE Cell Cycle/DNA Damage Compound Library | 87 | 95 | 87 | 90 | 92 | 94 | 92 | 99 | 94 | 92 |
| Ciprofibrate | MCE Cell Cycle/DNA Damage Compound Library | 83 | 88 | 88 | 91 | 89 | 100 | 95 | 95 | 94 | 94 |
| Levomefolate (calcium) | MCE Cell Cycle/DNA Damage Compound Library | 92 | 90 | 89 | 96 | 95 | 96 | 94 | 89 | 94 | 96 |
| L-165041 | MCE Cell Cycle/DNA Damage Compound Library | 95 | 98 | 94 | 93 | 98 | 94 | 93 | 97 | 94 | 104 |
| PJ34 (hydrochloride) | MCE Cell Cycle/DNA Damage Compound Library | 108 | 99 | 104 | 94 | 107 | 99 | 105 | 107 | 94 | 103 |
| JW 55 | MCE Cell Cycle/DNA Damage Compound Library | 96 | 104 | 106 | 98 | 101 | 103 | 98 | 104 | 96 | 104 |
| Rosiglitazone (maleate) | MCE Cell Cycle/DNA Damage Compound Library | 94 | 92 | 92 | 96 | 97 | 93 | 96 | 96 | 96 | 88 |
| Purvalanol B | MCE Cell Cycle/DNA Damage Compound Library | 94 | 111 | 104 | 103 | 98 | 102 | 102 | 103 | 98 | 99 |
| SCR7 | MCE Cell Cycle/DNA Damage Compound Library | 103 | 104 | 106 | 104 | 103 | 103 | 104 | 115 | 99 | 99 |
| CW-069 | MCE Cell Cycle/DNA Damage Compound Library | 104 | 102 | 100 | 105 | 100 | 108 | 103 | 98 | 99 | 108 |
| Orotic acid | MCE Cell Cycle/DNA Damage Compound Library | 104 | 106 | 106 | 118 | 105 | 99 | 100 | 103 | 99 | 98 |
| GW 501516 | MCE Cell Cycle/DNA Damage Compound Library | 90 | 95 | 90 | 91 | 90 | 97 | 96 | 94 | 100 | 96 |
| LFM-A13 | MCE Cell Cycle/DNA Damage Compound Library | 89 | 99 | 100 | 101 | 94 | 98 | 96 | 97 | 101 | 99 |
| Thio-TEPA | MCE Cell Cycle/DNA Damage Compound Library | 106 | 103 | 104 | 102 | 105 | 100 | 105 | 105 | 102 | 97 |
| SRT 2104 | MCE Cell Cycle/DNA Damage Compound Library | 86 | 91 | 85 | 93 | 93 | 97 | 89 | 97 | 102 | 104 |
| Capecitabine | MCE Cell Cycle/DNA Damage Compound Library | 101 | 106 | 107 | 102 | 101 | 98 | 100 | 99 | 102 | 102 |
| Hydroxyfasudil (hydrochloride) | MCE Cell Cycle/DNA Damage Compound Library | 105 | 105 | 107 | 121 | 108 | 102 | 103 | 98 | 102 | 104 |
| Valproic acid (sodium salt) | MCE Cell Cycle/DNA Damage Compound Library | 96 | 96 | 112 | 101 | 107 | 101 | 94 | 96 | 102 | 100 |
| Rosilitazone | MCE Cell Cycle/DNA Damage Compound Library | 103 | 102 | 102 | 102 | 103 | 107 | 102 | 102 | 103 | 97 |
| Levoleucovorin (Calcium) | MCE Cell Cycle/DNA Damage Compound Library | 99 | 100 | 101 | 103 | 109 | 103 | 111 | 106 | 103 | 108 |
| TTP 22 | MCE Cell Cycle/DNA Damage Compound Library | 103 | 102 | 105 | 108 | 104 | 105 | 101 | 102 | 103 | 109 |
| GSK-J1 | MCE Cell Cycle/DNA Damage Compound Library | 112 | 103 | 108 | 104 | 104 | 96 | 107 | 107 | 103 | 93 |
| GSK3787 | MCE Cell Cycle/DNA Damage Compound Library | 106 | 105 | 101 | 105 | 102 | 102 | 99 | 101 | 103 | 106 |
| Clofibrate | MCE Cell Cycle/DNA Damage Compound Library | 99 | 112 | 104 | 105 | 104 | 100 | 107 | 105 | 104 | 106 |
| Ifosfamide | MCE Cell Cycle/DNA Damage Compound Library | 103 | 109 | 99 | 106 | 104 | 101 | 108 | 106 | 104 | 98 |
| GSK-J2 | MCE Cell Cycle/DNA Damage Compound Library | 102 | 113 | 106 | 118 | 106 | 102 | 108 | 108 | 105 | 111 |
| PYZD-4409 | MCE Cell Cycle/DNA Damage Compound Library | 100 | 96 | 99 | 95 | 99 | 99 | 93 | 110 | 105 | 104 |
| PYR-41 | MCE Cell Cycle/DNA Damage Compound Library | 104 | 109 | 114 | 107 | 115 | 98 | 110 | 102 | 108 | 97 |
| Trifluorothymidine | MCE Cell Cycle/DNA Damage Compound Library | 83 | 101 | 93 | 101 | 92 | 87 | 21 | 6 | -6 | -2 |
| Podophyllotoxin | MCE Cell Cycle/DNA Damage Compound Library | 111 | 100 | 97 | 100 | 1 | 1 | 2 | -4 | -4 | 1 |
| BRD4770 | MCE Cell Cycle/DNA Damage Compound Library | 107 | 109 | 105 | 109 | 102 | 115 | 98 | 103 | -3 | 2 |
| PF-3758309 | MCE Cell Cycle/DNA Damage Compound Library | 105 | 105 | 86 | 105 | 25 | 88 | 12 | 31 | -3 | 7 |
| MLN0905 | MCE Cell Cycle/DNA Damage Compound Library | 93 | 95 | 89 | 95 | 4 | 5 | 1 | 1 | -3 | 5 |
| AZD-5438 | MCE Cell Cycle/DNA Damage Compound Library | 114 | 116 | 112 | 116 | 110 | 118 | 13 | 24 | -3 | 1 |
| Amonafide | MCE Cell Cycle/DNA Damage Compound Library | 98 | 111 | 91 | 111 | 107 | 102 | 94 | 93 | -2 | -1 |
| 4'-Demethylepipodophyllotoxin | MCE Cell Cycle/DNA Damage Compound Library | 104 | 108 | 105 | 108 | 105 | 102 | 2 | 1 | -2 | 1 |
| RITA | MCE Cell Cycle/DNA Damage Compound Library | 94 | 99 | 78 | 99 | 7 | -3 | 3 | -3 | -2 | 1 |
| Vincristine (sulfate) | MCE Cell Cycle/DNA Damage Compound Library | 74 | 100 | -2 | 100 | 2 | 7 | 0 | -5 | -2 | -2 |
| GSK 525762A | MCE Cell Cycle/DNA Damage Compound Library | 100 | 108 | 106 | 108 | 89 | 102 | 3 | 17 | -2 | 0 |
| Daun02 | MCE Cell Cycle/DNA Damage Compound Library | 92 | 92 | 88 | 92 | 83 | 90 | 3 | 53 | -1 | 2 |
| (+)-JQ-1 | MCE Cell Cycle/DNA Damage Compound Library | 104 | 95 | 107 | 95 | 80 | 87 | 1 | 4 | -1 | 0 |
| Volasertib | MCE Cell Cycle/DNA Damage Compound Library | 108 | 100 | 106 | 100 | 4 | 0 | 1 | -4 | -1 | 0 |
| AZD6738 | MCE Cell Cycle/DNA Damage Compound Library | 93 | 90 | 102 | 90 | 82 | 101 | 5 | 27 | -1 | 1 |
| WP1130 | MCE Cell Cycle/DNA Damage Compound Library | 102 | 108 | 104 | 108 | 88 | 103 | 103 | 104 | -1 | 2 |
| LMK-235 | MCE Cell Cycle/DNA Damage Compound Library | 109 | 108 | 110 | 108 | 96 | 84 | -2 | 2 | -1 | 2 |
| SB1317 | MCE Cell Cycle/DNA Damage Compound Library | 87 | 91 | 98 | 91 | 94 | 92 | 3 | 2 | -1 | 0 |
| MS436 | MCE Cell Cycle/DNA Damage Compound Library | 93 | 96 | 86 | 96 | 90 | 88 | 87 | 95 | -1 | 9 |
| MK-4827 (tosylate) | MCE Cell Cycle/DNA Damage Compound Library | 100 | 99 | 103 | 99 | 96 | 102 | 82 | 101 | -1 | 28 |
| BMH-21 | MCE Cell Cycle/DNA Damage Compound Library | 92 | 87 | 81 | 87 | 66 | 65 | 14 | 14 | -1 | 1 |
| GSK461364 | MCE Cell Cycle/DNA Damage Compound Library | 81 | 91 | 83 | 91 | 1 | 3 | -1 | 0 | -1 | 3 |
| I-BET151 | MCE Cell Cycle/DNA Damage Compound Library | 81 | 89 | 91 | 89 | 65 | 78 | 2 | 6 | -1 | 1 |
| PCI-24781 | MCE Cell Cycle/DNA Damage Compound Library | 93 | 102 | 89 | 102 | 60 | 60 | 0 | -3 | -1 | 0 |
| Methotrexate | MCE Cell Cycle/DNA Damage Compound Library | 101 | 96 | 98 | 96 | -2 | 1 | 4 | 0 | 0 | 5 |
| SNS-032 | MCE Cell Cycle/DNA Damage Compound Library | 96 | 101 | 107 | 101 | 107 | 105 | 1 | 82 | 0 | 0 |
| Geldanamycin | MCE Cell Cycle/DNA Damage Compound Library | 106 | 104 | 101 | 104 | 2 | 43 | 8 | 6 | 0 | 3 |
| Uramustine | MCE Cell Cycle/DNA Damage Compound Library | 96 | 100 | 86 | 100 | 92 | 91 | 88 | 91 | 0 | 1 |
| Reversine | MCE Cell Cycle/DNA Damage Compound Library | 107 | 103 | 105 | 103 | 89 | 88 | 0 | 0 | 0 | 0 |
| Ispinesib | MCE Cell Cycle/DNA Damage Compound Library | 90 | 88 | 86 | 88 | 3 | -8 | -4 | 2 | 0 | 1 |
| CCT129202 | MCE Cell Cycle/DNA Damage Compound Library | 95 | 96 | 92 | 96 | 92 | 94 | 45 | 62 | 0 | 2 |
| MK-5108 | MCE Cell Cycle/DNA Damage Compound Library | 113 | 107 | 111 | 107 | 91 | 100 | 38 | 39 | 0 | -7 |
| ABT-751 | MCE Cell Cycle/DNA Damage Compound Library | 110 | 96 | 102 | 96 | 96 | 101 | -2 | 1 | 0 | 3 |
| Cabazitaxel | MCE Cell Cycle/DNA Damage Compound Library | 93 | 93 | -2 | 93 | 3 | -4 | 1 | -1 | 1 | -2 |
| ARRY-520 | MCE Cell Cycle/DNA Damage Compound Library | 121 | 103 | 0 | 103 | -2 | -2 | 1 | 4 | 1 | -1 |
| Oxaliplatin | MCE Cell Cycle/DNA Damage Compound Library | 107 | 106 | 105 | 106 | 88 | 76 | 13 | 10 | 1 | 1 |
| TG101209 | MCE Cell Cycle/DNA Damage Compound Library | 105 | 101 | 102 | 101 | 95 | 102 | 15 | 41 | 1 | 1 |
| CHIR-124 | MCE Cell Cycle/DNA Damage Compound Library | 96 | 91 | 91 | 91 | 30 | 71 | 1 | 2 | 1 | -7 |
| PHA-848125 | MCE Cell Cycle/DNA Damage Compound Library | 93 | 85 | 90 | 85 | 62 | 73 | 7 | 10 | 1 | 1 |
| Epothilone B | MCE Cell Cycle/DNA Damage Compound Library | 98 | 94 | 1 | 94 | 1 | -1 | 1 | 3 | 1 | 1 |
| LY2603618 | MCE Cell Cycle/DNA Damage Compound Library | 95 | 94 | 96 | 94 | 83 | 102 | 7 | 21 | 1 | -6 |
| AZD-7762 | MCE Cell Cycle/DNA Damage Compound Library | 52 | 84 | 27 | 84 | 4 | 34 | 2 | -2 | 1 | 0 |
| Campathecin | MCE Cell Cycle/DNA Damage Compound Library | 102 | 104 | 42 | 104 | -5 | -9 | 3 | 2 | 1 | 1 |
| NVP-AUY922 | MCE Cell Cycle/DNA Damage Compound Library | 95 | 90 | 84 | 90 | -4 | 9 | 4 | 7 | 1 | 8 |
| Ganetespib | MCE Cell Cycle/DNA Damage Compound Library | 102 | 102 | 92 | 102 | 7 | 16 | 3 | 8 | 1 | 9 |
| Alvespimycin (hydrochloride) | MCE Cell Cycle/DNA Damage Compound Library | 108 | 98 | 104 | 98 | 27 | 75 | 3 | 17 | 1 | 0 |
| Irinotecan (hydrochloride trihydrate) | MCE Cell Cycle/DNA Damage Compound Library | 104 | 99 | 93 | 99 | 78 | 100 | 1 | 11 | 2 | 1 |
| GSK1324726A | MCE Cell Cycle/DNA Damage Compound Library | 90 | 95 | 87 | 95 | 14 | 23 | -1 | -3 | 2 | -2 |
| CCT 137690 | MCE Cell Cycle/DNA Damage Compound Library | 105 | 102 | 100 | 102 | 89 | 99 | -1 | -3 | 2 | 1 |
| TAK-960 | MCE Cell Cycle/DNA Damage Compound Library | 105 | 96 | 102 | 96 | 1 | 0 | 2 | 0 | 2 | 0 |
| Cytarabine | MCE Cell Cycle/DNA Damage Compound Library | 105 | 104 | 107 | 104 | 19 | 25 | 0 | 2 | 2 | -1 |
| MC1568 | MCE Cell Cycle/DNA Damage Compound Library | 108 | 103 | 101 | 103 | 100 | 108 | 84 | 96 | 2 | 2 |
| APTO-253 | MCE Cell Cycle/DNA Damage Compound Library | 111 | 99 | 104 | 99 | 99 | 97 | 6 | 59 | 2 | 5 |
| Irinotecan (hydrochloride) | MCE Cell Cycle/DNA Damage Compound Library | 122 | 104 | 104 | 104 | 112 | 106 | 54 | 86 | 2 | 5 |
| Vinblastine | MCE Cell Cycle/DNA Damage Compound Library | 115 | 115 | 108 | 115 | 46 | 85 | 3 | 10 | 3 | 2 |
| BIIB021 | MCE Cell Cycle/DNA Damage Compound Library | 110 | 96 | 100 | 96 | 78 | 88 | 8 | 11 | 3 | 1 |
| BI 2536 | MCE Cell Cycle/DNA Damage Compound Library | 95 | 98 | 87 | 98 | 0 | 37 | 2 | 0 | 3 | -3 |
| CB-5083 | MCE Cell Cycle/DNA Damage Compound Library | 94 | 97 | 95 | 97 | 84 | 89 | 1 | 0 | 3 | 1 |
| NMS-1286937 | MCE Cell Cycle/DNA Damage Compound Library | 93 | 92 | 104 | 92 | -1 | -5 | -1 | -2 | 3 | 0 |
| 17-AAG | MCE Cell Cycle/DNA Damage Compound Library | 82 | 90 | 85 | 90 | 84 | 83 | 35 | 48 | 3 | 10 |
| VE-821 | MCE Cell Cycle/DNA Damage Compound Library | 89 | 91 | 95 | 91 | 82 | 95 | 48 | 79 | 3 | 3 |
| Paclitaxel | MCE Cell Cycle/DNA Damage Compound Library | 119 | 106 | 111 | 106 | 0 | 78 | 1 | -4 | 3 | -1 |
| Cevipabulin | MCE Cell Cycle/DNA Damage Compound Library | 100 | 103 | 112 | 103 | 2 | 36 | 2 | 1 | 3 | -6 |
| Pemetrexed (disodium) | MCE Cell Cycle/DNA Damage Compound Library | 118 | 112 | 118 | 112 | 4 | 13 | 1 | 3 | 3 | 4 |
| TH588 | MCE Cell Cycle/DNA Damage Compound Library | 104 | 101 | 105 | 101 | 106 | 114 | 105 | 89 | 3 | 6 |
| Pimelic Diphenylamide 106 | MCE Cell Cycle/DNA Damage Compound Library | 98 | 93 | 100 | 93 | 86 | 98 | 79 | 87 | 3 | -3 |
| Vinflunine (Tartrate) | MCE Cell Cycle/DNA Damage Compound Library | 101 | 89 | 93 | 89 | 28 | 53 | 1 | -3 | 3 | -1 |
| Genz-644282 | MCE Cell Cycle/DNA Damage Compound Library | 85 | 91 | 43 | 91 | 2 | -1 | 1 | -2 | 4 | 0 |

| | | | | | | | | | | | | | | | | | |
|---|---|---|---|---|---|---|---|---|---|---|---|---|---|---|---|---|---|
| 5-BrdU | MCE Cell Cycle/DNA Damage Compound Library | 107 | 112 | 107 | | 112 | | 110 | | 114 | | 50 | | 57 | | 4 | 18 |
| 100 mM Nimustine | MCE Cell Cycle/DNA Damage Compound Library | 99 | 106 | 87 | | 106 | | 95 | | 92 | | 93 | | 56 | | 4 | 1 |
| NVP-LCQ195 | MCE Cell Cycle/DNA Damage Compound Library | 96 | 95 | 101 | | 95 | | 98 | | 91 | | 13 | | 29 | | 4 | 0 |
| CX-4945 | MCE Cell Cycle/DNA Damage Compound Library | 88 | 91 | 93 | | 91 | | 93 | | 96 | | 82 | | 90 | | 4 | 0 |
| Gemcitabine (Hydrochloride) | MCE Cell Cycle/DNA Damage Compound Library | 95 | 95 | 33 | | 95 | | -3 | | -1 | | -1 | | 2 | | 4 | 7 |
| Idarubicin (hydrochloride) | MCE Cell Cycle/DNA Damage Compound Library | 55 | 79 | 1 | | 79 | | 2 | | 0 | | 1 | | 2 | | 4 | -2 |
| THZ1 | MCE Cell Cycle/DNA Damage Compound Library | 96 | 87 | 90 | | 87 | | 21 | | 72 | | -2 | | 15 | | 4 | -2 |
| Pemetrexed (disodium hemipenta hyd | MCE Cell Cycle/DNA Damage Compound Library | 113 | 101 | 109 | | 101 | | 8 | | 15 | | -3 | | -2 | | 4 | 3 |
| AZ3146 | MCE Cell Cycle/DNA Damage Compound Library | 91 | 93 | 94 | | 93 | | 93 | | 96 | | 80 | | 77 | | 4 | 3 |
| Roscovitine | MCE Cell Cycle/DNA Damage Compound Library | 91 | 87 | 97 | | 87 | | 93 | | 97 | | 101 | | 102 | | 4 | 2 |
| VE-822 | MCE Cell Cycle/DNA Damage Compound Library | 113 | 103 | 106 | | 103 | | 64 | | 96 | | -1 | | -4 | | 5 | 0 |
| MK-1775 | MCE Cell Cycle/DNA Damage Compound Library | 112 | 106 | 107 | | 106 | | 69 | | 96 | | 4 | | 2 | | 5 | 1 |
| MK-4827 | MCE Cell Cycle/DNA Damage Compound Library | 90 | 93 | 91 | | 93 | | 96 | | 83 | | 77 | | 89 | | 5 | 23 |
| AT7519 (Hydrochloride) | MCE Cell Cycle/DNA Damage Compound Library | 99 | 95 | 93 | | 95 | | 104 | | 99 | | 67 | | 92 | | 5 | 35 |
| Colchicine | MCE Cell Cycle/DNA Damage Compound Library | 100 | 98 | 97 | | 98 | | 4 | | 1 | | -3 | | -4 | | 5 | -5 |
| 6-Mercaptopurine | MCE Cell Cycle/DNA Damage Compound Library | 109 | 94 | 100 | | 94 | | 91 | | 99 | | 68 | | 53 | | 5 | 6 |
| CX-4945 (sodium salt) | MCE Cell Cycle/DNA Damage Compound Library | 100 | 91 | 98 | | 91 | | 101 | | 103 | | 99 | | 97 | | 5 | 1 |
| PHA-793887 | MCE Cell Cycle/DNA Damage Compound Library | 104 | 104 | 106 | | 104 | | 108 | | 107 | | 49 | | 102 | | 5 | 55 |
| TAS-103 (dihydrochloride) | MCE Cell Cycle/DNA Damage Compound Library | 90 | 86 | 68 | | 86 | | 2 | | 0 | | 0 | | -2 | | 5 | -1 |
| BML-210 | MCE Cell Cycle/DNA Damage Compound Library | 95 | 88 | 94 | | 88 | | 93 | | 96 | | 90 | | 86 | | 6 | 0 |
| Floxuridine | MCE Cell Cycle/DNA Damage Compound Library | 101 | 100 | 41 | | 100 | | 2 | | -1 | | 2 | | -4 | | 6 | 1 |
| JQ-1 (carboxylic acid) | MCE Cell Cycle/DNA Damage Compound Library | 112 | 99 | 103 | | 99 | | 107 | | 96 | | 86 | | 89 | | 6 | 13 |
| Doxorubicin (hydrochloride) | MCE Cell Cycle/DNA Damage Compound Library | 104 | 101 | 88 | | 101 | | 8 | | 58 | | 1 | | 0 | | 6 | 1 |
| GW843682X | MCE Cell Cycle/DNA Damage Compound Library | 93 | 86 | 91 | | 86 | | 93 | | 93 | | 6 | | -5 | | 7 | 0 |
| Fludarabine (phosphate) | MCE Cell Cycle/DNA Damage Compound Library | 107 | 95 | 98 | | 95 | | 100 | | 95 | | 101 | | 94 | | 7 | 14 |
| TH287 | MCE Cell Cycle/DNA Damage Compound Library | 107 | 103 | 107 | | 103 | | 105 | | 102 | | 47 | | 35 | | 8 | 0 |
| BMS-265246 | MCE Cell Cycle/DNA Damage Compound Library | 94 | 90 | 80 | | 90 | | 62 | | 73 | | 17 | | 30 | | 11 | 9 |
| GSK-923295 | MCE Cell Cycle/DNA Damage Compound Library | 120 | 112 | 109 | | 112 | | 108 | | 107 | | 5 | | 21 | | 11 | 14 |
| LY2835219 | MCE Cell Cycle/DNA Damage Compound Library | 104 | 103 | 102 | | 103 | | 104 | | 97 | | 43 | | 61 | | 11 | 7 |
| KU-57788 | MCE Cell Cycle/DNA Damage Compound Library | 93 | 96 | 86 | | 96 | | 95 | | 94 | | 99 | | 89 | | 12 | 31 |
| NU2058 | MCE Cell Cycle/DNA Damage Compound Library | 104 | 102 | 101 | | 102 | | 106 | | 100 | | 95 | | 99 | | 16 | 72 |
| Palbociclib (isethionate) | MCE Cell Cycle/DNA Damage Compound Library | 101 | 102 | 101 | | 102 | | 97 | | 101 | | 41 | | 61 | | 17 | 19 |
| Tenovin-1 | MCE Cell Cycle/DNA Damage Compound Library | 90 | 98 | 94 | | 98 | | 89 | | 90 | | 41 | | 46 | | 18 | 9 |
| FH535 | MCE Cell Cycle/DNA Damage Compound Library | 91 | 96 | 96 | | 96 | | 91 | | 96 | | 75 | | 79 | | 18 | 12 |
| Olaparib | MCE Cell Cycle/DNA Damage Compound Library | 95 | 93 | 91 | | 93 | | 80 | | 89 | | 81 | | 95 | | 19 | 76 |
| SGI-1027 | MCE Cell Cycle/DNA Damage Compound Library | 89 | 93 | 94 | | 93 | | 85 | | 95 | | 86 | | 91 | | 21 | 90 |
| Palbociclib | MCE Cell Cycle/DNA Damage Compound Library | 115 | 101 | 106 | | 101 | | 106 | | 112 | | 56 | | 73 | | 22 | 28 |
| Kenpaullone | MCE Cell Cycle/DNA Damage Compound Library | 107 | 94 | 105 | | 94 | | 111 | | 97 | | 86 | | 89 | | 23 | 29 |
| Pyrimethamine | MCE Cell Cycle/DNA Damage Compound Library | 109 | 102 | 112 | | 102 | | 99 | | 114 | | 105 | | 110 | | 25 | 44 |
| NG 52 | MCE Cell Cycle/DNA Damage Compound Library | 97 | 94 | 101 | | 94 | | 89 | | 95 | | 89 | | 91 | | 27 | 28 |
| GSK2606414 | MCE Cell Cycle/DNA Damage Compound Library | 91 | 94 | 82 | | 94 | | 86 | | 81 | | 83 | | 89 | | 28 | 43 |
| GSK269962A | MCE Cell Cycle/DNA Damage Compound Library | 102 | 98 | 87 | | 98 | | 79 | | 82 | | 54 | | 64 | | 31 | 48 |
| Busulfan | MCE Cell Cycle/DNA Damage Compound Library | 89 | 100 | 99 | | 100 | | 83 | | 102 | | 84 | | 89 | | 34 | 43 |
| VER-155008 | MCE Cell Cycle/DNA Damage Compound Library | 86 | 98 | 89 | | 98 | | 89 | | 96 | | 83 | | 84 | | 35 | 52 |
| P005091 | MCE Cell Cycle/DNA Damage Compound Library | 91 | 102 | 99 | | 102 | | 96 | | 101 | | 90 | | 99 | | 36 | 50 |
| PFI-4 | MCE Cell Cycle/DNA Damage Compound Library | 111 | 114 | 110 | | 114 | | 116 | | 109 | | 109 | | 108 | | 39 | 67 |
| KU-60019 | MCE Cell Cycle/DNA Damage Compound Library | 123 | 119 | 111 | | 119 | | 109 | | 108 | | 112 | | 125 | | 40 | 48 |
| Pyridostatin (hydrochloride) | MCE Cell Cycle/DNA Damage Compound Library | 101 | 101 | 101 | | 101 | | 99 | | 102 | | 103 | | 102 | | 44 | 84 |
| Betulinic acid | MCE Cell Cycle/DNA Damage Compound Library | 105 | 96 | 105 | | 96 | | 106 | | 110 | | 91 | | 90 | | 44 | 47 |
| OF-1 | MCE Cell Cycle/DNA Damage Compound Library | 98 | 101 | 108 | | 101 | | 93 | | 103 | | 87 | | 96 | | 46 | 71 |
| GSK503 | MCE Cell Cycle/DNA Damage Compound Library | 100 | 98 | 92 | | 98 | | 92 | | 94 | | 83 | | 93 | | 54 | 74 |
| GSK-5959 | MCE Cell Cycle/DNA Damage Compound Library | 97 | 93 | 94 | | 93 | | 92 | | 97 | | 88 | | 93 | | 56 | 70 |
| CGK733 | MCE Cell Cycle/DNA Damage Compound Library | 92 | 87 | 94 | | 87 | | 90 | | 89 | | 85 | | 86 | | 60 | 21 |
| UNC0642 | MCE Cell Cycle/DNA Damage Compound Library | 105 | 103 | 104 | | 103 | | 112 | | 110 | | 107 | | 98 | | 62 | 89 |
| UF010 | MCE Cell Cycle/DNA Damage Compound Library | 113 | 107 | 110 | | 107 | | 102 | | 107 | | 110 | | 110 | | 63 | 60 |
| KU-55933 | MCE Cell Cycle/DNA Damage Compound Library | 101 | 91 | 87 | | 91 | | 101 | | 94 | | 101 | | 92 | | 66 | 74 |
| Wortmannin | MCE Cell Cycle/DNA Damage Compound Library | 117 | 104 | 105 | | 104 | | 111 | | 101 | | 99 | | 97 | | 69 | 77 |
| GSK126 | MCE Cell Cycle/DNA Damage Compound Library | 105 | 108 | 110 | | 108 | | 103 | | 115 | | 93 | | 106 | | 70 | 86 |
| MN-64 | MCE Cell Cycle/DNA Damage Compound Library | 102 | 96 | 97 | | 96 | | 89 | | 87 | | 90 | | 90 | | 71 | 87 |
| ZLN005 | MCE Cell Cycle/DNA Damage Compound Library | 111 | 98 | 102 | | 98 | | 100 | | 102 | | 100 | | 102 | | 71 | 58 |
| Fotemustine | MCE Cell Cycle/DNA Damage Compound Library | 97 | 100 | 93 | | 100 | | 88 | | 100 | | 90 | | 96 | | 72 | 0 |
| EPZ-6438 | MCE Cell Cycle/DNA Damage Compound Library | 87 | 90 | 92 | | 90 | | 89 | | 85 | | 75 | | 88 | | 74 | 82 |
| BIBR 1532 | MCE Cell Cycle/DNA Damage Compound Library | 93 | 93 | 90 | | 93 | | 81 | | 91 | | 89 | | 93 | | 74 | 83 |
| Thiazovivin | MCE Cell Cycle/DNA Damage Compound Library | 114 | 109 | 111 | | 109 | | 110 | | 113 | | 102 | | 105 | | 74 | 84 |
| BRD7116 | MCE Cell Cycle/DNA Damage Compound Library | 107 | 104 | 103 | | 104 | | 101 | | 100 | | 96 | | 103 | | 78 | 72 |
| Y-27632 (dihydrochloride) | MCE Cell Cycle/DNA Damage Compound Library | 81 | 91 | 85 | | 91 | | 95 | | 91 | | 93 | | 84 | | 79 | 88 |
| ML-323 | MCE Cell Cycle/DNA Damage Compound Library | 99 | 104 | 97 | | 104 | | 102 | | 99 | | 101 | | 102 | | 81 | 89 |
| Zebularine | MCE Cell Cycle/DNA Damage Compound Library | 94 | 96 | 94 | | 96 | | 92 | | 93 | | 92 | | 94 | | 82 | 79 |
| BRD73954 | MCE Cell Cycle/DNA Damage Compound Library | 94 | 96 | 90 | | 96 | | 91 | | 95 | | 89 | | 91 | | 83 | 88 |
| EPZ004777 | MCE Cell Cycle/DNA Damage Compound Library | 92 | 103 | 93 | | 103 | | 94 | | 103 | | 102 | | 94 | | 87 | 103 |
| EPZ011989 | MCE Cell Cycle/DNA Damage Compound Library | 110 | 104 | 110 | | 104 | | 106 | | 106 | | 98 | | 99 | | 90 | 108 |
| Lomustine | MCE Cell Cycle/DNA Damage Compound Library | 96 | 97 | 104 | | 97 | | 94 | | 97 | | 96 | | 98 | | 92 | 54 |
| Vidarabine | MCE Cell Cycle/DNA Damage Compound Library | 96 | 93 | 96 | | 93 | | 93 | | 90 | | 84 | | 89 | | 93 | 92 |
| Temozolomide | MCE Cell Cycle/DNA Damage Compound Library | 91 | 85 | 99 | | 85 | | 95 | | 95 | | 91 | | 92 | | 94 | 75 |
| CDK9-IN-1 | MCE Cell Cycle/DNA Damage Compound Library | 108 | 103 | 110 | | 103 | | 103 | | 105 | | 100 | | 101 | | 94 | 85 |
| Cyclophosphamide | MCE Cell Cycle/DNA Damage Compound Library | 113 | 104 | 106 | | 104 | | 106 | | 102 | | 103 | | 101 | | 97 | 112 |
| Palifosfamide | MCE Cell Cycle/DNA Damage Compound Library | 107 | 98 | 103 | | 98 | | 105 | | 113 | | 109 | | 95 | | 97 | 101 |
| Estramustine (phosphate sodium) | MCE Cell Cycle/DNA Damage Compound Library | 104 | 98 | 101 | | 98 | | 110 | | 108 | | 103 | | 104 | | 100 | 104 |
| Folinic acid (Calcium) | MCE Cell Cycle/DNA Damage Compound Library | 96 | 95 | 85 | | 95 | | 91 | | 108 | | 99 | | 104 | | 101 | 111 |
| Tegafur | MCE Cell Cycle/DNA Damage Compound Library | 94 | 92 | 100 | | 92 | | 100 | | 98 | | 106 | | 96 | | 101 | 101 |
| UNC 669 | MCE Cell Cycle/DNA Damage Compound Library | 86 | 96 | 93 | | 96 | | 95 | | 86 | | 86 | | 83 | | 92 | 91 |
| Levomefolic acid | MCE Cell Cycle/DNA Damage Compound Library | 99 | 93 | 99 | | 93 | | 92 | | 98 | | 95 | | 91 | | 99 | 98 |
| Gemfibrozil | MCE Cell Cycle/DNA Damage Compound Library | 98 | 90 | 97 | | 90 | | 100 | | 91 | | 87 | | 92 | | 90 | 93 |
| Cytidine | MCE Cell Cycle/DNA Damage Compound Library | 104 | 92 | 95 | | 92 | | 86 | | 97 | | 95 | | 92 | | 87 | 92 |
| GSK429286A | MCE Cell Cycle/DNA Damage Compound Library | 94 | 92 | 84 | | 92 | | 93 | | 96 | | 84 | | 92 | | 80 | 87 |
| SGC707 | MCE Cell Cycle/DNA Damage Compound Library | 93 | 93 | 89 | | 93 | | 94 | | 91 | | 90 | | 88 | | 95 | 96 |
| (R)-(-)-JQ1 Enantiomer | MCE Cell Cycle/DNA Damage Compound Library | 109 | 116 | 108 | | 116 | | 99 | | 106 | | 104 | | 97 | | 82 | 99 |
| GSK2801 | MCE Cell Cycle/DNA Damage Compound Library | 96 | 95 | 97 | | 95 | | 95 | | 111 | | 95 | | 92 | | 85 | 93 |
| Trimethoprim | MCE Cell Cycle/DNA Damage Compound Library | 94 | 92 | 99 | | 92 | | 95 | | 92 | | 88 | | 85 | | 99 | 90 |
| Carboplatin | MCE Cell Cycle/DNA Damage Compound Library | 97 | 93 | 97 | | 93 | | 91 | | 93 | | 88 | | 87 | | 89 | 97 |
| UNC1215 | MCE Cell Cycle/DNA Damage Compound Library | 100 | 93 | 93 | | 93 | | 91 | | 97 | | 91 | | 102 | | 95 | 87 |
| Doxifluridine | MCE Cell Cycle/DNA Damage Compound Library | 104 | 96 | 99 | | 96 | | 102 | | 96 | | 101 | | 99 | | 100 | 98 |
| UPF 1069 | MCE Cell Cycle/DNA Damage Compound Library | 96 | 95 | 88 | | 95 | | 86 | | 87 | | 91 | | 92 | | 88 | 97 |
| Pioglitazone (hydrochloride) | MCE Cell Cycle/DNA Damage Compound Library | 104 | 88 | 102 | | 88 | | 103 | | 99 | | 99 | | 92 | | 91 | 89 |
| GSK0660 | MCE Cell Cycle/DNA Damage Compound Library | 93 | 98 | 103 | | 98 | | 99 | | 112 | | 93 | | 96 | | 80 | 99 |
| PU-WS13 | MCE Cell Cycle/DNA Damage Compound Library | 98 | 95 | 109 | | 95 | | 97 | | 99 | | 89 | | 96 | | 87 | 91 |
| Forodesine (hydrochloride) | MCE Cell Cycle/DNA Damage Compound Library | 100 | 93 | 102 | | 93 | | 99 | | 86 | | 90 | | 92 | | 96 | 97 |
| EI1 | MCE Cell Cycle/DNA Damage Compound Library | 96 | 95 | 95 | | 95 | | 89 | | 90 | | 85 | | 86 | | 81 | 91 |
| WDR5-0103 | MCE Cell Cycle/DNA Damage Compound Library | 94 | 91 | 95 | | 91 | | 90 | | 97 | | 96 | | 93 | | 89 | 98 |
| Fenofibrate | MCE Cell Cycle/DNA Damage Compound Library | 106 | 100 | 96 | | 100 | | 106 | | 106 | | 99 | | 101 | | 81 | 90 |
| XAV-939 | MCE Cell Cycle/DNA Damage Compound Library | 107 | 99 | 113 | | 99 | | 110 | | 110 | | 111 | | 121 | | 100 | 96 |
| EPZ-5676 | MCE Cell Cycle/DNA Damage Compound Library | 103 | 102 | 103 | | 102 | | 103 | | 104 | | 92 | | 99 | | 86 | 99 |
| Hydroxyurea | MCE Cell Cycle/DNA Damage Compound Library | 106 | 107 | 104 | | 107 | | 111 | | 108 | | 101 | | 104 | | 90 | 99 |
| A-366 | MCE Cell Cycle/DNA Damage Compound Library | 113 | 104 | 109 | | 104 | | 109 | | 105 | | 102 | | 98 | | 86 | 99 |
| Sirtinol | MCE Cell Cycle/DNA Damage Compound Library | 91 | 94 | 95 | | 94 | | 91 | | 99 | | 94 | | 96 | | 102 | 100 |
| Bezafibrate | MCE Cell Cycle/DNA Damage Compound Library | 100 | 95 | 95 | | 95 | | 99 | | 97 | | 103 | | 97 | | 97 | 101 |
| Wy-14643 | MCE Cell Cycle/DNA Damage Compound Library | 96 | 91 | 94 | | 91 | | 97 | | 91 | | 88 | | 92 | | 97 | 101 |
| Flumequine | MCE Cell Cycle/DNA Damage Compound Library | 95 | 90 | 98 | | 90 | | 102 | | 95 | | 94 | | 96 | | 87 | 101 |
| Tipiracil (hydrochloride) | MCE Cell Cycle/DNA Damage Compound Library | 105 | 99 | 99 | | 99 | | 104 | | 104 | | 101 | | 96 | | 101 | 102 |
| MM-102 (trifluoroacetate) | MCE Cell Cycle/DNA Damage Compound Library | 104 | 88 | 101 | | 88 | | 93 | | 102 | | 94 | | 101 | | 95 | 103 |
| TZ9 | MCE Cell Cycle/DNA Damage Compound Library | 115 | 109 | 110 | | 109 | | 108 | | 99 | | 104 | | 104 | | 96 | 105 |
| WIKI4 | MCE Cell Cycle/DNA Damage Compound Library | 101 | 90 | 102 | | 90 | | 93 | | 102 | | 99 | | 102 | | 95 | 109 |
| L189 | MCE Cell Cycle/DNA Damage Compound Library | 104 | 100 | 108 | | 100 | | 107 | | 105 | | 103 | | 100 | | 103 | 109 |
| EX-527 | MCE Cell Cycle/DNA Damage Compound Library | 106 | 105 | 112 | | 105 | | 109 | | 106 | | 107 | | 118 | | 104 | 104 |
| RG108 | MCE Cell Cycle/DNA Damage Compound Library | 106 | 101 | 98 | | 101 | | 104 | | 96 | | 98 | | 93 | | 104 | 97 |
| TBB | MCE Cell Cycle/DNA Damage Compound Library | 106 | 100 | 104 | | 100 | | 97 | | 108 | | 106 | | 105 | | 104 | 103 |
| Cisplatin | MCE Cell Cycle/DNA Damage Compound Library | 104 | 97 | 112 | | 97 | | 109 | | 99 | | 103 | | 100 | | 106 | 102 |
| Lomeguatrib | MCE Cell Cycle/DNA Damage Compound Library | 107 | 96 | 111 | | 96 | | 106 | | 111 | | 103 | | 99 | | 108 | 103 |
| TAME | MCE Cell Cycle/DNA Damage Compound Library | 106 | 114 | 110 | | 114 | | 107 | | 109 | | 106 | | 117 | | 108 | 115 |
| PFI-2 (hydrochloride) | MCE Cell Cycle/DNA Damage Compound Library | 119 | 114 | 117 | | 114 | | 116 | | 103 | | 119 | | 112 | | 109 | 108 |
| Adenine | MCE Cell Cycle/DNA Damage Compound Library | 109 | 93 | 108 | | 93 | | 108 | | 104 | | 105 | | 95 | | 110 | 109 |
| Sodium Butyrate | MCE Cell Cycle/DNA Damage Compound Library | 109 | 100 | 107 | | 100 | | 100 | | 104 | | 106 | | 103 | | 110 | 101 |
| Carmustine | MCE Cell Cycle/DNA Damage Compound Library | 102 | 99 | 105 | | 99 | | 104 | | 105 | | 100 | | 106 | | 111 | 98 |
| Streptozocin | MCE Cell Cycle/DNA Damage Compound Library | 110 | 100 | 106 | | 100 | | 105 | | 107 | | 110 | | 110 | | 112 | 115 |
| BSI-201 | MCE Cell Cycle/DNA Damage Compound Library | 114 | 100 | 104 | | 100 | | 105 | | 110 | | 100 | | 102 | | 113 | 98 |
| Adenosine | MCE Cell Cycle/DNA Damage Compound Library | 111 | 105 | 107 | | 105 | | 113 | | 109 | | 102 | | 105 | | 114 | 106 |
| INO-1001 | MCE Cell Cycle/DNA Damage Compound Library | 110 | 112 | 104 | | 112 | | 107 | | 108 | | 114 | | 124 | | 115 | 105 |
| 10-Hydroxycamptothecin | Sequoia FDA approved anti-neoplastic drug library | | | 2 | 5 | 4 | 35 | 0 | 2 | 1 | 2 | 0 | 1 | 0 | 0 | | |
| 2'-DEOXY-5-FLUOROURIDINE | Sequoia FDA approved anti-neoplastic drug library | | | 18 | 56 | 23 | 70 | 2 | 4 | 4 | 6 | 3 | 5 | 2 | 4 | | |
| 2'-Deoxy-5-methylcytidine | Sequoia FDA approved anti-neoplastic drug library | | | 104 | 98 | 108 | 97 | 109 | 97 | 104 | 102 | 102 | 80 | 114 | 107 | | |
| 5'-DEOXY-5-FLUOROCYTISINE | Sequoia FDA approved anti-neoplastic drug library | | | 95 | 97 | 92 | 103 | 98 | 98 | 99 | 108 | 99 | 107 | 104 | 107 | | |

| | | | | | | | | | | | | | |
|---|---|---|---|---|---|---|---|---|---|---|---|---|---|
| 7-EPI-TAXOL | Sequoia FDA approved anti-neoplastic drug library | 88 | 96 | 88 | 93 | 1 | 3 | 29 | 30 | 1 | 2 | 0 | 2 |
| 7-ETHYL-10-HYDROXYCAMPTOTH | Sequoia FDA approved anti-neoplastic drug library | 1 | 3 | 3 | 4 | 0 | 1 | 1 | 2 | 0 | 1 | 0 | 1 |
| A-769662 | Sequoia FDA approved anti-neoplastic drug library | 114 | 103 | 113 | 101 | 116 | 106 | 115 | 103 | 112 | 89 | 120 | 101 |
| ABIRATERONE | Sequoia FDA approved anti-neoplastic drug library | 108 | 110 | 104 | 102 | 103 | 107 | 102 | 99 | 105 | 88 | 96 | 95 |
| ABIRATERONE ACETATE | Sequoia FDA approved anti-neoplastic drug library | 105 | 101 | 110 | 107 | 99 | 100 | 104 | 104 | 98 | 95 | 98 | 120 |
| ABT263 | Sequoia FDA approved anti-neoplastic drug library | 78 | 93 | 72 | 83 | 0 | 53 | 0 | 12 | 0 | 0 | 0 | 0 |
| ABT-888 | Sequoia FDA approved anti-neoplastic drug library | 112 | 112 | 106 | 113 | 115 | 103 | 109 | 102 | 112 | 106 | 112 | 114 |
| ACLARUBICIN HCL | Sequoia FDA approved anti-neoplastic drug library | 91 | 94 | 90 | 97 | 94 | 44 | 97 | 44 | 73 | 0 | 84 | -1 |
| ADRIAMYCIN | Sequoia FDA approved anti-neoplastic drug library | 70 | 51 | 85 | 86 | 2 | 1 | 9 | 13 | 0 | 0 | 0 | 0 |
| AFATINIB | Sequoia FDA approved anti-neoplastic drug library | 93 | 87 | 99 | 91 | 97 | 89 | 93 | 91 | 82 | 48 | 81 | 63 |
| ALARELIN ACETATE | Sequoia FDA approved anti-neoplastic drug library | 103 | 101 | 106 | 105 | 107 | 104 | 101 | 99 | 105 | 114 | 102 | 116 |
| AMG-458 | Sequoia FDA approved anti-neoplastic drug library | 99 | 103 | 108 | 108 | 95 | 92 | 94 | 99 | 30 | 43 | 49 | 67 |
| ANASTROZOLE | Sequoia FDA approved anti-neoplastic drug library | 106 | 98 | 104 | 104 | 110 | 102 | 102 | 106 | 105 | 104 | 106 | 112 |
| APREPITANT | Sequoia FDA approved anti-neoplastic drug library | 108 | 97 | 110 | 101 | 107 | 113 | 101 | 106 | 113 | 98 | 107 | 101 |
| AS703026 | Sequoia FDA approved anti-neoplastic drug library | 104 | 121 | 101 | 112 | 95 | 111 | 88 | 109 | 73 | 69 | 84 | 104 |
| AT7519 | Sequoia FDA approved anti-neoplastic drug library | 106 | 107 | 107 | 104 | 102 | 99 | 105 | 99 | 5 | 1 | 61 | 74 |
| AXITINIB | Sequoia FDA approved anti-neoplastic drug library | 111 | 106 | 110 | 106 | 115 | 105 | 108 | 106 | 59 | 11 | 73 | 40 |
| AZD-4547 | Sequoia FDA approved anti-neoplastic drug library | 110 | 103 | 106 | 102 | 105 | 107 | 105 | 105 | 69 | 45 | 80 | 85 |
| AZD-6244 | Sequoia FDA approved anti-neoplastic drug library | 109 | 124 | 115 | 116 | 106 | 113 | 101 | 107 | 86 | 86 | 102 | 104 |
| AZD-8055 | Sequoia FDA approved anti-neoplastic drug library | 78 | 83 | 87 | 83 | 35 | 47 | 44 | 56 | 17 | 25 | 19 | 24 |
| BAY 60-7550 | Sequoia FDA approved anti-neoplastic drug library | 106 | 98 | 104 | 101 | 103 | 105 | 99 | 97 | 107 | 122 | 103 | 111 |
| BLEOMYCIN SULPHATE | Sequoia FDA approved anti-neoplastic drug library | 59 | 49 | 86 | 89 | 9 | 5 | 41 | 54 | 2 | 2 | 5 | 5 |
| BMS-777607 | Sequoia FDA approved anti-neoplastic drug library | 97 | 92 | 107 | 90 | 92 | 89 | 97 | 94 | 49 | 50 | 68 | 67 |
| BMS-794833 | Sequoia FDA approved anti-neoplastic drug library | 87 | 97 | 96 | 105 | 37 | 67 | 54 | 84 | 23 | 35 | 29 | 66 |
| BORTEZOMIB | Sequoia FDA approved anti-neoplastic drug library | 0 | 0 | 0 | 0 | 0 | 0 | 0 | 0 | 0 | 0 | 0 | 0 |
| BOSUTINIB Isomer 1 | Sequoia FDA approved anti-neoplastic drug library | 99 | 95 | 100 | 95 | 103 | 85 | 100 | 89 | 29 | 26 | 58 | 32 |
| BRIVANIB ALANINATE | Sequoia FDA approved anti-neoplastic drug library | 113 | 113 | 109 | 120 | 121 | 121 | 104 | 106 | 108 | 83 | 111 | 107 |
| BUSERELIN ACETATE | Sequoia FDA approved anti-neoplastic drug library | 100 | 87 | 98 | 98 | 108 | 94 | 102 | 92 | 97 | 91 | 94 | 109 |
| CANERTINIB DIHCL | Sequoia FDA approved anti-neoplastic drug library | 98 | 94 | 102 | 95 | 95 | 91 | 98 | 94 | 63 | 60 | 82 | 76 |
| CARMUSTINE | Sequoia FDA approved anti-neoplastic drug library | 100 | 106 | 110 | 102 | 108 | 101 | 107 | 103 | 110 | 134 | 109 | 56 |
| CCG-63802 | Sequoia FDA approved anti-neoplastic drug library | 96 | 122 | 107 | 114 | 103 | 116 | 102 | 111 | 87 | 33 | 99 | 69 |
| CCG-63808 | Sequoia FDA approved anti-neoplastic drug library | 111 | 113 | 113 | 113 | 114 | 110 | 103 | 108 | 108 | 67 | 106 | 106 |
| CDDO Methyl ester | Sequoia FDA approved anti-neoplastic drug library | 110 | 93 | 107 | 90 | 70 | 70 | 82 | 76 | 0 | 0 | 0 | 0 |
| CEDIRANIB | Sequoia FDA approved anti-neoplastic drug library | 89 | 101 | 98 | 108 | 95 | 103 | 99 | 102 | 70 | 66 | 91 | 86 |
| CISPLATIN | Sequoia FDA approved anti-neoplastic drug library | 111 | 122 | 108 | 114 | 117 | 108 | 104 | 107 | 117 | 113 | 108 | 109 |
| CLADRIBINE | Sequoia FDA approved anti-neoplastic drug library | 83 | 99 | 67 | 76 | 0 | 0 | 0 | 0 | 0 | 1 | 0 | 1 |
| CLOFARABINE | Sequoia FDA approved anti-neoplastic drug library | 15 | 72 | 17 | 35 | 0 | 1 | 0 | 1 | 0 | 1 | 0 | 1 |
| CP-690550 | Sequoia FDA approved anti-neoplastic drug library | 111 | 107 | 108 | 100 | 108 | 107 | 102 | 105 | 116 | 118 | 108 | 104 |
| CRIZOTINIB | Sequoia FDA approved anti-neoplastic drug library | 111 | 110 | 110 | 112 | 107 | 107 | 113 | 103 | 75 | 6 | 86 | 12 |
| CYC-116 | Sequoia FDA approved anti-neoplastic drug library | 105 | 121 | 99 | 102 | 83 | 93 | 81 | 91 | 3 | 7 | 4 | 6 |
| CYT387 | Sequoia FDA approved anti-neoplastic drug library | 105 | 101 | 105 | 103 | 101 | 106 | 107 | 104 | 58 | 50 | 87 | 72 |
| CYTARABINE | Sequoia FDA approved anti-neoplastic drug library | 80 | 92 | 77 | 76 | 13 | 30 | 14 | 14 | 1 | 2 | 3 | 2 |
| DASATINIB | Sequoia FDA approved anti-neoplastic drug library | 98 | 88 | 105 | 88 | 88 | 81 | 93 | 89 | 69 | 67 | 81 | 84 |
| Daun02 | Sequoia FDA approved anti-neoplastic drug library | 97 | 98 | 105 | 100 | 3 | 9 | 39 | 66 | 0 | 0 | 0 | 3 |
| DAUNORUBICINHCl | Sequoia FDA approved anti-neoplastic drug library | 71 | 47 | 86 | 88 | 1 | 1 | 7 | 9 | 0 | 1 | 0 | 1 |
| Dideoxycytidine | Sequoia FDA approved anti-neoplastic drug library | 112 | 109 | 115 | 101 | 106 | 101 | 104 | 104 | 119 | 102 | 109 | 114 |
| DOCETAXEL | Sequoia FDA approved anti-neoplastic drug library | 8 | 23 | 47 | 79 | 1 | 2 | 3 | 3 | 1 | 2 | 0 | 1 |
| DOVITINIB LACTATE | Sequoia FDA approved anti-neoplastic drug library | 53 | 54 | 67 | 72 | 16 | 36 | 35 | 57 | 6 | 9 | 9 | 12 |
| DOXIFLURIDINE | Sequoia FDA approved anti-neoplastic drug library | 104 | 109 | 98 | 108 | 104 | 111 | 98 | 103 | 98 | 99 | 104 | 104 |
| DOXORUBICIN HYDROCHLORIDE | Sequoia FDA approved anti-neoplastic drug library | 85 | 62 | 88 | 90 | 2 | 1 | 12 | 18 | 0 | 0 | 0 | 0 |
| ELACRIDAR | Sequoia FDA approved anti-neoplastic drug library | 99 | 108 | 104 | 106 | 108 | 102 | 103 | 100 | 97 | 95 | 95 | 104 |
| ELACRIDAR HCl | Sequoia FDA approved anti-neoplastic drug library | 112 | 102 | 105 | 98 | 108 | 99 | 105 | 99 | 102 | 93 | 102 | 98 |
| ENOCITABINE | Sequoia FDA approved anti-neoplastic drug library | 94 | 98 | 97 | 97 | 96 | 80 | 98 | 83 | 38 | 38 | 60 | 34 |
| EPIRUBICIN HYDROCHLORIDE | Sequoia FDA approved anti-neoplastic drug library | 99 | 93 | 105 | 98 | 14 | 4 | 58 | 42 | 0 | 1 | 0 | 1 |
| EPTAPLATIN | Sequoia FDA approved anti-neoplastic drug library | 93 | 101 | 103 | 97 | 92 | 89 | 97 | 89 | 6 | 66 | 15 | 85 |
| ERLOTINIB | Sequoia FDA approved anti-neoplastic drug library | 106 | 121 | 104 | 109 | 107 | 111 | 99 | 103 | 96 | 100 | 97 | 104 |
| ERLOTINIB HCl | Sequoia FDA approved anti-neoplastic drug library | 101 | 102 | 108 | 109 | 100 | 104 | 96 | 105 | 78 | 83 | 89 | 91 |
| ERLOTINIB MESYLATE | Sequoia FDA approved anti-neoplastic drug library | 94 | 95 | 102 | 93 | 90 | 93 | 101 | 90 | 90 | 79 | 95 | 80 |
| ESTRAMUSTINE SODIUM PHOSPH | Sequoia FDA approved anti-neoplastic drug library | 91 | 95 | 94 | 104 | 90 | 91 | 91 | 98 | 72 | 71 | 82 | 89 |
| ETOPOSIDE | Sequoia FDA approved anti-neoplastic drug library | 92 | 89 | 99 | 98 | 14 | 24 | 51 | 79 | 2 | 3 | 1 | 7 |
| EXEMESTANE | Sequoia FDA approved anti-neoplastic drug library | 106 | 115 | 104 | 116 | 107 | 107 | 104 | 115 | 110 | 105 | 103 | 111 |
| FASUDIL HCl | Sequoia FDA approved anti-neoplastic drug library | 108 | 102 | 106 | 101 | 104 | 99 | 103 | 105 | 102 | 97 | 110 | 103 |
| FLAVOPIRIDOL | Sequoia FDA approved anti-neoplastic drug library | 106 | 114 | 118 | 104 | 74 | 109 | 67 | 109 | 0 | 1 | 0 | 0 |
| FLUDARABINE | Sequoia FDA approved anti-neoplastic drug library | 104 | 99 | 108 | 99 | 104 | 104 | 105 | 99 | 8 | 1 | 30 | 2 |
| FLUDARABINE PHOSPHATE | Sequoia FDA approved anti-neoplastic drug library | 106 | 119 | 104 | 103 | 105 | 94 | 98 | 106 | 4 | 12 | 35 | 16 |
| FORETINIB | Sequoia FDA approved anti-neoplastic drug library | 28 | 46 | 44 | 71 | 13 | 33 | 35 | 60 | 1 | 3 | 1 | 3 |
| FORMESTANE | Sequoia FDA approved anti-neoplastic drug library | 110 | 108 | 109 | 103 | 109 | 109 | 108 | 102 | 105 | 100 | 108 | 107 |
| GDC-0941 | Sequoia FDA approved anti-neoplastic drug library | 92 | 107 | 99 | 95 | 68 | 71 | 78 | 81 | 22 | 28 | 37 | 39 |
| GEFITINIB | Sequoia FDA approved anti-neoplastic drug library | 120 | 115 | 114 | 108 | 115 | 114 | 111 | 106 | 103 | 73 | 113 | 90 |
| GEMCITABINE HYDROCHLORIDE | Sequoia FDA approved anti-neoplastic drug library | 2 | 3 | 3 | 2 | 1 | 2 | 1 | 1 | 1 | 2 | 0 | 3 |
| GOSERELIN ACETATE | Sequoia FDA approved anti-neoplastic drug library | 91 | 87 | 99 | 93 | 97 | 89 | 102 | 91 | 86 | 93 | 90 | 104 |
| GSK 2118436 | Sequoia FDA approved anti-neoplastic drug library | 100 | 93 | 104 | 92 | 102 | 97 | 106 | 96 | 97 | 89 | 95 | 106 |
| GSK-1120212 | Sequoia FDA approved anti-neoplastic drug library | 97 | 98 | 97 | 107 | 76 | 94 | 85 | 91 | 78 | 87 | 82 | 95 |
| GSK-1120212 DMSO | Sequoia FDA approved anti-neoplastic drug library | 90 | 109 | 99 | 102 | 73 | 90 | 82 | 102 | 79 | 70 | 78 | 93 |
| GSK-1904529A | Sequoia FDA approved anti-neoplastic drug library | 86 | 94 | 94 | 91 | 79 | 84 | 93 | 82 | 62 | 58 | 75 | 64 |
| GSK-2126458 | Sequoia FDA approved anti-neoplastic drug library | 39 | 46 | 54 | 66 | 16 | 22 | 30 | 39 | 4 | 8 | 7 | 7 |
| IDARUBICIN HCl | Sequoia FDA approved anti-neoplastic drug library | 5 | 4 | 13 | 16 | 0 | 0 | 0 | 0 | 0 | 0 | 0 | 0 |
| IDOXURIDINE | Sequoia FDA approved anti-neoplastic drug library | 113 | 107 | 109 | 108 | 105 | 102 | 100 | 99 | 45 | 46 | 77 | 67 |
| IMATINIB BASE | Sequoia FDA approved anti-neoplastic drug library | 95 | 98 | 103 | 93 | 89 | 99 | 99 | 94 | 96 | 91 | 103 | 91 |
| IMATINIB MESYLATE | Sequoia FDA approved anti-neoplastic drug library | 111 | 98 | 102 | 90 | 106 | 100 | 103 | 103 | 107 | 101 | 100 | 98 |
| IRINOTECAN HCl (trihydrate) | Sequoia FDA approved anti-neoplastic drug library | 108 | 96 | 104 | 110 | 102 | 100 | 98 | 104 | 22 | 21 | 52 | 70 |
| LAPATINIB | Sequoia FDA approved anti-neoplastic drug library | 106 | 104 | 107 | 103 | 99 | 107 | 100 | 99 | 100 | 116 | 100 | 107 |
| LAPATINIB TOSYLATE | Sequoia FDA approved anti-neoplastic drug library | 99 | 92 | 102 | 94 | 96 | 85 | 98 | 88 | 96 | 93 | 95 | 90 |
| LBH-589 | Sequoia FDA approved anti-neoplastic drug library | 0 | 1 | 0 | 1 | 0 | 1 | 0 | 0 | 0 | 1 | 0 | 1 |
| LDK378 | Sequoia FDA approved anti-neoplastic drug library | 106 | 94 | 101 | 94 | 100 | 93 | 97 | 88 | 77 | 32 | 79 | 42 |
| LEE011 | Sequoia FDA approved anti-neoplastic drug library | 88 | 97 | 97 | 100 | 89 | 92 | 97 | 93 | 46 | 46 | 75 | 101 |
| LENALIDOMIDE | Sequoia FDA approved anti-neoplastic drug library | 104 | 114 | 110 | 104 | 100 | 110 | 106 | 110 | 82 | 79 | 115 | 116 |
| LEUPROLIDE ACETATE | Sequoia FDA approved anti-neoplastic drug library | 105 | 98 | 111 | 93 | 110 | 97 | 107 | 97 | 119 | 97 | 109 | 105 |
| LOMEGUATRIB | Sequoia FDA approved anti-neoplastic drug library | 109 | 101 | 113 | 95 | 108 | 100 | 117 | 102 | 111 | 98 | 117 | 122 |
| LOMUSTINE | Sequoia FDA approved anti-neoplastic drug library | 109 | 105 | 114 | 105 | 115 | 105 | 112 | 102 | 129 | 101 | 115 | 83 |
| LONIDAMINE | Sequoia FDA approved anti-neoplastic drug library | 106 | 103 | 113 | 104 | 112 | 107 | 106 | 105 | 115 | 103 | 109 | 115 |
| LY-294002 | Sequoia FDA approved anti-neoplastic drug library | 103 | 121 | 101 | 105 | 109 | 111 | 105 | 103 | 82 | 73 | 88 | 84 |
| LY-404039 | Sequoia FDA approved anti-neoplastic drug library | 111 | 113 | 117 | 104 | 112 | 117 | 115 | 119 | 102 | 113 | 118 | 123 |
| LY-450139 | Sequoia FDA approved anti-neoplastic drug library | 107 | 101 | 112 | 99 | 105 | 100 | 108 | 96 | 98 | 95 | 103 | 98 |
| MASITINIB | Sequoia FDA approved anti-neoplastic drug library | 108 | 103 | 107 | 100 | 107 | 97 | 107 | 100 | 102 | 94 | 101 | 100 |
| MDV-3100 | Sequoia FDA approved anti-neoplastic drug library | 95 | 91 | 102 | 95 | 92 | 86 | 92 | 87 | 91 | 75 | 90 | 86 |
| MELPHALAN | Sequoia FDA approved anti-neoplastic drug library | 109 | 93 | 108 | 108 | 105 | 98 | 101 | 98 | 68 | 11 | 96 | 26 |
| MGCD-265 | Sequoia FDA approved anti-neoplastic drug library | 96 | 97 | 98 | 96 | 64 | 84 | 72 | 87 | 21 | 30 | 31 | 65 |
| MITOMYCIN C | Sequoia FDA approved anti-neoplastic drug library | 60 | 88 | 59 | 58 | 2 | 5 | 4 | 3 | 0 | 1 | 1 | 1 |
| MITOXANTHRONE HCl | Sequoia FDA approved anti-neoplastic drug library | 29 | 5 | 52 | 33 | 2 | 2 | 5 | 1 | 0 | 1 | 0 | 0 |
| MK-0822 | Sequoia FDA approved anti-neoplastic drug library | 104 | 99 | 107 | 104 | 104 | 100 | 100 | 99 | 100 | 101 | 104 | 99 |
| MONENSIN SODIUM | Sequoia FDA approved anti-neoplastic drug library | 98 | 102 | 103 | 99 | 5 | 7 | 12 | 5 | 1 | 0 | 1 | 1 |
| MOTESANIB | Sequoia FDA approved anti-neoplastic drug library | 108 | 113 | 110 | 102 | 103 | 101 | 105 | 100 | 100 | 97 | 104 | 103 |
| NAFARELIN ACETATE | Sequoia FDA approved anti-neoplastic drug library | 96 | 94 | 103 | 93 | 97 | 91 | 105 | 91 | 97 | 89 | 106 | 93 |
| NEDAPLATIN | Sequoia FDA approved anti-neoplastic drug library | 110 | 101 | 112 | 103 | 108 | 100 | 113 | 102 | 112 | 80 | 118 | 96 |
| NELARABINE | Sequoia FDA approved anti-neoplastic drug library | 108 | 113 | 107 | 112 | 111 | 107 | 103 | 115 | 104 | 109 | 104 | 100 |
| NERATINIB | Sequoia FDA approved anti-neoplastic drug library | 92 | 90 | 101 | 93 | 92 | 81 | 99 | 89 | 22 | 14 | 48 | 10 |
| NILOTINIB | Sequoia FDA approved anti-neoplastic drug library | 90 | 90 | 95 | 88 | 87 | 86 | 97 | 88 | 79 | 74 | 79 | 87 |
| NILOTINIB HCl | Sequoia FDA approved anti-neoplastic drug library | 99 | 91 | 102 | 96 | 96 | 91 | 98 | 94 | 88 | 70 | 89 | 92 |
| NIMUSTINE HCl | Sequoia FDA approved anti-neoplastic drug library | 114 | 117 | 109 | 112 | 114 | 118 | 109 | 99 | 112 | 105 | 110 | 42 |
| NOLATREXED 2HCl | Sequoia FDA approved anti-neoplastic drug library | 112 | 114 | 109 | 103 | 109 | 106 | 110 | 103 | 71 | 40 | 99 | 70 |
| Nullscript | Sequoia FDA approved anti-neoplastic drug library | 91 | 92 | 98 | 90 | 95 | 84 | 96 | 88 | 83 | 82 | 92 | 92 |
| NVP-BEZ235 | Sequoia FDA approved anti-neoplastic drug library | 89 | 91 | 99 | 104 | 22 | 45 | 36 | 60 | 8 | 27 | 11 | 30 |
| NVP-BGJ398 | Sequoia FDA approved anti-neoplastic drug library | 99 | 102 | 104 | 96 | 100 | 98 | 102 | 90 | 91 | 50 | 95 | 66 |
| NVP-TAE684 | Sequoia FDA approved anti-neoplastic drug library | 109 | 105 | 112 | 99 | 93 | 86 | 88 | 84 | 2 | 2 | 2 | 2 |
| O6-BENZYLGUANINE | Sequoia FDA approved anti-neoplastic drug library | 109 | 103 | 109 | 95 | 109 | 106 | 111 | 110 | 117 | 101 | 118 | 107 |
| OLAPARIB | Sequoia FDA approved anti-neoplastic drug library | 105 | 109 | 107 | 113 | 101 | 104 | 96 | 105 | 85 | 89 | 97 | 106 |
| OXALIPLATIN | Sequoia FDA approved anti-neoplastic drug library | 106 | 109 | 108 | 109 | 103 | 99 | 90 | 99 | 78 | 68 | 94 | 75 |
| PACLITAXEL | Sequoia FDA approved anti-neoplastic drug library | 8 | 41 | 92 | 104 | 1 | 2 | 9 | 28 | 2 | 3 | 1 | 4 |
| PAZOPANIB | Sequoia FDA approved anti-neoplastic drug library | 111 | 110 | 108 | 112 | 110 | 102 | 102 | 107 | 90 | 36 | 84 | 57 |
| PELITINIB | Sequoia FDA approved anti-neoplastic drug library | 102 | 101 | 104 | 102 | 93 | 87 | 99 | 91 | 17 | 8 | 35 | 9 |
| PEMETREXED Na | Sequoia FDA approved anti-neoplastic drug library | 92 | 84 | 76 | 86 | 3 | 4 | 5 | 5 | 4 | 6 | 3 | 6 |
| PF-2341066 (Crizotinib) | Sequoia FDA approved anti-neoplastic drug library | 114 | 104 | 111 | 99 | 107 | 107 | 117 | 107 | 58 | 6 | 75 | 4 |
| PIRARUBICIN | Sequoia FDA approved anti-neoplastic drug library | 86 | 80 | 94 | 98 | 9 | 4 | 24 | 14 | 0 | 1 | 0 | 1 |
| PLX-4032 | Sequoia FDA approved anti-neoplastic drug library | 98 | 88 | 99 | 88 | 98 | 85 | 96 | 86 | 90 | 104 | 91 | 92 |
| PONATINIB | Sequoia FDA approved anti-neoplastic drug library | 75 | 60 | 86 | 75 | 18 | 34 | 40 | 61 | 8 | 9 | 10 | 5 |
| PROCARBAZINE HCl | Sequoia FDA approved anti-neoplastic drug library | 104 | 105 | 101 | 110 | 105 | 105 | 104 | 108 | 112 | 108 | 109 | 111 |
| RALTITREXED | Sequoia FDA approved anti-neoplastic drug library | 4 | 7 | 7 | 8 | 2 | 3 | 4 | 4 | 5 | 6 | 4 | 7 |
| Regorafenib | Sequoia FDA approved anti-neoplastic drug library | 109 | 102 | 110 | 100 | 104 | 93 | 95 | 94 | 33 | 38 | 55 | 60 |
| RG7388 | Sequoia FDA approved anti-neoplastic drug library | 101 | 104 | 106 | 104 | 107 | 101 | 105 | 90 | 94 | 66 | 99 | 93 |
| RUBITECAN | Sequoia FDA approved anti-neoplastic drug library | 101 | 123 | 100 | 107 | 67 | 79 | 69 | 94 | 2 | 3 | 2 | 5 |

| | | | | | | | | | | | | | |
|---|---|---|---|---|---|---|---|---|---|---|---|---|---|
| SARACATINIB | Sequoia FDA approved anti-neoplastic drug library | 98 | 91 | 97 | 95 | 87 | 85 | 92 | 84 | 72 | 68 | 63 | 81 |
| SATRAPLATIN | Sequoia FDA approved anti-neoplastic drug library | 97 | 98 | 103 | 98 | 83 | 86 | 81 | 89 | 2 | 5 | 4 | 4 |
| SB-408124 | Sequoia FDA approved anti-neoplastic drug library | 114 | 117 | 110 | 105 | 120 | 116 | 112 | 104 | 143 | 121 | 117 | 120 |
| SORAFENIB TOLSYLATE | Sequoia FDA approved anti-neoplastic drug library | 76 | 92 | 77 | 93 | 21 | 44 | 41 | 64 | 23 | 48 | 28 | 69 |
| ST-836 HCl | Sequoia FDA approved anti-neoplastic drug library | 112 | 108 | 98 | 108 | 111 | 114 | 101 | 110 | 98 | 119 | 97 | 122 |
| SU-11274 | Sequoia FDA approved anti-neoplastic drug library | 102 | 105 | 107 | 94 | 89 | 91 | 89 | 90 | 73 | 50 | 89 | 87 |
| SUNITINIB | Sequoia FDA approved anti-neoplastic drug library | 89 | 98 | 92 | 97 | 25 | 43 | 42 | 66 | 13 | 21 | 19 | 40 |
| SUNITINIB MALATE | Sequoia FDA approved anti-neoplastic drug library | 72 | 79 | 80 | 80 | 19 | 35 | 41 | 60 | 10 | 14 | 14 | 25 |
| TANDUTINIB | Sequoia FDA approved anti-neoplastic drug library | 102 | 104 | 106 | 102 | 96 | 91 | 102 | 95 | 30 | 29 | 57 | 60 |
| TARIQUIDAR | Sequoia FDA approved anti-neoplastic drug library | 107 | 102 | 109 | 108 | 106 | 105 | 100 | 102 | 107 | 82 | 96 | 92 |
| TEGAFUR | Sequoia FDA approved anti-neoplastic drug library | 105 | 98 | 111 | 104 | 107 | 101 | 106 | 101 | 124 | 115 | 110 | 109 |
| TENIPOSIDE | Sequoia FDA approved anti-neoplastic drug library | 48 | 31 | 70 | 87 | 2 | 3 | 9 | 14 | 1 | 1 | 1 | 2 |
| TOK-001 | Sequoia FDA approved anti-neoplastic drug library | 105 | 104 | 114 | 105 | 111 | 105 | 114 | 107 | 104 | 80 | 112 | 100 |
| TOPOTECAN HCL | Sequoia FDA approved anti-neoplastic drug library | 74 | 99 | 82 | 108 | 1 | 3 | 3 | 6 | 0 | 1 | 0 | 1 |
| TOREMIFENE CITRATE | Sequoia FDA approved anti-neoplastic drug library | 105 | 98 | 104 | 98 | 115 | 91 | 105 | 96 | 109 | 89 | 104 | 97 |
| Trametinib | Sequoia FDA approved anti-neoplastic drug library | 98 | 94 | 104 | 92 | 97 | 74 | 92 | 84 | 56 | 61 | 66 | 77 |
| TRAMETINIB | Sequoia FDA approved anti-neoplastic drug library | 94 | 113 | 98 | 106 | 73 | 108 | 85 | 103 | 68 | 191 | 75 | 164 |
| TRIPTORELIN ACETATE | Sequoia FDA approved anti-neoplastic drug library | 97 | 93 | 99 | 93 | 100 | 89 | 103 | 91 | 101 | 81 | 94 | 88 |
| VADIMEZAN | Sequoia FDA approved anti-neoplastic drug library | 107 | 99 | 110 | 103 | 105 | 104 | 102 | 106 | 98 | 96 | 112 | 105 |
| VALRUBICIN | Sequoia FDA approved anti-neoplastic drug library | 102 | 95 | 105 | 98 | 47 | 45 | 77 | 87 | 0 | 0 | 2 | 4 |
| VANDETANIB | Sequoia FDA approved anti-neoplastic drug library | 99 | 105 | 102 | 99 | 97 | 97 | 102 | 99 | 76 | 54 | 90 | 74 |
| VATALANIB HCl | Sequoia FDA approved anti-neoplastic drug library | 110 | 102 | 106 | 99 | 111 | 99 | 107 | 97 | 120 | 102 | 116 | 105 |
| VINBLASTINE SULFATE | Sequoia FDA approved anti-neoplastic drug library | 2 | 2 | 32 | 11 | 1 | 2 | 3 | 1 | 0 | 0 | 1 | 0 |
| VINCRISTINE SULPHATE | Sequoia FDA approved anti-neoplastic drug library | 1 | 2 | 31 | 45 | 1 | 1 | 3 | 1 | 0 | 0 | 0 | 1 |
| VINDESINE SULFATE | Sequoia FDA approved anti-neoplastic drug library | 8 | 4 | 71 | 81 | 1 | 2 | 37 | 19 | 1 | 0 | 1 | 1 |
| VINORELBINE BITATRATE | Sequoia FDA approved anti-neoplastic drug library | 59 | 75 | 95 | 102 | 1 | 2 | 37 | 30 | 2 | 0 | 1 | 2 |
| VX-680 | Sequoia FDA approved anti-neoplastic drug library | 94 | 92 | 98 | 90 | 4 | 4 | 31 | 41 | 1 | 2 | 0 | 3 |
| YM155 | Sequoia FDA approved anti-neoplastic drug library | 87 | 95 | 111 | 101 | 1 | 1 | 89 | 92 | 0 | 0 | 0 | 16 |
| Zebularine | Sequoia FDA approved anti-neoplastic drug library | 98 | 91 | 103 | 97 | 102 | 98 | 103 | 96 | 100 | 86 | 97 | 105 |
| ZOSUQUIDAR | Sequoia FDA approved anti-neoplastic drug library | 96 | 94 | 98 | 88 | 93 | 90 | 87 | 86 | 75 | 88 | 89 | 86 |
